# Electrical impedance tomography–guided individualized ventilation strategy in patients with trauma-related and postoperative acute respiratory distress syndrome

**DOI:** 10.1371/journal.pone.0355609

**Published:** 2026-08-24

**Authors:** Minh Nguyen Viet, Thuy Luu Quang, Dong Trinh Van, Thu Nguyen Dang, Huyen Nguyen Thi

**Affiliations:** 1 Department of Anesthesia and Critical Care, Hanoi Medical University, Hanoi, Vietnam; 2 Center of Anesthesia and Surgical Intensive Care, Viet Duc University Hospital, Hanoi, Vietnam; 3 Department of Anesthesiology, Military Hospital 103, Vietnam Military Medical University, Hanoi, Vietnam; 4 Institute of Diagnostic and Interventional Radiology, Bach Mai Hospital, Hanoi, Vietnam; Massachusetts General Hospital Department of Anesthesia Critical Care and Pain Medicine, UNITED STATES OF AMERICA

## Abstract

**Objective:**

Optimal positive end-expiratory pressure (PEEP) selection in acute respiratory distress syndrome (ARDS) is challenging because of marked regional lung heterogeneity. Electrical impedance tomography (EIT) provides bedside assessment of regional ventilation and may support individualized ventilation by informing PEEP titration, but evidence in trauma- and postoperative-associated ARDS remains limited.

**Methods:**

In this single-center randomized controlled trial, 86 adults with moderate-to-severe trauma- or postoperative-associated ARDS were randomized to an EIT-guided individualized ventilation strategy (n = 43) or a lower-PEEP/FiO_2_ strategy (n = 43). Four patients were transferred early before analyzable follow-up data were collected; therefore, the modified intention-to-treat population included 42 patients in the EIT group and 40 in the control group. In the EIT group, the multicomponent strategy included EIT monitoring, recruitment maneuvers, decremental PEEP titration, repeated reassessment, and individualized PEEP selection based on the intersection of regional collapse and overdistension curves. The co-primary physiologic outcomes were PaO₂/FiO₂ and static respiratory system compliance (Cstat), assessed longitudinally over 3 days. Secondary outcomes included mechanical power (MP), Sequential Organ Failure Assessment (SOFA) scores, ventilator-free days, ICU length of stay, barotrauma, and 28-day mortality. Prespecified exploratory analyses evaluated physiologic responses according to pulmonary contusion status. This study was powered for physiologic endpoints rather than clinical outcomes.

**Results:**

PaO₂/FiO₂ improved in both groups, with greater increases in the EIT group during Days 1–3 (Sidak-adjusted p < 0.05). Overall Cstat trajectories were similar between groups. The EIT-guided individualized ventilation strategy was associated with improved oxygenation without increases in plateau pressure or driving pressure. No differential temporal pattern in MP normalized to predicted body weight (MP/PBW) was observed, although overall values were modestly higher in the EIT group. Secondary clinical outcomes were broadly similar between groups. Exploratory analyses suggested greater SOFA score reduction and shorter ICU length of stay in the EIT group. Duration of mechanical ventilation, ventilator-free days, and 28-day mortality were similar between groups. Exploratory subgroup analyses suggested selected later improvements in Cstat among patients without pulmonary contusion, whereas MP/PBW was modestly higher in the EIT group across subgroups.

**Conclusions:**

In trauma- and postoperative-associated ARDS, an EIT-guided individualized ventilation strategy was associated with improved oxygenation compared with a lower-PEEP/FiO₂ strategy, while maintaining plateau and driving pressures within lung-protective ranges. These findings suggest the physiological feasibility of this strategy, but do not establish the independent effect of EIT monitoring or the underlying mechanistic pathway. Larger multicenter studies are needed to determine mechanisms and effects on patient-centered outcomes.

## Introduction

Acute respiratory distress syndrome (ARDS) remains a significant cause of morbidity and mortality in critically ill patients with highly variable outcomes despite lung-protective ventilation strategies [1,2]. This variability reflects substantial heterogeneity in lung injury and respiratory mechanics, highlighting limitations of uniform, population-based ventilatory approaches [3,4]. Although positive end-expiratory pressure (PEEP) is central to ARDS management, reliance on global PEEP/FiO₂ tables may inadequately capture regional lung behavior, underscoring the need for strategies that incorporate spatial information into individualized ventilation, including PEEP selection [5–9].

Electrical impedance tomography (EIT) is a noninvasive bedside imaging technique that provides real-time assessment of regional ventilation distribution, enabling visualization of collapse and overdistension during PEEP titration [10–14]. Physiological studies and recent randomized trials have shown that EIT-guided individualized ventilation strategies, commonly involving PEEP titration, can improve oxygenation and respiratory mechanics, and reduce potentially injurious ventilatory load compared with conventional strategies [15–19]. However, most prior studies enrolled etiologically heterogeneous ARDS populations, predominantly related to sepsis or pneumonia, limiting insight into the effects of EIT-guided individualized ventilation strategies in specific ARDS phenotypes. ARDS following trauma or major surgery represents a distinct clinical context. It is characterized by pronounced regional heterogeneity due to dependent atelectasis, pleural effusions, pulmonary contusion, and altered chest wall mechanics [20–23]. Both trauma- and postoperative-associated ARDS share prominent mechanical heterogeneity driven by dependent atelectasis, pleural effusions, and altered chest wall mechanics, distinguishing them from predominantly inflammatory medical ARDS. In this setting, global indices such as PaO₂/FiO₂ or static respiratory system compliance (Cstat) may incompletely reflect regional recruitability. PEEP/FiO₂ tables derived largely from medical ARDS cohorts may therefore be suboptimal. Despite increasing interest in individualized ventilation strategies, evidence for EIT-guided individualized ventilation strategies remains limited in trauma- and postoperative-associated ARDS, a population characterized by distinct mechanical and structural heterogeneity. In particular, the influence of traumatic lung injury patterns, such as pulmonary contusion, on physiologic responses to PEEP remains poorly understood.

Accordingly, we conducted a randomized controlled trial comparing an EIT-guided individualized ventilation strategy with a lower-PEEP/FiO_2_ strategy in patients with moderate-to-severe trauma- and postoperative-associated ARDS. This trial was designed to examine the effects of this EIT-guided individualized ventilation strategy on oxygenation and respiratory mechanics, and to explore whether physiological responses differed according to pulmonary contusion status.

## Materials and methods

### Study design and ethical approval

This single-center randomized controlled trial was conducted at Viet Duc University Hospital, Hanoi, Vietnam, between 2025 and 2026. The protocol was approved by the Institutional Review Board of Hanoi Medical University (No. 1879/GCN-HMUIRB). Written informed consent was obtained from patients or their legal surrogates. The study complied with the Declaration of Helsinki and was reported in accordance with CONSORT guidelines and was registered at ClinicalTrials.gov (NCT07313644).

### Study population

Adult patients (≥18 years) receiving invasive mechanical ventilation in the surgical intensive care unit were screened. Patients with trauma- or postoperative-associated moderate to severe ARDS, defined according to the 2023 Global Definition of ARDS, were eligible.

Exclusion criteria included age > 90 years; severe acute brain injury or stroke (Glasgow Coma Scale <8); untreated or clinically unstable pneumothorax or pneumomediastinum, defined as ongoing air leak or radiographic progression despite appropriate chest drainage; end-stage disease or expected survival <7 days; conditions requiring prolonged ventilation (e.g., Guillain–Barré syndrome or cervical spinal cord injury); prior use of advanced respiratory therapies (extracorporeal membrane oxygenation, inhaled nitric oxide, prone positioning, or high-frequency ventilation); pregnancy or breastfeeding; skin lesions at electrode sites; implanted electrical devices interfering with EIT; known allergy to electrode materials; refusal of consent; or concurrent enrollment in another interventional trial.

Patients with chest trauma, including pulmonary contusion and stabilized pneumothorax after appropriate drainage, were eligible for inclusion.

### Randomization and study groups

Patients were randomized 1:1 to the EIT group or the control group using permuted block randomization with variable block sizes, with the allocation sequence generated by an independent investigator using a computer-based random number list. Group assignments were concealed in sequentially numbered, opaque, sealed envelopes and revealed only after written informed consent and confirmation of eligibility, when the attending physician opened the next envelope in numerical order. Due to the nature of the intervention, treating clinicians were not blinded to group allocation.

### Intervention

#### EIT group.

The EIT-guided individualized ventilation strategy comprised EIT monitoring, recruitment maneuvers, decremental PEEP titration, repeated reassessment, and individualized PEEP selection. EIT was performed using a commercial system (Enlight 2100, Timpel, São Paulo, Brazil). A 32-electrode belt was positioned circumferentially around the thorax at the 4th–6th intercostal space following a diaphragm-based positioning method [24,25]. Patients were deeply sedated (Richmond Agitation–Sedation Scale ≤−3) without spontaneous breathing, and airway secretions were adequately cleared.

A recruitment maneuver was performed using pressure-controlled ventilation with inspiratory pressure 15 cmH₂O above PEEP and PEEP 24 cmH₂O (maximum airway pressure ≤40 cmH₂O) for 30 seconds at FiO₂ 1.0. A decremental PEEP titration followed, starting at 24 cmH₂O and decreasing in 2 cmH₂O steps to a minimum of 6 cmH₂O or until SpO₂ ≤ 80% with each level maintained for 30 seconds. This duration was chosen to allow stabilization of regional ventilation signals, consistent with prior EIT-based decremental PEEP titration protocols [26].

Protocol-selected PEEP was defined as the level at which the cumulative regional collapse and overdistension curves intersected, according to the manufacturer’s standardized EIT algorithm [6,7]. The selected PEEP level was independently confirmed by two blinded investigators. An example of an individualized EIT-based PEEP titration curve is shown in S1 Fig. Following titration, a recruitment maneuver was repeated, and PEEP was set at the protocol-selected level for at least 12 hours.

PEEP reassessment within the EIT-guided individualized ventilation strategy was performed according to protocol on Days 1–3 in all patients with available follow-up in the EIT group. Repeat titration was planned on Days 1–3 according to protocol, with predefined safety criteria (e.g., hemodynamic instability or barotrauma). PEEP reduction (2 cmH₂O every 4–8 hours) was considered when PaO₂/FiO₂ exceeded 200 with FiO₂ < 0.5; deterioration prompted return to the protocol-selected PEEP with repeat recruitment if needed.

PEEP titration was discontinued for sustained mean arterial pressure decrease >20 mmHg, SpO₂ < 88%, or new arrhythmias, after which prior ventilator settings were restored.

#### Control group.

Sedation management was identical. PEEP was applied according to the lower-PEEP/FiO_2_ strategy of the ARDS Network ALVEOLI trial, using predefined PEEP–FiO₂ combinations [27]; the exact table used in this study is provided in S1 Table. Routine recruitment maneuvers were not performed. This approach is consistent with contemporary ESICM guidelines, which suggest against the routine use of brief high-pressure recruitment maneuvers in ARDS due to the absence of demonstrated mortality benefit [28].

Adjunctive therapies: Prone positioning was considered for PaO₂/FiO₂ < 150 mmHg with PEEP >10 cmH₂O and FiO₂ > 0.6, and was maintained for up to 16 hours per day [29]. Extracorporeal membrane oxygenation was considered as rescue therapy if oxygenation targets were not achieved.

Other respiratory therapies: Maintenance ventilation was delivered using volume-controlled ventilation as part of a lung-protective strategy; pressure-controlled ventilation was used transiently for standardized recruitment and EIT-based PEEP titration maneuvers, targeting a plateau pressure (Pplat) ≤30 cmH₂O, tidal volume (VT) of 4–6 mL/kg predicted body weight (PBW), and respiratory rate (RR) ≤35 breaths/min, adjusted to maintain arterial pH ≥ 7.30. The inspiratory-to-expiratory ratio was set between 1:1 and 1:3. Oxygenation targets were PaO₂ 55–80 mmHg or SpO₂ 88–95%.

### Data collection and outcomes

The co-primary physiologic outcomes were PaO₂/FiO₂ and Cstat, assessed longitudinally over the predefined study period (Days 0–3). Analyses focused on repeated measurements over time to evaluate temporal trajectories and between-group differences. Changes from baseline were analyzed as supportive measures to aid interpretation of physiologic responses. MP (J/min) was a prespecified secondary physiologic endpoint. MP was calculated as [5,30]:


$$\begin{array}{c} \text{MP }=\;\text{0}.\text{098 }\times \text{ VT }\times \text{ RR }\times \;(\text{PIP }-\;\text{0}.\text{5}\;\times \text{ Pdriv}) \end{array}$$

where PIP is peak inspiratory pressure. Driving pressure (Pdriv) was defined as Pplat minus total PEEP (PEEPtot). PEEPtot was measured under passive conditions using an end-expiratory hold. Pplat was measured during an end-inspiratory pause. PIP was used to calculate MP as previously described in clinical studies, acknowledging that this incorporates resistive components and may overestimate alveolar stress. However, this approach was consistently applied across groups and time points, thereby preserving internal validity. At each predefined study time point, ventilatory variables recorded included applied PEEP, Pplat, Pdriv, Cstat, and MP.

Secondary outcomes included 28-day mortality, ventilator-free days at day 28, ICU length of stay, barotrauma, use of rescue therapies, and Sequential Organ Failure Assessment (SOFA) scores. To account for differences in body size, MP was additionally normalized to PBW (MP/PBW), calculated based on patient height and sex using standard formulas. MP/PBW was analyzed as a supportive physiologic metric. Weaning success was defined as sustained liberation from mechanical ventilation for at least 48 hours without the need for reintubation. Patients who died before Day 28 were assigned zero ventilator-free days.

### Statistical analysis

Sample size estimation was based on a two-sided comparison of independent means using Day 1 PaO₂/FiO₂, with α = 0.05, 90% power, and allowance for 20% attrition. Continuous variables are presented as mean ± SD or median (IQR), and categorical variables as counts (percentages). Between-group comparisons used Student’s *t* test or Mann–Whitney U test for continuous variables and chi-square or Fisher’s exact test for categorical variables, as appropriate. The primary analysis population was a modified intention-to-treat population, defined as all randomized patients who received the allocated intervention and had at least one post-randomization outcome assessment available. Patients transferred before collection of analyzable follow-up data were excluded. Non-repeated secondary outcomes were analyzed using complete-case analysis. Longitudinal outcomes during Days 0–3 were analyzed using linear mixed-effects models with fixed effects for treatment group, time, and the group-by-time interaction, with subject-specific random intercepts and an autoregressive covariance structure. Model assumptions were assessed by inspection of residual distributions and diagnostic plots. This approach used all available repeated measurements while accounting for within-subject correlation. Sidak-adjusted post hoc comparisons were applied where appropriate. No imputation was performed after death, transfer, or loss to follow-up. Subgroup analyses were exploratory. A two-sided *p* < 0.05 was considered statistically significant. Analyses were performed using SPSS version 27.0 (IBM Corp., Armonk, NY, USA).

## Results

**Fig 1:**
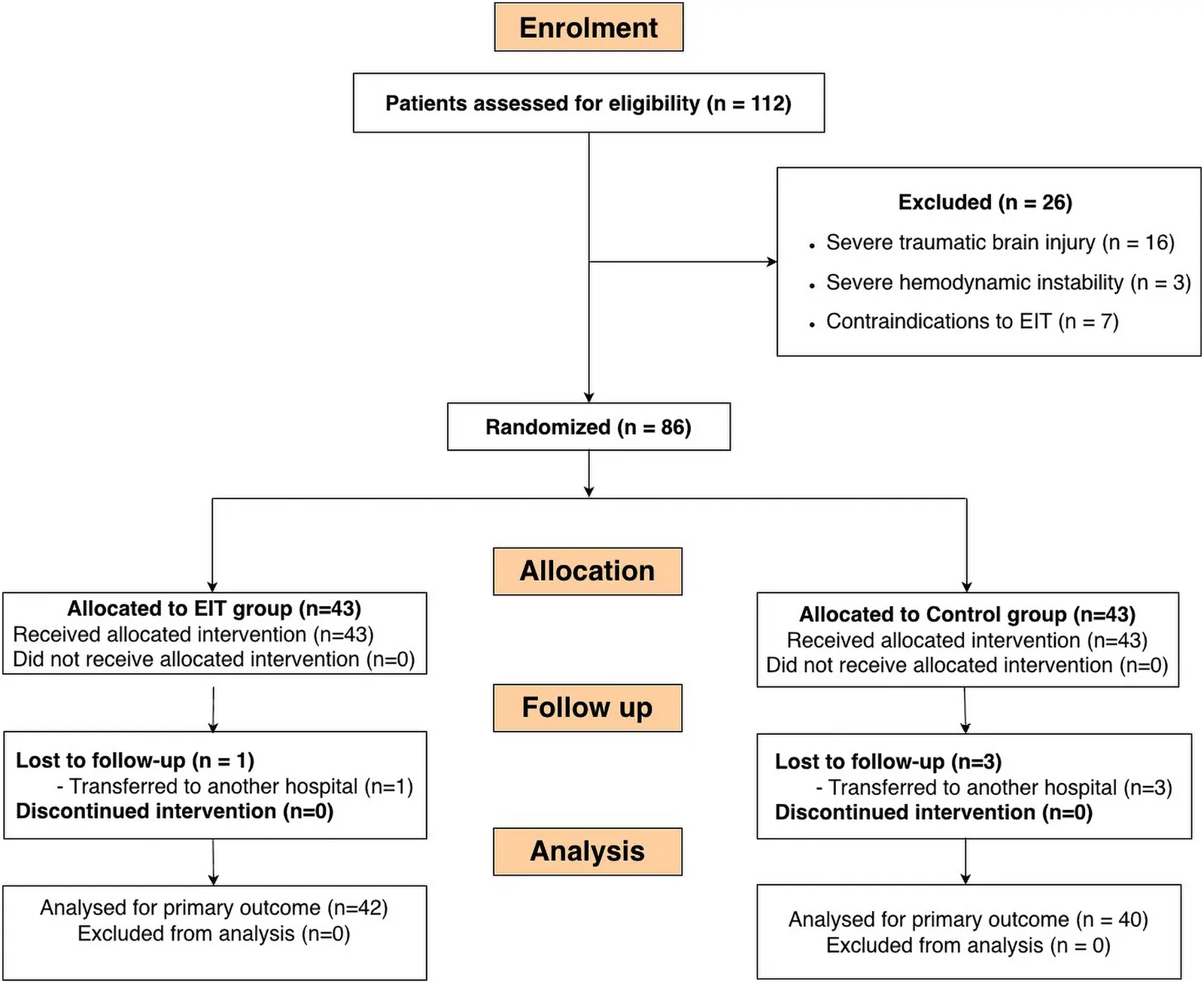
CONSORT Participant flow diagram.

Participant flow through the trial is shown in Fig 1. A total of 112 patients were assessed for eligibility, of whom 26 were excluded because of severe traumatic brain injury (n = 16), severe hemodynamic instability (n = 3), or contraindications to electrical impedance tomography (n = 7). The remaining 86 patients were randomized, with 43 assigned to the EIT group and 43 to the control group, and all received the allocated intervention. During follow-up, four patients were transferred early to other hospitals and were not further followed, including one in the EIT group and three in the control group. Accordingly, the modified intention-to-treat population included 82 patients, comprising 42 patients in the EIT group and 40 patients in the control group. Protocol adherence was high in the intervention arm: all analyzed patients underwent initial PEEP titration within the EIT-guided individualized ventilation strategy on Day 0, and repeat titration was performed on Days 1–3 in all patients with available follow-up.

### Patient characteristics

Baseline demographic and clinical characteristics were comparable between the two groups (Table 1). Four randomized patients were lost to follow-up before outcome assessment and were not included in the analysis.

**Table 1:** Baseline characteristics of the study population.

Variables	EIT group (n = 42)	Control group (n = 40)	p value
**Demographic and clinical characteristics**			
Age, years	54.0 ± 17.1	57.4 ± 18.8	0.401^α^
Male sex, n (%)	37.0 (88.1)	29.0 (72.5)	0.075^β^
Height, cm	167.0 (162, 171)	167.5 (158, 172)	0.907^γ^
Weight, kg	66.6 ± 7.9	64.9 ± 12.6	0.461^α^
Body mass index, kg/m²	24.0 ± 1.9	23.4 ± 2.8	0.275^α^
APACHE II score	12.0 (10, 15)	13.0 (9, 17)	0.485^γ^
SOFA score	9.2 ± 3.0	10.4 ± 3.2	0.107^α^
**Comorbidities**			
Hypertension, n (%)	11.0 (26.2)	12.0 (30.0)	0.701^β^
Diabetes mellitus, n (%)	6.0 (14.3)	6.0 (15.0)	0.927^β^
Chronic liver disease, n (%)	3.0 (7.1)	5.0 (12.5)	0.447^β^
Other comorbidities, n (%)	6.0 (14.3)	6.0 (15.0)	0.927^β^
**ARDS severity**			
Moderate ARDS, n (%)	32.0 (76.2)	35.0 (87.5)	0.185^β^
Severe ARDS, n (%)	10.0 (23.8)	5.0 (12.5)	
**Clinical context**			
Duration of mechanical ventilation before randomization, days	1.0 (0, 2)	1.0 (0, 2.5)	0.915^γ^
Postoperative status, n (%)	13.0 (31.0)	12.0 (30.0)	0.925^β^
Trauma-related ARDS, n (%)	29.0 (69.0)	28.0 (70.0)	
Trauma characteristics (EIT group n = 29; Control group n = 28)*			
ISS score	33.3 ± 9.4	28.7 ± 10.3	
Head/neck injury, n (%)	19.0 (65.5)	8.0 (28.6)	
Chest injury, n (%)	23.0 (79.3)	26.0 (92.9)	
Abdominal injury, n (%)	12.0 (41.4)	11.0 (39.3)	
Extremities or pelvic injury, n (%)	10.0 (34.5)	11.0 (39.3)	

Data are presented as mean ± SD or median (IQR). ARDS, acute respiratory distress syndrome; APACHE II, Acute Physiology and Chronic Health Evaluation; ISS, Injury Severity Score.

* Trauma-related variables were assessed only in patients with trauma-related ARDS. Percentages are calculated within the trauma subgroup. Between-group comparisons were not prespecified for trauma characteristics.

αStudent’s t-test; ^β^ Chi-Square Test; ^γ^ Mann-Whitney Test.

### Oxygenation

**Fig 2:**
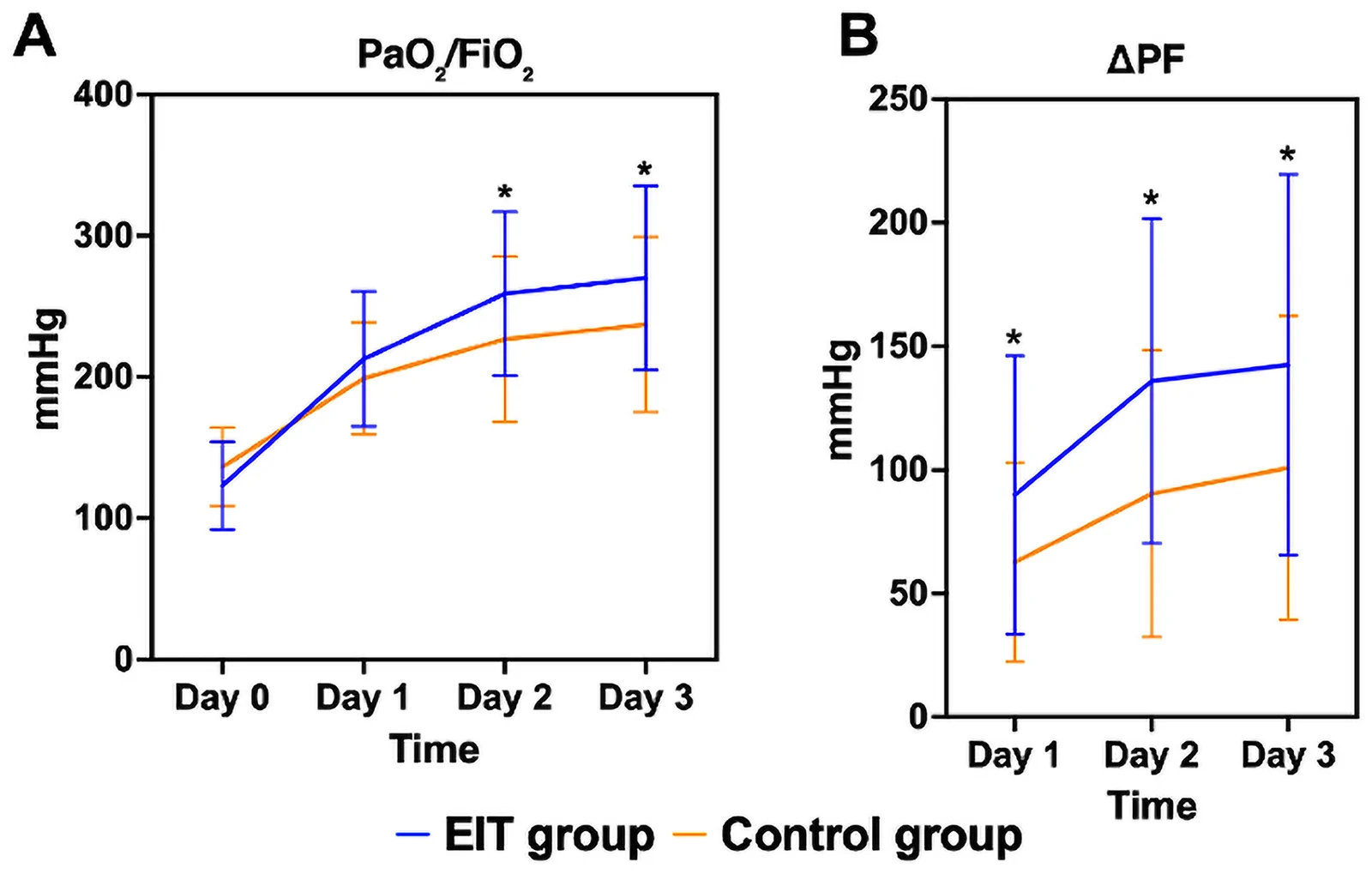
Oxygenation. Data are presented as mean ± SD. Trajectory of PaO₂/FiO₂ over time (A), change from baseline in PaO₂/FiO₂ (ΔPF; B). Longitudinal changes were analyzed using linear mixed-effects models with fixed effects for group, time, and group-by-time interaction, with Sidak-adjusted post hoc comparisons where applicable. *p < 0.05 for between-group comparisons.

PaO₂/FiO₂ changed over time in the overall cohort (linear mixed-effects model, main effect of time: F(3,153.9)=65.21, p < 0.001; Fig 2A). A group-by-time interaction was observed (F(3,153.9)=3.66, p = 0.014). PaO₂/FiO₂ values were similar between groups at Day 0 (145.8 ± 34.5 vs. 159.2 ± 35.8; p = 0.224) and Day 1 (212.9 ± 48.0 vs. 199.0 ± 39.5; p = 0.224). On Day 2 and Day 3, PaO₂/FiO₂ values were higher in the EIT group than in the control group (Day 2: 258.9 ± 58.3 vs. 226.7 ± 58.5, p = 0.005; Day 3: 267.9 ± 63.9 vs. 237.2 ± 62.2, p = 0.007; Sidak-adjusted).

Change in PaO₂/FiO₂ (ΔPF) increased over time (time effect: F(3,146.5)=61.67, p < 0.001), with a significant group-by-time interaction (F(3,146.5)=3.40, p = 0.019). Overall values were higher in the EIT group than in the control group (group effect: F(1,83.1)=11.75, p = 0.001). Between-group differences were observed at Day 1 (90.0 ± 56.4 vs. 62.7 ± 40.3 mmHg; p = 0.024), Day 2 (136.0 ± 65.7 vs. 90.4 ± 58.1 mmHg; p < 0.001), and Day 3 (142.6 ± 77.1 vs. 100.9 ± 61.6 mmHg; p = 0.001; Fig 2B).

**Fig 3:**
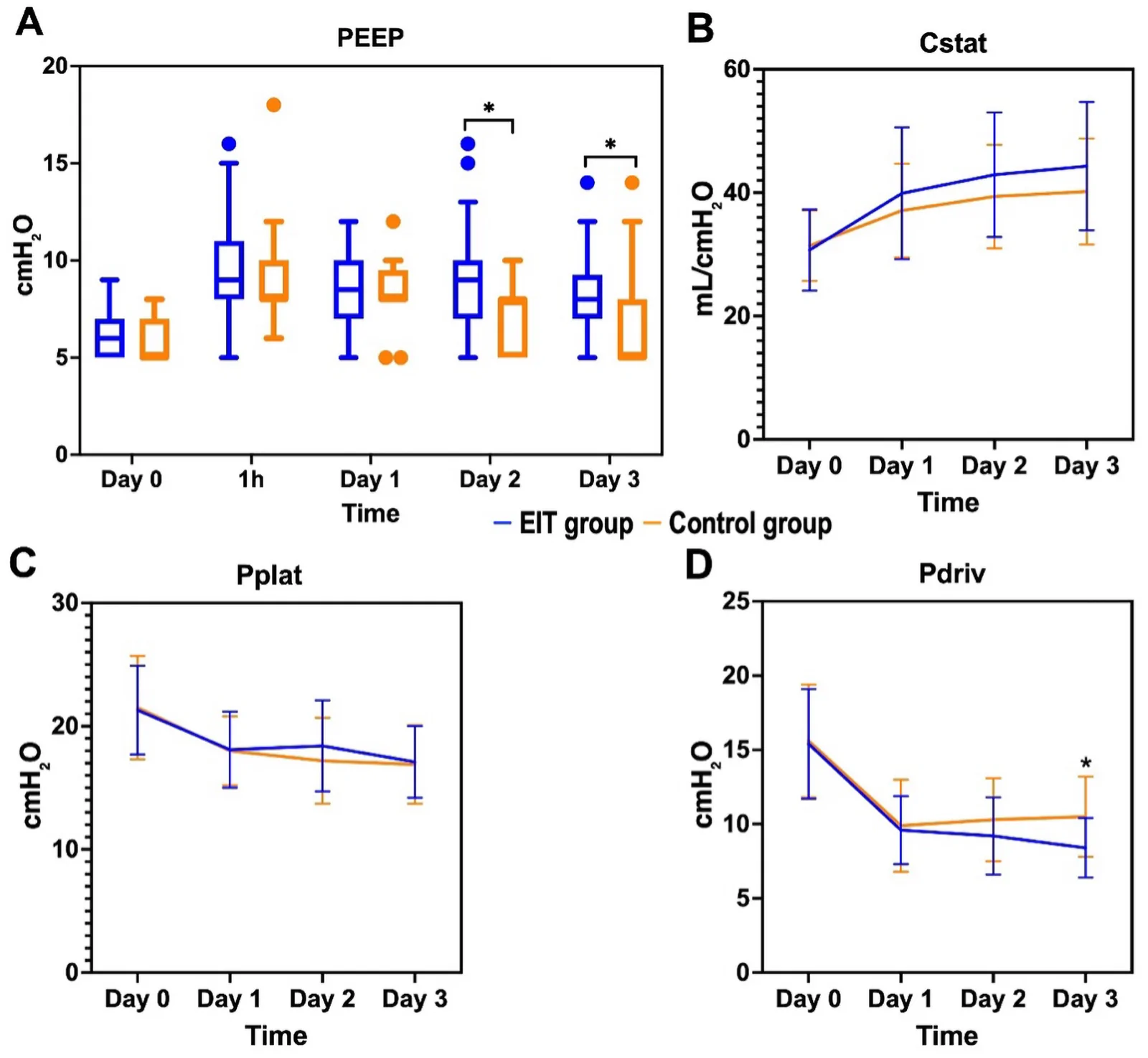
Respiratory mechanics. Positive end-expiratory pressure (PEEP; A), static respiratory system compliance (Cstat; B), plateau pressure (Pplat; C), and driving pressure (Pdriv; D). Data are presented as median (IQR) or mean ± SD, as appropriate. Longitudinal changes were analyzed using linear mixed-effects models with fixed effects for group, time, and group-by-time interaction, with Sidak-adjusted post hoc comparisons where applicable. *p < 0.05 for between-group comparisons.

PEEP values were non-normally distributed and are presented as median (IQR). Median PEEP was similar between groups at baseline, 1 hour, and Day 1. Higher values were observed in the EIT group on Day 2 and Day 3. In the linear mixed-effects model, group (F = 10.57, p = 0.002), time (F = 22.54, p < 0.001), and group-by-time interaction (F = 10.42, p < 0.001) were associated with PEEP. Post hoc comparisons showed higher PEEP levels in the EIT group on Day 2 (mean difference 2.17 cmH₂O, p < 0.001) and Day 3 (mean difference 1.83 cmH₂O, p < 0.001), but not at earlier time points (Fig 3A).

Cstat increased over time in both groups (time effect: F(3,163.6) = 29.13, p < 0.001), rising from 34.0 ± 7.4 to 44.4 ± 10.5 mL/cmH₂O in the EIT group and from 32.7 ± 6.0 to 40.3 ± 8.7 mL/cmH₂O in the control group by Day 3. There was no evidence of a group-by-time interaction (F(3,163.6) = 0.65, p = 0.587), and the overall between-group effect did not reach statistical significance (F(1,80.4) = 3.58, p = 0.062). Sidak-adjusted between-group comparisons at individual time points were not significant (all p > 0.05; Fig 3B).

Pplat decreased over time in both groups (time effect: F(3,163.6)=19.57, p < 0.001). No group-by-time interaction was observed (F(3,163.6)=1.37, p = 0.253), and no overall between-group difference was detected (F(1,80.4)=0.39, p = 0.536). Sidak-adjusted comparisons at each time point did not identify significant between-group differences (all p > 0.05; Fig 3C).

Pdriv changed over time in both groups (time effect: F(3,157.7)=4.74, p = 0.003). A group-by-time interaction was observed (F(3,157.7)=2.80, p = 0.042). Between-group differences were not identified at baseline, Day 1, or Day 2 (all p > 0.05). At Day 3, driving pressure was lower in the EIT group than in the control group (8.4 vs. 10.6 cmH₂O; Sidak-adjusted p < 0.001; Fig 3D).

MP/PBW changed over time in both groups (time effect: F(3,131.5)=6.14, p = 0.001), from 0.252 ± 0.095 to 0.225 ± 0.059 J/min/kg in the EIT group and from 0.232 ± 0.084 to 0.188 ± 0.065 J/min/kg in the control group by Day 3. No group-by-time interaction was observed (F(3,131.5)=0.57, p = 0.633). Overall values were higher in the EIT group (F(1,80.6)=5.73, p = 0.019), with no between-group difference at baseline (p = 0.221) and higher values from Day 1 onward (all p < 0.05).

### Clinical outcomes and adjunctive therapies

Clinical outcomes and use of adjunctive therapies are summarized in Table 2. Exploratory secondary outcomes showed larger reductions in SOFA scores at later time points and shorter ICU length of stay in the EIT group. Other clinical outcomes, including weaning success, ventilator-free days, duration of mechanical ventilation, hospital length of stay, and 28-day mortality, were similar between groups, although ICU length of stay was shorter in the EIT group. No barotrauma events were observed in either group during the study period. The use of adjunctive therapies was limited and balanced between groups; no patients received systemic corticosteroids or ECMO, and the frequency of prone positioning and neuromuscular blockade did not differ between groups.

**Table 2:** Clinical outcomes and adjunctive therapies.

Variable	EIT group (n = 42)	Control group (n = 40)	p value
**Clinical Outcomes**			
ΔSOFA score, Day 2	−2 (−4, −1)	−1 (−2, 0)	**0.023** ^**γ**^
ΔSOFA score, Day 3	−3 (−5, −1)	−2 (−2, 0)	**0.024** ^**γ**^
Weaning success, n (%)	32.0 (76.2)	28.0 (70.0)	0.527^α^
Ventilator-free days at day 28, days	20.0 (3, 22)	14.0 (0, 22.5)	0.474^**γ**^
Duration MV, days	11.5 (8, 17)	13.0 (7.5, 21)	0.524^**γ**^
ICU length of stay, days	8.5 (6, 14)	12.0 (8, 19)	**0.039** ^**γ**^
Hospital length of stay, days	20.0 (15, 25)	22.0 (14.5, 32.5)	0.281^**γ**^
28-day mortality, n (%)	9.0 (21.4)	12.0 (30.0)	0.374^α^
**Adjunctive therapies**			
Prone positioning, n (%)	1.0 (2.4)	2.0 (5.0)	0.611^δ^
Neuromuscular blockade, n (%)	7.0 (16.7)	7.0 (17.5)	0.92^α^

**Data are presented as median (IQR) or n (%).**

**SOFA**, Sequential Organ Failure Assessment; **ICU**, intensive care unit; **MV**, mechanical ventilation; **ECMO**, extracorporeal membrane oxygenation.

ΔSOFA represents the change from baseline (Day 0).

γMann-Whitney Test; ^α^ Chi-Square Test; ^δ^ Fisher’s Exact Test.

### Subgroup analyses

**Fig 4:**
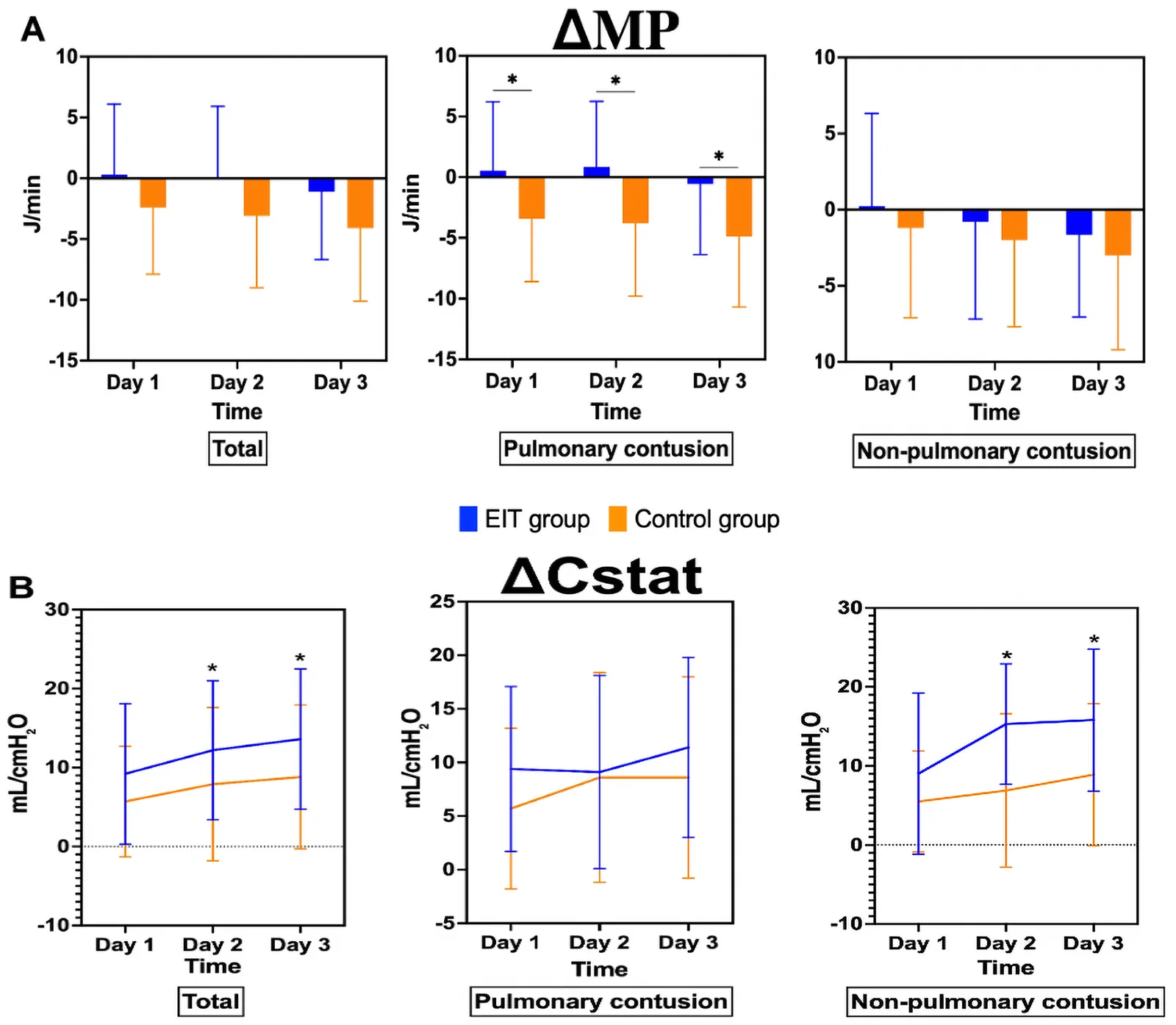
Changes in MP and Cstat according to pulmonary contusion status. (A) ΔMP and (B) ΔCstat from baseline to Days 1–3 in the overall cohort and stratified by pulmonary contusion. Data are presented as mean ± SD. Longitudinal changes were analyzed using linear mixed-effects models with fixed effects for group, time, and group-by-time interaction, with Sidak-adjusted post hoc comparisons where applicable. *p < 0.05 for between-group comparisons.

Pulmonary contusion was defined on admission chest computed tomography. Patients were classified as having pulmonary contusion or no pulmonary contusion, the latter including postoperative ARDS and trauma-related ARDS without CT evidence of pulmonary contusion. Subgroup analyses according to pulmonary contusion status were exploratory and hypothesis-generating because the study was not powered for subgroup comparisons. PEEP changed over time and was higher in the EIT group at later time points. Neither the time-by-contusion interaction nor the group-by-time-by-contusion interaction was significant, indicating similar temporal PEEP patterns across subgroups. Subgroup-specific estimated marginal means are shown in S2 Table. ΔMP showed a broadly similar pattern across subgroups. In contrast, selected later differences in ΔCstat were observed among patients without pulmonary contusion, whereas no clear between-group differences were identified in patients with pulmonary contusion. Detailed subgroup results, including fixed effects and Sidak-adjusted pairwise comparisons, are presented in S3 Table, S4 Table, and Fig 4.

## Discussion

In this randomized controlled trial of moderate-to-severe trauma- and postoperative-associated ARDS, an EIT-guided individualized ventilation optimization strategy was associated with greater improvement in oxygenation than a lower-PEEP/FiO₂ strategy. Measurements were obtained after post-titration stabilization. The intervention incorporated EIT monitoring, recruitment maneuvers, decremental PEEP titration, repeated reassessment, and individualized PEEP selection. These effects were achieved without increases in Pplat or Pdriv. MP/PBW trajectories were similar over time, although overall values were modestly higher in the EIT group. Secondary clinical outcomes were broadly similar between groups, and exploratory findings related to SOFA score reduction and ICU length of stay should be interpreted cautiously because the trial was powered for physiologic rather than clinical endpoints. Overall, these findings suggest the feasibility of an EIT-guided individualized ventilation strategy in this population.

In our study, PaO₂/FiO₂ improved in the EIT group from baseline on Day 1 but did not differ significantly from the control group at this early time point. This contrasts with studies by Zhao and Van Trung, which reported significant improvements in oxygenation within 24 hours of EIT-based PEEP titration [8,31]. One possible explanation is differences in study populations, Van Trung et al enrolled a higher proportion of patients with severe ARDS, with the observed benefit largely concentrated in this subgroup. By contrast, the proportion of severe ARDS was lower in our cohort, similar to the study by He et al., who also did not observe early absolute improvements in PaO₂/FiO₂ [9]. Nevertheless, ΔPF was consistently greater in the EIT group from Day 1 onward, with sustained and significant differences at Days 2 and 3 and a significant group-by-time interaction. Unlike prior EIT studies in predominantly sepsis- or pneumonia-related ARDS [7–9,31], our cohort consisted of patients with trauma- and postoperative-associated ARDS, a population with greater heterogeneity in lung injury, including pulmonary contusion, dependent atelectasis, pleural effusion, and altered chest wall mechanics [32,33]. These differences may influence responses to ventilatory interventions and should be considered when comparing studies across ARDS populations [34,35]. In heterogeneous lung injury, the same level of PEEP may result in recruitment in some regions while causing overdistension in others [36]. Because this trial evaluated a bundled EIT-guided ventilation strategy, without systematic collection of quantitative EIT-derived indices or EIT assessment in the control group, it cannot determine the mechanisms underlying the observed improvement in oxygenation or the relative contribution of individual intervention components.

Cstat improved over time in both groups with a nonsignificant trend favoring the EIT group. This likely reflects population-specific factors. Prior studies of EIT-based PEEP titration were largely conducted in sepsis- or pneumonia-related ARDS, where chest wall mechanics are relatively preserved and improvements in Cstat have been reported [8,9,31]. In trauma- and postoperative-associated ARDS, Cstat may be strongly influenced by chest wall factors, including injury, pain, surgical dressings, pleural effusions, and abdominal hypertension [15,34]. These factors may limit the ability of global compliance measures to reflect changes in alveolar recruitment. Accordingly, the absence of a significant between-group difference in overall Cstat is biologically plausible. Under the study conditions, intrinsic PEEP was likely minimal; therefore, total PEEP was similar to the applied PEEP, supporting the validity of Cstat and Pdriv measurements. Notably, subgroup analysis showed greater ΔCstat at Days 2–3 with the EIT-guided individualized ventilation strategy in patients without pulmonary contusion. In contrast, no difference was observed in those with contusion, consistent with evidence that pulmonary contusion represents structural lung injury with limited recruitability [16]. These findings suggest that global Cstat may be an insensitive marker in unselected trauma–postoperative ARDS. These subgroup findings should be considered exploratory and should not be interpreted as definitive evidence of treatment-effect heterogeneity. We did not measure esophageal pressure and therefore could not separate lung from chest wall mechanics; however, this limitation reflects real-world practice and underscores the potential value of bedside regional ventilation monitoring when global compliance is difficult to interpret.

MP/PBW decreased over time in both groups, with no group-by-time interaction, indicating similar temporal trends. Overall MP/PBW was modestly higher in the EIT group. In the MP framework proposed by Gattinoni et al., MP integrates the ventilator-related load generated by VT, RR, inspiratory flow, Pdriv, and PEEP [5]. In our study, higher MP/PBW paralleled greater PEEP exposure within the EIT-guided individualized ventilation strategy, while Pplat and Pdriv were not increased. This pattern supports a PEEP-related contribution to calculated MP/PBW rather than worsening respiratory mechanics or greater intrinsic lung injury. In an analysis of patients with ARDS, Costa et al. showed that MP was associated with outcome, but a simpler model using ΔP and RR captured comparable prognostic information, with ΔP having a greater effect than RR [37]. The absence of increased Pplat or Pdriv is therefore important when interpreting the modestly higher MP/PBW in our study. In an EIT-based study during decremental PEEP titration, Costa et al. showed that recruitable alveolar collapse and regional hyperdistension can be estimated at the bedside and have regional distributions [7]. This supports the concept that PEEP effects depend on lung morphology and recruitability [38]. Because quantitative EIT-derived indices were not systematically collected and EIT was not performed in the control group, our findings should be interpreted as physiologic and hypothesis-generating, not as evidence of increased injurious regional lung stress or a specific EIT-derived mechanism.

Beyond respiratory physiology, exploratory secondary analyses showed greater SOFA score reduction and shorter ICU length of stay in the EIT group; however, these findings should be interpreted cautiously because the study was not powered for clinical endpoints. No increase in ventilation-related complications was observed, with no cases of barotrauma, corticosteroid use, or ECMO in either group. These findings are consistent with prior EIT trials. Van Trung et al. reported physiological improvement without a mortality benefit in moderate to severe ARDS [31]. At the same time, He et al. observed greater SOFA score reduction with EIT-based PEEP titration but no effect on mortality or ventilation duration [9]. Improved oxygenation and more stable ventilatory conditions may have contributed to downstream physiologic recovery, but this pathway was not directly tested. Taken together, these findings support further investigation of EIT-guided individualized ventilation strategies in trauma- and postoperative-associated ARDS with particular attention to underlying lung injury patterns and patient selection.

The characteristics of the study population warrant consideration when interpreting these findings. Trauma- and postoperative-associated ARDS is characterized by heterogeneous lung injury with potentially greater recruitability in dependent regions, whereas ARDS of predominantly medical etiology more often involves diffuse alveolar damage, extensive consolidation, and a heightened inflammatory milieu that may limit responses to recruitment-based strategies [1,39–42]. These features may partly explain the oxygenation improvement observed with the EIT-guided individualized ventilation strategy and differences from prior studies in more heterogeneous or predominantly medical ARDS cohorts, although the underlying mechanism could not be determined [41,43]. The predominance of male patients reflects the epidemiology of trauma-related ARDS and is consistent with prior trauma ICU cohorts. Pulmonary contusion represents a distinct phenotype of traumatic lung injury, characterized by focal alveolar hemorrhage, interstitial edema, and regional loss of aeration adjacent to relatively preserved lung units, resulting in marked anatomic and functional heterogeneity [17,44,45]. In this context, bedside regional ventilation monitoring may help guide individualized PEEP selection, although this remains hypothesis-generating. Regional heterogeneity may result in coexistence of recruitable and non-recruitable lung units [6,7]. Although quantitative indices of collapse and overdistension were not analyzed, PEEP titration within the intervention strategy was guided by real-time EIT assessment of regional ventilation. Although exploratory and underpowered, our findings suggested possible differences in selected physiologic responses according to pulmonary contusion status; however, formal interaction testing did not identify consistent subgroup-specific temporal effects [40,43].

### Strengths

This study has several notable strengths. To our knowledge, this is among the first randomized controlled trials to evaluate an EIT-guided individualized ventilation strategy specifically in trauma- and postoperative-associated ARDS within a surgical intensive care population. Unlike prior studies that applied a fixed PEEP level or a single titration maneuver, PEEP in the intervention group was dynamically individualized according to evolving regional ventilation patterns over consecutive study days, reflecting real-world clinical decision-making. The use of prespecified physiologic endpoints, longitudinal analyses, and a standardized lung-protective ventilation strategy further strengthens the internal validity of the findings.

### Limitations

Several limitations should be acknowledged. First, this was a single-center study with a modest sample size, which may limit generalizability and statistical power for clinical outcomes. Second, potential misclassification of ARDS at screening may have introduced selection bias. Third, blinding the treating clinicians was not feasible and may have led to performance bias. Fourth, the relatively short duration of protocolized intervention and follow-up precludes assessment of longer-term effects. Fifth, the study population was enriched for trauma- and postoperative-associated ARDS, and subgroup analyses were exploratory and underpowered. Sixth, the absence of inflammatory biomarkers and detailed hemodynamic measurements limits mechanistic interpretation. Seventh, because four randomized patients were transferred early and had no analyzable post-randomization follow-up data, the analysis was not a full intention-to-treat analysis. Although the proportion of excluded patients was small (4/86), this may introduce limited attrition bias and should be considered when interpreting the randomized comparison. Missing physiologic data after early transfer, death, or clinical deterioration may not have been random; therefore, longitudinal comparisons during Days 0–3 should be interpreted cautiously. No post-event imputation was performed. Finally, mechanistic interpretation was limited because EIT was not performed in the control group and quantitative EIT-derived indices were not systematically collected. Therefore, the observed oxygenation benefit should be interpreted as the effect of the multicomponent EIT-guided individualized ventilation strategy rather than as evidence of a specific EIT-derived mechanism.

### Clinical implications and future directions

Our findings support the feasibility of an EIT-guided individualized ventilation strategy in trauma- and postoperative-associated ARDS. However, the study was not designed to determine the independent effect of EIT monitoring, nor was it powered to assess organ dysfunction, duration of ventilation, ICU length of stay, or mortality. In centers with access to EIT, this strategy may provide a practical framework for individualized PEEP selection beyond conventional PEEP/FiO₂ tables, but its clinical impact requires confirmation in larger multicenter trials.

Future studies should evaluate EIT-guided individualized ventilation strategies in larger multicenter trials powered for patient-centered outcomes. Integration of EIT with other physiologic monitoring tools, such as esophageal pressure or lung ultrasound, may further refine individualized ventilation strategies. Additionally, stratification by ARDS subphenotype and etiology should be incorporated into trial design to identify populations most likely to benefit from EIT-guided individualized ventilation approaches.

## Conclusions

In moderate-to-severe trauma- and postoperative-associated ARDS, an EIT-guided individualized ventilation strategy was associated with improved oxygenation compared with a lower-PEEP/FiO₂ strategy while preserving lung-protective ventilatory parameters. These findings suggest the physiological feasibility of this strategy, but do not establish the independent effect of EIT monitoring or the mechanistic pathway underlying the observed oxygenation benefit. Larger multicenter studies incorporating quantitative EIT-derived measurements and parallel regional ventilation monitoring in both groups are needed to determine effects on patient-centered outcomes.

## Supporting information

S1 Fig Determination of PEEP using EIT-based analysis. The recommended PEEP was identified by the manufacturer’s EIT algorithm at the intersection of the cumulative collapse (blue) and overdistension (white) curves, representing the point at which the estimated proportions of atelectasis and overinflation were balanced. In this example, the recommended PEEP was 7.1 cmH₂O.
(TIF)

S1 Table Lower PEEP/FiO₂ table used in the control group, adapted from the ARDS Network ALVEOLI trial. (DOCX)

S2 Table Estimated marginal mean PEEP (cmH₂O) over time by pulmonary contusion status and study group. (Values are estimated marginal means (95% confidence intervals) derived from the linear mixed-effects model. P values are Sidak-adjusted for between-group comparisons at each time point within each subgroup).
(DOCX)

S3 Table Fixed effects from exploratory subgroup linear mixed-effects models by pulmonary contusion status. (Values are F statistics with denominator degrees of freedom and two-sided p values from linear mixed-effects models with fixed effects for treatment group, time, pulmonary contusion status, and interaction terms, with subject-specific random intercepts and autoregressive covariance structure).
(DOCX)

S4 Table Sidak-adjusted pairwise comparisons for exploratory subgroup analyses. (Values are from Sidak-adjusted pairwise comparisons).
(DOCX)

S5 File CONSORT 2025 checklist. (DOCX)

S6 File PLOS Human Participants Research Checklist 2025. (PDF)

S7 File Research protocol. (DOCX)
