# Supplementary material for: Electrical impedance tomography–guided individualized ventilation strategy in patients with trauma-related and postoperative acute respiratory distress syndrome
Source: PLoS One. 2026 Aug 24;21(8):e0355609. doi: 10.1371/journal.pone.0355609 (PMC13502546; doi:10.1371/journal.pone.0355609)
Supplement: Supplementary file 2 [file pone.0355609.s009.pdf]

Check for updates

ORIGINAL ARTICLE

Ventilatory Variables and Mechanical Power in Patients with Acute Respiratory Distress Syndrome

Eduardo L. V. Costa[1,2], Arthur S. Slutsky[3,4], Laurent J. Brochard[3,4*], Roy Brower[5], Ary Serpa-Neto[6], Alexandre B. Cavalcanti[7], Alain Mercat[8], Maureen Meade[9], Caio C. A. Morais[1], Ewan Goligher[3,10,11], Carlos R. R. Carvalho[1], and Marcelo B. P. Amato[1]

[1]Laboratório de Pneumologia, Laboratório de Investigação Médica 09, Disciplina de Pneumologia, Instituto do Coração, Hospital das Clínicas, Faculdade de Medicina, Universidade de São Paulo, São Paulo, São Paulo, Brazil; [2]Instituto de Ensino e Pesquisa, Hospital Sírio-Libanes, São Paulo, São Paulo, Brazil; [3]Interdepartmental Division of Critical Care Medicine, University of Toronto, Toronto, Ontario, Canada; [4]Keenan Research Centre, Li Ka Shing Knowledge Institute, St. Michael's Hospital, Toronto, Ontario, Canada; [5]Division of Pulmonary and Critical Care Medicine, School of Medicine, Johns Hopkins University, Baltimore, Maryland; [6]Hospital Israelita Albert Einstein, São Paulo, São Paulo, Brazil; [7]Instituto de Pesquisas Hospital do Coração–Hospital do Coração, São Paulo, São Paulo, Brazil; [8]Département de Médecine Intensive–Réanimation, Centre Hospitalier Universitaire d'Angers, Université d'Angers, Angers, France; [9]Department of Medicine, McMaster University, Hamilton, Ontario, Canada; [10]Division of Respirology, Department of Medicine, University Health Network and Sinai Health System, Toronto, Ontario, Canada; and [11]Toronto General Hospital Research Institute, Toronto General Hospital, Toronto, Ontario, Canada

ORCID IDs: 0000-0002-6941-3626 (E.L.V.C.); 0000-0002-0990-6701 (E.G.).

Abstract

Rationale: Mortality in acute respiratory distress syndrome (ARDS) has decreased after the adoption of lung-protective strategies. Lower V_T, lower driving pressure (ΔP), lower respiratory rates (RR), and higher end-expiratory pressure have all been suggested as key components of lung protection strategies. A unifying theoretical explanation has been proposed that attributes lung injury to the energy transfer rate (mechanical power) from the ventilator to the patient, calculated from a combination of several ventilator variables.

Objectives: To assess the impact of mechanical power on mortality in patients with ARDS as compared with that of primary ventilator variables such as the ΔP, V_T, and RR.

Methods: We obtained data on ventilatory variables and mechanical power from a pooled database of patients with ARDS who had participated in six randomized clinical trials of protective mechanical ventilation and one large observational cohort of patients with ARDS. The primary outcome was mortality at 28 days or 60 days.

Measurements and Main Results: We included 4,549 patients (38% women; mean age, 55 ± 23 yr). The average mechanical power was $0.32 \pm 0.14\ J \cdot min^{-1} \cdot kg^{-1}$ of predicted body weight, the ΔP was 15.0 ± 5.8 cm H_2O, and the RR was 25.7 ± 7.4 breaths/min. The driving pressure, RR, and mechanical power were significant predictors of mortality in adjusted analyses. The impact of the ΔP on mortality was four times as large as that of the RR.

Conclusions: Mechanical power was associated with mortality during controlled mechanical ventilation in ARDS, but a simpler model using only the ΔP and RR was equivalent.

Keywords: respiratory distress syndrome; adult; respiration; artificial; ventilator-induced lung injury

(Received in original form September 9, 2020; accepted in final form March 31, 2021)

*L.J.B. is Deputy Editor of *AJRCCM*. His participation complies with American Thoracic Society requirements for recusal from review and decisions for authored works.

Supported in part by the Fundação de Amparo e Pesquisa do Estado de São Paulo and the Canadian Institutes of Health Research.

Author Contributions: Study concept and design: E.L.V.C., A.S.S., and M.B.P.A. Acquisition of data: A.S.S., L.J.B., R.B., A.S.-N., A.B.C., A.M., M.M., C.R.R.C., and M.B.P.A. Analysis and interpretation of data: E.L.V.C., A.S.S., and L.J.B. Drafting of the manuscript: E.L.V.C., A.S.S., and M.B.P.A. Critical revision and important intellectual contribution: A.S.S., L.J.B., R.B., A.S.-N., A.B.C., A.M., M.M., C.C.A.M., and C.R.R.C. Statistical analysis: E.L.V.C., A.S., E.G., and M.B.P.A. Administrative support: C.R.R.C. and M.B.P.A. Study supervision: M.B.P.A.

Correspondence and requests for reprints should be addressed to Eduardo L. V. Costa, M.D., Faculdade de Medicina da Universidade de São Paulo, 455 Dr Arnaldo Avenue, Room 2144, São Paulo, SP, Brazil. E-mail: eduardoleitecosta@gmail.com.

This article has a related editorial.

This article has an online supplement, which is accessible from this issue's table of contents at www.atsjournals.org.

Am J Respir Crit Care Med Vol 204, Iss 3, pp 303–311, Aug 1, 2021
Copyright © 2021 by the American Thoracic Society
Originally Published in Press as DOI: 10.1164/rccm.202009-3467OC on March 31, 2021
Internet address: www.atsjournals.org

At a Glance Commentary

Scientific Knowledge on the Subject: Lung-protective strategies for acute respiratory distress syndrome (ARDS) are based on lower V_T, lower driving pressure (ΔP), limited plateau pressure, limited respiratory rates (RRs), and enough end-expiratory pressure. Mechanical power has been promulgated as a unifying theoretical explanation that lumps all of these variables into one measure of energy transferred from the ventilator to the respiratory system. In this theoretical construct, this transferred energy would be the cause of ventilator-induced lung injury. It remains unclear which ventilatory settings composing the mechanical power delivered from a mechanical ventilator to a patient with ARDS are associated with mortality or how the predictive values of these settings compare with those of mechanical power.

What This Study Adds to the Field: On the basis of an analysis of 4,549 patients from a pooled database of patients with ARDS, we found that among ventilator variables, only the ΔP and RR had significant associations with mortality. These two variables combined according to the formula $[(4 \times \Delta P) + RR]$ were at least as informative as mechanical power. Ventilatory strategies with lower V_T and higher RRs may be beneficial for patients with low respiratory system compliance.

Lung-protective strategies in patients with acute respiratory distress syndrome (ARDS) have demonstrated survival benefits in some randomized clinical trials (1–3). Lower V_T (1–3), lower driving pressure (ΔP) (4, 5), lower plateau pressure (Pplat) (6, 7), and higher positive end-expiratory pressure (PEEP) (8) have all been suggested to decrease mechanical stresses imposed on inflamed and fragile lung tissues and hence act as components of an effective lung protection strategy.

One potentially important variable that has not been directly studied in randomized trials in humans is the respiratory rate (RR). Randomized trials with the largest between-arm differences in RRs have aimed at high versus low V_T, and the differences in outcomes because of the V_T itself might have offset the importance of the RR. Studies in experimental models suggest that an increased RR worsens ventilator-induced lung injury (VILI) (9, 10), but the relative contribution of the RR versus the other factors is not known. This is an important issue because clinicians often face the dilemma of optimizing the ventilatory strategy when one component negatively affects another component of lung protection. For example, decreasing ΔP may require higher RRs to maintain adequate CO_2 removal, with unknown net consequences on clinical outcomes.

A unifying theoretical explanation (11, 12) has recently been proposed that could address these trade-offs. On the basis of basic thermodynamic principles, this hypothesis attributes lung injury to the energy transfer rate (mechanical power) from the ventilator to the patient. This energy dissipation within the lungs can lead to heat production, inflammation, and disruptive deformation of cells and the extracellular matrix (12). Mathematically, the mechanical power is the integral of the area under airway pressure versus the V_T plots multiplied by the RR and is given by the following equation: Power (J/min) $= 0.098 \times V_T \times RR \times [PEEP + 0.5 \times P + (Ppeak - Pplat)]$, where Ppeak represents peak pressure.

This model is appealing because it includes all the ventilator variables that have been implicated in VILI. However, it lacks clinical validation. Furthermore, mechanical power is composed of resistive, static, and dynamic (elastic) forces generating tissue strain, and it is unclear whether each of these forces contributes to lung injury during mechanical ventilation. In addition, the power computation comprises multiple components, which makes it difficult for the clinician to establish priorities among different ventilator settings. If power is high, for instance, which of the five to six single ventilator parameters should be changed first?

The ΔP was defined as the difference between the pressure at the airway opening after a brief end-inspiratory pause (Pplat) and the PEEP level, which is easily captured (in patients under controlled ventilation) at the bedside. Recently, ΔP has been shown to be the ventilator variable with the strongest predictive performance for both lung injury and mortality, exhibiting the highest standardized effect size among all ventilator variables (4). However, the impact of the RR was not assessed. In addition, the prognostic information carried by ΔP has never been tested against the risk prediction provided by mechanical power.

In this study, we assessed the impact on mortality of mechanical power and the specific components of mechanical power (i.e., static, dynamic, and resistive components) and compared these to the impact of simpler primary ventilator variables in patients with ARDS.

Methods

Patients

We used patient-level data of 4,549 patients, obtained from a large observational cohort (13) and from six randomized clinical trials on protective mechanical ventilation (2, 14–18) (*see* Table E1 in the online supplement). The observational cohort comprised 693 patients with ARDS from the Medical Information Mart for Intensive Care III (MIMIC-III) (version 1.4) database, a freely accessible database (13). Two trials (2, 14) ($N = 880$) examined lower V_T versus higher V_T. Four trials (15–18) ($N = 2,976$) used low V_T and examined higher PEEP values versus lower PEEP values. Patients were randomized in a 1:1 ratio to either low V_T versus high V_T (2, 14) or low PEEP versus high PEEP (15–18) if they met the criteria for hypoxemic respiratory failure for 36–72 hours as per the original trials' protocols.

We included only patients under controlled mechanical ventilation to assess the effect of *set* RRs on mortality because measurements of Pplat are difficult to interpret in spontaneously breathing patients. To this end, patients ventilated in pressure-support mode or whose RRs exceeded the set RR in an assist-controlled mode (suggesting ventilatory efforts) were excluded.

Direct Data

For every patient, we obtained the age, sex, and adjusted risk of death on the basis of the Acute Physiology and Chronic Health Evaluation, Simplified Acute Physiology Score 2 score, or Simplified Acute Physiology Score 3 score. Values of arterial pH, $Pa_{O_2}/F_{I_{O_2}}$, Pa_{CO_2}, respiratory system compliance, PEEP, V_T (scaled to the predicted body weight [PBW]), ΔP, Pplat, and RR were based on average values for the first 24 hours after

randomization or for the first 24 hours of mechanical ventilation in the MIMIC-III cohort.

Derived Data: Combined Ventilator Variables

Mechanical power was obtained as the algebraic sum of three components (Figure E1):

1. Elastic–static power related to PEEP: elastic–static power (J/min) = $0.098 \times V_T \times RR \times PEEP$.
2. Elastic–dynamic power, related to ΔP: elastic–dynamic power (J/min) = $0.098 \times V_T \times RR \times 0.5 \times \Delta P$.
3. Resistive power related to resistance in the ventilator circuit, endotracheal tube, and airways: resistive power (J/min) = $0.098 \times V_T \times RR \times (Ppeak - Pplat)$.

Data on Ppeak were available only in the MIMIC-III cohort. In the remaining studies, we computed the mechanical power using a simplified version of the formula, ignoring the resistive term.

Outcomes

The primary outcome was a 60-day mortality except in the MIMIC-III cohort and in ART (Alveolar Recruitment for ARDS Trial) (18), from which only 28-day mortality was available.

Statistical Analysis

Continuous data are presented as means and SDs or medians and interquartile ranges. Categorical data are presented as counts and percentages. We used multivariable logistic regression to assess the association between mechanical power and single ventilator variables with mortality. To adjust for baseline disease severity, we created a *baseline risk model* based on potential confounders identified in a directed acyclic graph (Figure E2). The set of confounders included the Pa_{O_2}/FI_{O_2} ratio, Pa_{CO_2}, arterial pH, respiratory system compliance, and ventilatory ratio as continuous variables and two categorical dummy variables: one representing study trial and another representing study arm (Table E2). All subsequent analyses were preadjusted for this baseline risk model. In each of the models, models A through E, all variables were included simultaneously.

In addition, we divided the whole population into quintiles of ΔP, V_T, RR, or power. We assessed the association between belonging to a given quintile and mortality in a model adjusted to the baseline risk model.

We also conducted a multilevel mediation analysis to assess to what extent the ΔP and RR (indirect effects) mediated the effect of randomization on mortality. A multivariable linear regression model was used to calculate the coefficients expressing the influence of *randomization* on the two tested mediators (ΔP and RR). We then calculated the adjusted influence of the potential mediators on mortality. We did not test interaction terms. The net effect sizes of the two potential mediators on mortality were multiplied by the mean effect of randomization on potential mediators. Confidence intervals were obtained by using Preacher and Hayes bootstrap method (19). The MIMIC-III cohort was not included in the mediation analysis.

The analyses were conducted using R version 3.6.2 (R Foundation for Statistical Computing). *P* values <0.05 were considered to indicate significance.

Isocapnic Curves

To illustrate the trade-off between the risks associated with changes in the RR and ΔP, we computed the risk of death at each combination of the RR and ΔP required to maintain the same Pa_{CO_2} (isocapnic curves). The theoretical curves represent a situation in which one attempts to reduce the V_T (and consequently the ΔP) at the expense of increasing the RR to maintain the alveolar ventilation at a constant value (additional details in the online supplement). Specifically, alveolar ventilation was kept at the average value of our sample for varying values of the RR from 15 to 45 breaths/min, a range that encompasses approximately 90% of the RRs in our data set. For simplicity, we assumed an airway dead space of 1.5 ml/kg of PBW and an alveolar dead space of 20% of the V_T. Subsequently, we performed a sensitivity analysis examining the effects of larger airway dead spaces (up to 3.0 ml/kg of PBW) and larger alveolar dead spaces (up to 50% of V_T). The ΔP resulting from each computed V_T value was calculated by assuming that the respiratory system compliance did not vary with the RR and that there was no intrinsic PEEP. Isocapnic curves were computed at varying levels of respiratory system compliance, ranging from 0.14 to 0.86 ml/cm H_2O/kg of PBW (corresponding to compliances of 10 and 60 ml/cm H_2O for patients with a PBW of 70 kg). The risk of death was computed from the ΔP and RR obtained at each point along each curve using the effect sizes of the ΔP and RR obtained in the multivariable model of mortality.

Results

The analysis included 4,549 patients (mean age, 56 ± 24 yr), of whom 38% were women. On average, the mechanical power was 0.32 ± 0.14 $J \cdot min^{-1} \cdot kg^{-1}$ of PBW, the ΔP was 15.0 ± 5.8 cm H_2O, and the RR was 25.7 ± 7.4 breaths/min. The overall mortality was 38%. Additional details are shown in the online supplement (Table E3 and Figure E3).

Univariate predictors of 28-day or 60-day mortality were the ΔP, PEEP, Pplat, RR, and mechanical power. To assess independent associations between ventilator variables and mortality, we preadjusted all subsequent models to the baseline risk model, which includes validated predictors for ARDS mortality: age, risk of death, Pa_{O_2}/FI_{O_2} ratio, Pa_{CO_2}, arterial pH, respiratory system compliance, ventilatory ratio, and two categorical dummy variables (study trial and study arm).

Single Ventilator Variables

When all single ventilator variables were included in a multivariable model, only the ΔP and RR remained significantly associated with mortality (Figure 1, model A). We did not find a significant interaction between the ΔP and RR ($P = 0.08$). The effect size of each 1–cm H_2O increase in the ΔP was approximately 4.0 times that of each 1–breath/min increase in RR (Figure E4; this ratio varied from 3.6 to 4.0 in patients with a V_T less than 12 ml/kg of PBW). On the basis of this finding, we created a variable that was computed as a linear combination of the ventilator variables $[(4 \times \Delta P) + RR]$.

Combined Ventilator Variables

In alternative models, we substituted components of mechanical power (Figure 1, model C) or total mechanical power (Figure 1, model B) for the individual ventilator variables tested in model A. In this model, mechanical power, specifically its elastic (dynamic) component, was associated with mortality. Of note, the association with mortality was significantly stronger for the dynamic component of power than for total power ($P < 0.001$).

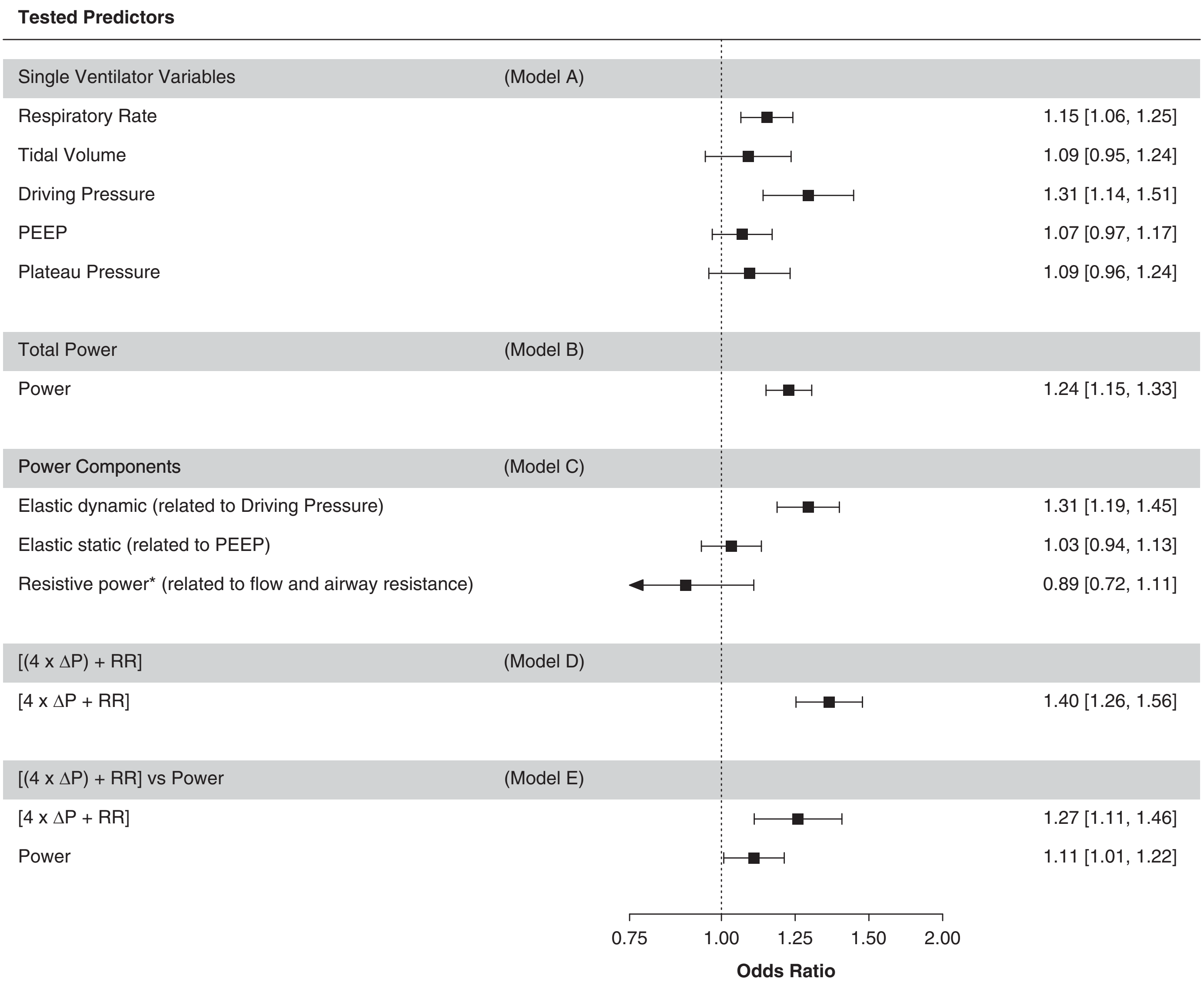

Figure 1. Forest plot of odds ratios for death relative to the population average after adjustment for the seven covariates (trial, study arm, respiratory system compliance, ventilatory ratio, arterial pH, Pa_{CO_2}, and Pa_{O_2}/F_{IO_2}) in the base model. All variables were standardized, and the odds ratios correspond to a 1-SD increment in the respective variable, using bedside units of measurement (i.e., breaths/min, ml/kg of ideal body weight, cm H_2O, and so forth). In model A, we tested whether single ventilator variables (respiratory rate [RR], V_T, driving pressure [ΔP], positive end-expiratory pressure [PEEP], and plateau pressure) were significantly associated with mortality. Note that the ΔP and RR were the only single ventilator variables significantly associated with mortality. In model B, we assessed whether power alone (total power, after forced inclusion in a model with the seven baseline covariates) was associated with mortality. Total power was associated with mortality. In model C, we tested whether the three additive power components (i.e., elastic–dynamic, elastic–static, and resistive components) were associated with mortality after a forced inclusion in the multivariable model. Only the elastic–dynamic component was significantly associated with mortality. In model D, we tested whether the linear combination of the ΔP and RR (i.e., [(4 × ΔP) + RR], weighted according to their relative effect sizes in model A; *see* METHODS) was significantly associated with mortality. In model E, we built a multivariable model by combining models B and D to assess the independent association of each variable with mortality risk. The linear combination of the ΔP and RR ([(4 × ΔP) + RR]) had a stronger association with mortality than total power. The squares represent the adjusted odds ratios of dying for each 1-SD increment of a given variable, and the error bars represent 95% confidence intervals. SDs were 7.4 breaths/min for the RR, 2.0 ml/kg for the V_T, 5.8 cm H_2O for the ΔP, 4.8 cm H_2O for the PEEP, 6.5 cm H_2O for the plateau pressure, 0.07 $J \cdot min^{-1} \cdot kg^{-1}$ for elastic–dynamic power, 0.10 $J \cdot min^{-1} \cdot kg^{-1}$ for elastic–static power, 0.05 $J \cdot min^{-1} \cdot kg^{-1}$ for resistive power, and 0.14 $J \cdot min^{-1} \cdot kg^{-1}$ for total power. *The resistive component could only be computed in the 693 patients in the Medical Information Mart for Intensive Care III cohort.

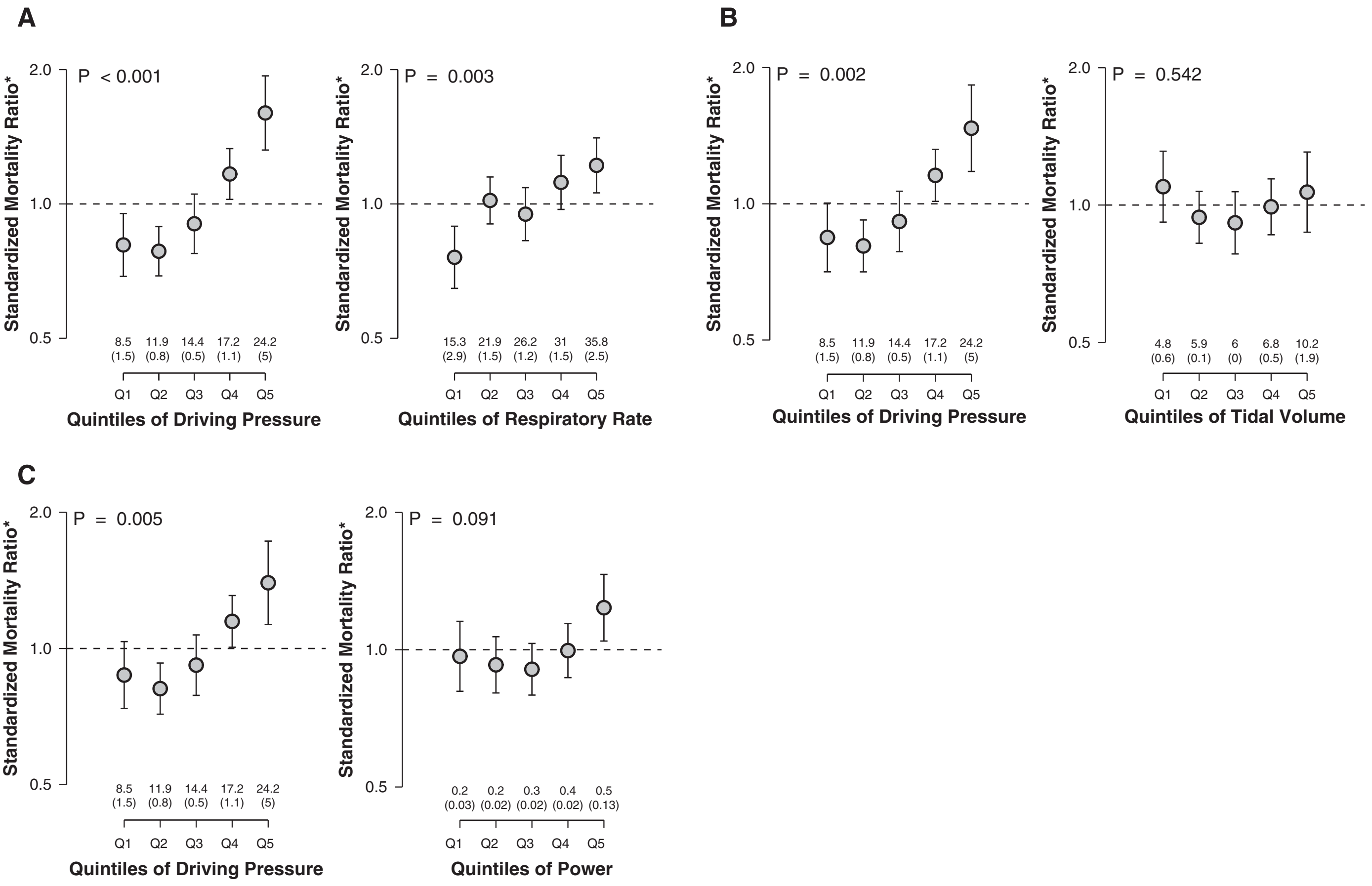

Figure 2. We partitioned our data set into five (roughly) equally sized subsamples (quintiles), each including approximately 935 patients, and calculated the adjusted mortality for each subsample compared with the average mortality. The circles represent the odds ratio for mortality calculated for each quintile after multivariable adjustment for the seven covariates (trial, study arm, respiratory system compliance, ventilatory ratio, arterial pH, Pa_{CO_2}, and $Pa_{O_2}/F_{I_{O_2}}$) in the base model as well as for the ventilator variable not involved in a given subsampling in each pairwise comparison. For example, in *A*, the mortality for each quintile of the respiratory rate (RR) was adjusted for the base model and driving pressure (ΔP), and the mortality of each quintile of the ΔP was adjusted for the base model and RR. Note that in every pairwise comparison, subsamples of ΔP remained significant. Also significant was the subsampling of the RR after an adjustment for (*A*) ΔP. Conversely, subsampling of both (*B*) V_T and (*C*) power did not carry additional significant information after adjustment for ΔP. Note that risks of death associated with ΔP changed somewhat in *A*–*C* because the number of patients was different because of missing data (Table E5) and because the mortality in each of these figures is adjusted for the baseline risk model, ΔP, and one of the following: RR (*A*), V_T (*B*), or power (*C*). The error bars represent 95% confidence intervals. The resistive component of total power could only be computed in the 693 patients in the Medical Information Mart for Intensive Care III cohort. *Adjusted for the base model. Q = quintile.

Combined versus Single Ventilator Variables

The combined ventilator variable [(4 × ΔP) + RR] was significantly associated with mortality (model D). After including mechanical power and [(4 × ΔP) + RR] in the same multivariable model, the total mechanical power was no longer associated with mortality (Figure 1, model E), implying that there was no independent information carried by this variable. In contrast, [(4 × ΔP) + RR] remained a significant predictor within the model, demonstrating that a model with this variable outperformed the predictions afforded by power.

Analysis by Quintiles of Single Ventilator Variables and Power

We partitioned our data set into five equally sized subsamples (quintiles), each including approximately 935 patients, according to increasing levels of clinically relevant variables (ΔP, RR, and V_T) and according to total power. The adjusted mortality for each subsample was greater than the average, with increasing values for the ΔP and RR but not for the V_T or total power (Figure 2). This analysis is consistent with a better discriminatory performance of [(4 × ΔP) + RR] as compared with total power (Figure E5).

Isocapnic Curves

On the basis of an average effect size of the ΔP being approximately four times that of the RR (Figure 3A), we computed the predicted mortality under simulated isocapnic conditions (Figure 3B and Figures E6–E8). After computing the change in V_T required to maintain constant alveolar ventilation with changes in the RR, we found a U-shaped relationship between mortality and the V_T; the same was true for the relationship between mortality and the RR. The simulation (Figure 3B) represents two situations in which reducing the V_T (and consequently the ΔP) forces an increase in the RR to maintain

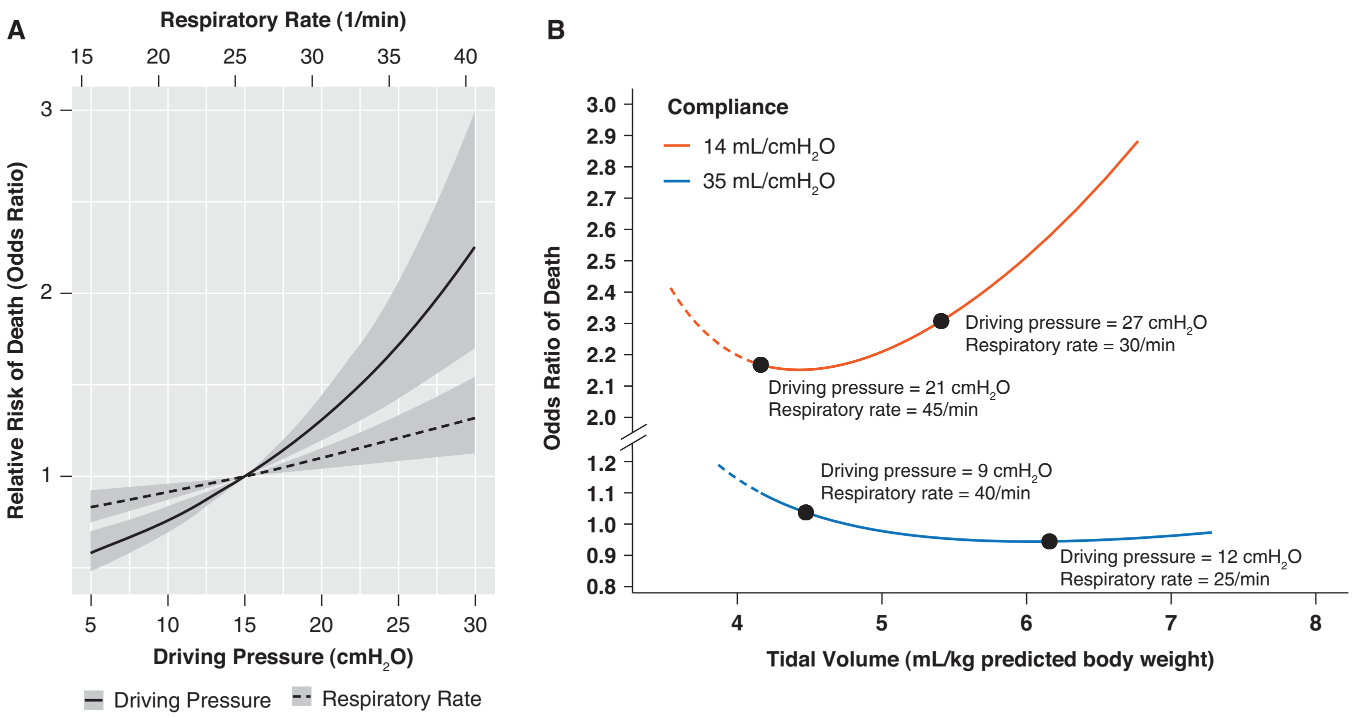

Figure 3. (*A*) Modeled odds ratio for death according to either driving pressure (ΔP) or respiratory rate (RR) after multivariable adjustment for the seven covariates (trial, study arm, respiratory system compliance, ventilatory ratio, arterial pH, Pa_{CO_2}, and Pa_{O_2}/FI_{O_2}) in the base model as well as for the single ventilator variables. The abscissa represents either the ΔP (lower axis) or the RR (upper axis). These values were multiplied by their respective effect sizes to obtain the odds ratio for death. The shaded areas represent 95% confidence intervals. (*B*) Simulated curves illustrating the trade-off between the risks associated with changes in ΔP versus changes in the RR under isocapnic conditions. The simulation represents a situation in which one attempts to reduce V_T (and consequently ΔP) at the expense of an increased RR (moving to the left along the *x*-axis), keeping alveolar ventilation constant (*see* online supplement). Each curve represents the odds ratio for mortality relative to the average of the study population plotted against the V_T. The odds ratio was calculated after multivariable adjustment for the seven covariates (trial, study arm, respiratory system compliance, ventilatory ratio, arterial pH, Pa_{CO_2}, and Pa_{O_2}/FI_{O_2}) in the base model as well as for the single ventilator variables. The different lines illustrate simulations with compliance below average (14 ml/cm H_2O) or above average (35 ml/cm H_2O), and the dashed portions are extrapolations for RRs from 45 to 60 breaths/min, which represented less than 1% of our sample. Note that for similar changes in V_T, the low-compliance condition has the steepest changes in the odds ratio for mortality. The RR that minimizes the risk of dying is 48 breaths/min in the low-compliance scenario and 30 breaths/min in the higher-compliance scenario. In the upper curve (lower compliance), the rightward circle shows an RR of 30 breaths/min, which corresponds to a ΔP of 27 cm H_2O. Under isocapnic conditions, an increase in the RR to 45 breaths/min (leftward circle) will be associated with a decrease in ΔP to 21 cm H_2O and a lower odds ratio for death. Conversely, with a higher compliance, the same 15-breaths/min increase in RR will correspond to a mild reduction in ΔP of 3 cm H_2O, an unfavorable trade-off as reflected in the higher odds ratio for death.

constant alveolar ventilation. The trade-off between a lower ΔP and a higher RR is beneficial at a lower compliance but not at a higher compliance.

For the sickest patients (i.e., low compliance and high alveolar dead space), the lowest predicted mortality occurs at higher RRs, which are often >35 breaths/min. Interestingly, in conditions with high compliance, the optimal V_T can be as high as 9 ml/kg of PBW (Figures E6A, E7A, and E8A).

Mediation Analysis

We found that both the ΔP and the RR mediated the effect of randomization (Figure 4). Randomization to the intervention arm caused a significant decrease in the ΔP, and this decrease explained away the direct effect of randomization (cooperative mediator). Conversely, randomization provoked an increase in the RR, which would predict an increase in mortality in patients randomized to the intervention arm (competitive mediation). The net effect of randomization was positive, despite the increase in the RR, because of the pronounced reduction in the ΔP.

Discussion

Our main findings are that *1)* among the ventilator variables used in the computation of mechanical power, only the ΔP and RR were independently associated with survival, and the effect of ΔP was not modified by the RR; *2)* these two variables together (i.e., ΔP and RR), readily available at the bedside, were comparable with mechanical power in terms of predicting mortality; *3)* mechanical power was independently associated with mortality, but this was mainly because of its dynamic–elastic component; and *4)* the greater impact of the ΔP on mortality (when compared with the RR) may have practical implications: ventilatory strategies with higher RRs, associated with a lower V_T, can be beneficial for very sick patients in a trade-off that can be estimated. We present a model for such estimation.

Our finding of an association between mechanical power and survival was not

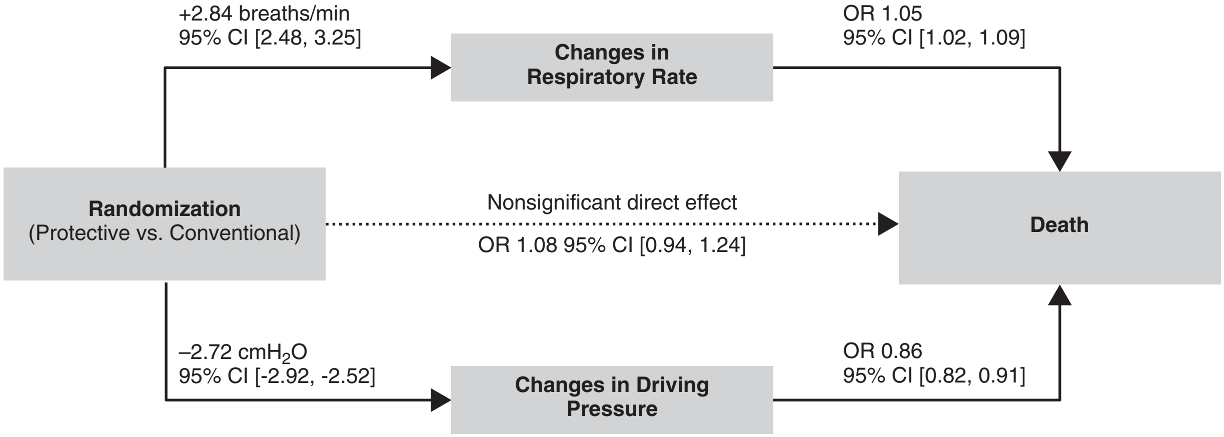

Figure 4. Mediational model showing the contribution of driving pressure (ΔP) and respiratory rate (RR) to mortality. The solid arrows in the path diagram represent the significant association between variables, with the left-to-right direction representing an independent-to-dependent relationship. The dotted arrows represent nonsignificant effects. A multivariable linear regression model was used to calculate the coefficients expressing the influence of *randomization* on the two tested mediators (ΔP and RR). The coefficients are shown in the middle of the arrows linking randomization to potential mediators, indicating that randomization caused a significant change of −2.73 cm H_2O in ΔP and of +3.18 breaths/min in the RR. We then calculated the adjusted influence of the potential mediators on mortality, after accounting for baseline risk factors. The net log ORs of the two potential mediators on mortality (shown in the middle of the arrows linking mediators to death) are multiplied by the mean influence of randomization on potential mediators. Note that ΔP explains the protective effect of randomization (cooperative mediation). Conversely, the effect of randomization on RR would predict an increase in mortality in patients randomized to the intervention arm (competitive mediation). In other words, despite the increase in RR, randomization to the intervention arm was still protective because of the pronounced reduction in ΔP. CI = confidence interval; OR = odds ratio.

unexpected. Power increases with increments in PEEP, ΔP, VT, RR, and Ppeak (11). Some of these variables are linked to disease severity. For example, more severe hypoxemia can lead clinicians to increase the PEEP (e.g., using PEEP/$F_{I_{O_2}}$ tables [2, 15, 17]). Consequently, patients in a more severe condition receive a higher PEEP, such that PEEP may be a marker of severity and may not necessarily be harmful. Conversely, other components, like the VT and ΔP, are implicated in promoting VILI (2, 4). Power is thus a composite of risk factors linked to underlying disease severity and modifiable factors that cause lung injury, such as the VT and RR (mediators). High power values could therefore have different interpretations: *1*) severe underlying disease and adequate ventilator settings, *2*) severe underlying disease and injurious ventilatory settings, or simply *3*) injurious ventilator settings. Recently, Zhang and colleagues (20) found that the predictive discrimination of mechanical power could not be improved by using machine learning to find the best combination of ventilator variables. Their analysis was not fully adjusted for the baseline risk of dying, and their findings support the concept that power carries a strong component of disease severity. Therefore, although potentially useful as a prognostic marker, power—as a lumped parameter—is not particularly helpful in establishing priorities among different ventilator settings.

Among the three components of power (Figure E1), only the cyclic, elastic–dynamic component was significantly associated with mortality after adjusting for baseline severity. The addition of the other two components (elastic–static and resistive) degraded the association of power with mortality, likely because of the introduction of noise. These findings are compatible with the hypothesis that cyclic loading of the lungs because of oscillating mechanical stresses is more injurious than static loading (21) and are also in line with our finding that the only two primary variables significantly associated with mortality were the ΔP and RR.

Our findings suggest that the higher RRs could be injurious *per se*, although this requires confirmation in a clinical trial. This study analyzed RRs under controlled mechanical ventilation, and rates were likely changed as providers responded to the pH or Pa_{CO_2} rather than responding to another existing condition. Consistent with this hypothesis, clinical conditions associated with increased RRs in the univariate analysis, such as sepsis (data not shown), were no longer significant after preadjustment for the baseline risk model, which included the pH and Pa_{CO_2}. In addition, we found that changes in the RR were caused by randomization, an exogenous factor unrelated to the underlying disease. The mediational analysis demonstrated that an increase in the RR was associated with harm. Although still susceptible to residual confounding, these findings suggest that not only the degree of stress and strain per breath (captured by the ΔP) but also how often the stress/strain is repeated (captured by the RR) is important. We found that the key value in this relationship is close to four; each 1–cm H_2O increase in ΔP would need to be compensated by a decrease in the RR of four or more breaths/min to not increase mortality. In other words, changes in ventilatory settings such that $[(4 \times \Delta P) + RR]$ decreases should be beneficial. This finding can explain, for example, why in classic low-VT versus high-VT trials, the low-VT arms fared better, even with higher RRs (1, 2). Interestingly, this finding implies that an optimal RR can be calculated using a simple formula that is based on the patient energy expenditure, target arterial CO_2, and respiratory system compliance (*see* the mathematical expression and its derivation in the online supplement).

If confirmed in a randomized clinical trial, the potential implications on clinical

decision-making are that a low ΔP and a low RR should both be targets. For a given target Pa_{CO_2}, reductions of the RR require a higher VT (and consequently a higher ΔP) and *vice versa* (Figure 3B), provided that the dead space remains constant. The same change in VT will produce larger changes in the ΔP in patients with lower respiratory system compliance. Consequently, the trade-off between the ΔP and RR is influenced by compliance, which itself can be affected by other settings, such as PEEP. For example, in a patient with a respiratory system compliance of 0.2 ml/cm H_2O/kg of PBW and a dead space 1.5 ml/kg of PBW, increasing the RR from 30 to 45 breaths/min will be associated with a decrease in the ΔP from 27 to 21 cm H_2O under isocapnic conditions (Figure 3B). This will result in an advantageous change in $[(4 \times \Delta P) + RR]$ from 138 to 129, indicating that the 6–cm H_2O fall in the ΔP is beneficial despite the increase in the RR of 15 breaths/min. However, another patient with a higher compliance of 0.5 ml/cm H_2O/kg of PBW would benefit from a decrease in the RR from 40 to 25/min, even though this would be associated with an increase in the ΔP from 9 to 12 cm H_2O, because $[(4 \times \Delta P) + RR]$ would fall from 76 to 73.

Our study has some strengths. The most important is the pool of randomized studies of both low VT versus high VT and low PEEP versus high PEEP. The use of this patient population is paramount because randomization is essential to break the collinearity between the PEEP and underlying disease severity (Figure E9) and between the VT and dead space (also a marker of underlying severity [22, 23]). For example, in some low-PEEP versus high-PEEP studies, PEEP followed a PEEP–FI_{O_2} table in each study arm, but additional variability was introduced according to the group assignment, which was uncorrelated with underlying severity. In addition, our analysis fulfills most characteristics of clinical trial emulation: the studies had similar eligibility criteria, we used regression to adjust for possible confounders identified on the basis of the directed acyclic graph, and outcome ascertainment was guaranteed by our use of the primary outcome of the original trials (24).

Our study also has several limitations. First, we were only able to compute the resistive component of power in the MIMIC-III cohort. In the randomized trials, only the simplified version without the resistive term was computed. Second, we did not adjust for a patient's specific dead space. We used the ventilatory ratio as a surrogate instead. We cannot rule out that some of the associations of the RR with mortality may be secondary to a higher dead space, a variable that is known to be associated with ARDS severity (23). Third, we used the ratio of decreased RR/increased ΔP of 4 $min^{-1} \cdot cm\ H_2O^{-1}$. This threshold actually corresponds to that found in a subsample with a VT ≤12 ml/kg of PBW (Figure E4), which seemed a reasonable choice considering that VTs >12 ml/kg are now seldom used. Fourth, we excluded patients with evidence of assisted breathing to whom our findings may not apply similarly. Fifth, we did not have data on the incidence or magnitude of intrinsic PEEP, which might have underestimated the ΔP in some cases. In addition, in the presence of intrinsic PEEP, the ideal combination of the RR and ΔP might be unattainable. Sixth, we did not assess the effect of PEEP on compliance or dead space, which could affect the choice of optimal ventilatory settings. Finally, even adjusting for potential confounders on the basis of the directed acyclic graph, it is possible that there was residual confounding. Therefore, our findings do not necessarily indicate causality. As such, we cannot conclude that changing the ventilatory strategy on the basis of our findings will lead to decreased mortality; this requires validation.

In conclusion, although mechanical power was associated with mortality in patients with ARDS, the ΔP and RR were as informative and easier to assess at the beside. Whether a ventilatory strategy based on these variables improves outcomes needs to be tested in randomized controlled studies. ■

Author disclosures are available with the text of this article at www.atsjournals.org.

References

1. Amato MBP, Barbas CSV, Medeiros DM, Magaldi RB, Schettino GP, Lorenzi-Filho G, *et al.* Effect of a protective-ventilation strategy on mortality in the acute respiratory distress syndrome. *N Engl J Med* 1998;338:347–354.
2. Brower RG, Matthay MA, Morris A, Schoenfeld D, Thompson BT, Wheeler A; Acute Respiratory Distress Syndrome Network. Ventilation with lower tidal volumes as compared with traditional tidal volumes for acute lung injury and the acute respiratory distress syndrome. *N Engl J Med* 2000;342: 1301–1308.
3. Villar J, Kacmarek RM, Pérez-Méndez L, Aguirre-Jaime A. A high positive end-expiratory pressure, low tidal volume ventilatory strategy improves outcome in persistent acute respiratory distress syndrome: a randomized, controlled trial. *Crit Care Med* 2006;34:1311–1318.
4. Amato MBP, Meade MO, Slutsky AS, Brochard L, Costa ELV, Schoenfeld DA, *et al.* Driving pressure and survival in the acute respiratory distress syndrome. *N Engl J Med* 2015;372:747–755.
5. Neto AS, Hemmes SNT, Barbas CSV, Beiderlinden M, Fernandez-Bustamante A, Futier E, *et al.*; PROVE Network Investigators. Association between driving pressure and development of postoperative pulmonary complications in patients undergoing mechanical ventilation for general anaesthesia: a meta-analysis of individual patient data. *Lancet Respir Med* 2016;4:272–280.
6. Hager DN, Krishnan JA, Hayden DL, Brower RG; ARDS Clinical Trials Network. Tidal volume reduction in patients with acute lung injury when plateau pressures are not high. *Am J Respir Crit Care Med* 2005;172: 1241–1245.
7. Villar J, Martín-Rodríguez C, Domínguez-Berrot AM, Fernández L, Ferrando C, Soler JA, *et al.*; Spanish Initiative for Epidemiology, Stratification and Therapies for ARDS (SIESTA) Investigators Network. A quantile analysis of plateau and driving pressures: effects on mortality in patients with acute respiratory distress syndrome receiving lung-protective ventilation. *Crit Care Med* 2017;45:843–850.
8. Briel M, Meade M, Mercat A, Brower RG, Talmor D, Walter SD, *et al.* Higher vs lower positive end-expiratory pressure in patients with acute lung injury and acute respiratory distress syndrome: systematic review and meta-analysis. *JAMA* 2010;303:865–873.
9. Hotchkiss JR, Blanch L, Murias G, Adams AB, Olson DA, Wangensteen OD, *et al.* Effects of decreased respiratory frequency on ventilator-induced lung injury. *Am J Respir Crit Care Med* 2000;161:463–468.
10. Vaporidi K, Voloudakis G, Priniannakis G, Kondili E, Koutsopoulos A, Tsatsanis C, *et al.* Effects of respiratory rate on ventilator-induced lung injury at a constant Pa_{CO_2} in a mouse model of normal lung. *Crit Care Med* 2008;36:1277–1283.
11. Gattinoni L, Tonetti T, Cressoni M, Cadringher P, Herrmann P, Moerer O, *et al.* Ventilator-related causes of lung injury: the mechanical power. *Intensive Care Med* 2016;42:1567–1575.
12. Cressoni M, Gotti M, Chiurazzi C, Massari D, Algieri I, Amini M, *et al.* Mechanical power and development of ventilator-induced lung injury. *Anesthesiology* 2016;124:1100–1108.
13. Johnson AEW, Pollard TJ, Shen L, Lehman LW, Feng M, Ghassemi M, *et al.* MIMIC-III, a freely accessible critical care database. *Sci Data* 2016; 3:160035.
14. Stewart TE, Meade MO, Cook DJ, Granton JT, Hodder RV, Lapinsky SE, *et al.*; Pressure- and Volume-Limited Ventilation Strategy Group.

Evaluation of a ventilation strategy to prevent barotrauma in patients at high risk for acute respiratory distress syndrome. *N Engl J Med* 1998;338: 355–361.

15. Brower RG, Lanken PN, MacIntyre N, Matthay MA, Morris A, Ancukiewicz M, *et al.*; National Heart, Lung, and Blood Institute ARDS Clinical Trials Network. Higher versus lower positive end-expiratory pressures in patients with the acute respiratory distress syndrome. *N Engl J Med* 2004;351:327–336.
16. Mercat A, Richard J-CM, Vielle B, Jaber S, Osman D, Diehl J-L, *et al.*; Expiratory Pressure (Express) Study Group. Positive end-expiratory pressure setting in adults with acute lung injury and acute respiratory distress syndrome: a randomized controlled trial. *JAMA* 2008;299:646–655.
17. Meade MO, Cook DJ, Guyatt GH, Slutsky AS, Arabi YM, Cooper DJ, *et al.*; Lung Open Ventilation Study Investigators. Ventilation strategy using low tidal volumes, recruitment maneuvers, and high positive end-expiratory pressure for acute lung injury and acute respiratory distress syndrome: a randomized controlled trial. *JAMA* 2008;299:637–645.
18. Cavalcanti AB, Suzumura ÉA, Laranjeira LN, Paisani D de M, Damiani LP, Guimarães HP, *et al.*; Writing Group for the Alveolar Recruitment for Acute Respiratory Distress Syndrome Trial (ART) Investigators. Effect of lung recruitment and titrated Positive End-Expiratory Pressure (PEEP) vs low PEEP on mortality in patients with acute respiratory distress syndrome: a randomized clinical trial. *JAMA* 2017;318:1335–1345.
19. Preacher KJ, Hayes AF. Asymptotic and resampling strategies for assessing and comparing indirect effects in multiple mediator models. *Behav Res Methods* 2008;40:879–891.
20. Zhang Z, Zheng B, Liu N, Ge H, Hong Y. Mechanical power normalized to predicted body weight as a predictor of mortality in patients with acute respiratory distress syndrome. *Intensive Care Med* 2019;45:856–864.
21. Protti A, Andreis DT, Monti M, Santini A, Sparacino CC, Langer T, *et al.* Lung stress and strain during mechanical ventilation: any difference between statics and dynamics? *Crit Care Med* 2013;41:1046–1055.
22. Kallet RH, Alonso JA, Pittet J-F, Matthay MA. Prognostic value of the pulmonary dead-space fraction during the first 6 days of acute respiratory distress syndrome. *Respir Care* 2004;49:1008–1014.
23. Nuckton TJ, Alonso JA, Kallet RH, Daniel BM, Pittet J-F, Eisner MD, *et al.* Pulmonary dead-space fraction as a risk factor for death in the acute respiratory distress syndrome. *N Engl J Med* 2002;346:1281–1286.
24. Hernán MA, Robins JM. Using big data to emulate a target trial when a randomized trial is not available. *Am J Epidemiol* 2016;183: 758–764.
