# Supplementary material for: Electrical impedance tomography–guided individualized ventilation strategy in patients with trauma-related and postoperative acute respiratory distress syndrome
Source: PLoS One. 2026 Aug 24;21(8):e0355609. doi: 10.1371/journal.pone.0355609 (PMC13502546; doi:10.1371/journal.pone.0355609)
Supplement: Supplementary file 3 [file pone.0355609.s010.pdf]

Personalised mechanical ventilation tailored to lung morphology versus low positive end-expiratory pressure for patients with acute respiratory distress syndrome in France (the LIVE study): a multicentre, single-blind, randomised controlled trial

*Jean-Michel Constantin, Matthieu Jabaudon, Jean-Yves Lefrant, Samir Jaber, Jean-Pierre Quenot, Olivier Langeron, Martine Ferrandière, Fabien Grelon, Philippe Seguin, Carole Ichai, Benoit Veber, Bertrand Souweine, Thomas Uberti, Sigismond Lasocki, François Legay, Marc Leone, Nathanael Eisenmann, Claire Dahyot-Fizelier, Hervé Dupont, Karim Asehnoune, Achille Sossou, Gérald Chanques, Laurent Muller, Jean-Etienne Bazin, Antoine Monsel, Lucile Borao, Jean-Marc Garcier, Jean-Jacques Rouby, Bruno Pereira, Emmanuel Futier, for the AZUREA Network**

Lancet Respir Med 2019; 7: 870–80

Published Online August 6, 2019 http://dx.doi.org/10.1016/S2213-2600(19)30138-9

See Comment page 830

*Members listed in the appendix

Department of Perioperative Medicine, University Hospital of Clermont-Ferrand, Clermont-Ferrand, France (J-M Constantin MD, M Jabaudon MD, J-E Bazin MD, E Futier MD); Laboratoire Génétique, Reproduction, et Développement, Centre National de la Recherche Scientifique (CNRS), Institut National de la Santé et de la Recherche Médicale (INSERM), Université Clermont Auvergne, Clermont-Ferrand, France (J-M Constantin, M Jabaudon, J-E Bazin, E Futier); Department of Radiology, Estaing Hospital, University Hospital of Clermont-Ferrand, Clermont-Ferrand, France (J-M Garcier MD); Biostatistical Unit, Department of Clinical Research and Innovation, University Hospital of Clermont-Ferrand, Clermont-Ferrand, France (L Borao PhD, B Pereira PhD); Service des Réanimations, Pôle Anesthésie Douleur Urgences Réanimation, Centre Hospitalier Universitaire de Nîmes, Nîmes, France (J-Y Lefrant MD, L Muller MD); Montpellier University Hospital, Saint Eloi Intensive Care Unit and PhyMedExp, University of Montpellier, INSERM, CNRS, Montpellier, France (S Jaber MD, G Chanques MD); Department of Intensive Care, François

Summary

Background The effect of personalised mechanical ventilation on clinical outcomes in patients with acute respiratory distress syndrome (ARDS) remains uncertain and needs to be evaluated. We aimed to test whether a mechanical ventilation strategy that was personalised to individual patients' lung morphology would improve the survival of patients with ARDS when compared with standard of care.

Methods We designed a multicentre, single-blind, stratified, parallel-group, randomised controlled trial enrolling patients with moderate-to-severe ARDS in 20 university or non-university intensive care units in France. Patients older than 18 years with early ARDS for less than 12 h were randomly assigned (1:1) to either the control group or the personalised group using a minimisation algorithm and stratified according to the study site, lung morphology, and duration of mechanical ventilation. Only the patients were masked to allocation. In the control group, patients received a tidal volume of 6 mL/kg per predicted bodyweight and positive end-expiratory pressure (PEEP) was selected according to a low PEEP and fraction of inspired oxygen table, and early prone position was encouraged. In the personalised group, the treatment approach was based on lung morphology; patients with focal ARDS received a tidal volume of 8 mL/kg, low PEEP, and prone position. Patients with non-focal ARDS received a tidal volume of 6 mL/kg, along with recruitment manoeuvres and high PEEP. The primary outcome was 90-day mortality as established by intention-to-treat analysis. This study is registered online with ClinicalTrials.gov, NCT02149589.

Findings From June 12, 2014, to Feb 2, 2017, 420 patients were randomly assigned to treatment. 11 patients were excluded in the personalised group and nine patients were excluded in the control group; 196 patients in the personalised group and 204 in the control group were included in the analysis. In a multivariate analysis, there was no difference in 90-day mortality between the group treated with personalised ventilation and the control group in the intention-to-treat analysis (hazard ratio [HR] 1·01; 95% CI 0·61–1·66; p=0·98). However, misclassification of patients as having focal or non-focal ARDS by the investigators was observed in 85 (21%) of 400 patients. We found a significant interaction between misclassification and randomised group allocation with respect to the primary outcome (p<0·001). In the subgroup analysis, the 90-day mortality of the misclassified patients was higher in the personalised group (26 [65%] of 40 patients) than in the control group (18 [32%] of 57 patients; HR 2·8; 95% CI 1·5–5·1; p=0·012.

Interpretation Personalisation of mechanical ventilation did not decrease mortality in patients with ARDS, possibly because of the misclassification of 21% of patients. A ventilator strategy misaligned with lung morphology substantially increases mortality. Whether improvement in ARDS phenotyping can decrease mortality should be assessed in a future clinical trial.

Funding French Ministry of Health (Programme Hospitalier de Recherche Clinique InterRégional 2013).

Copyright © 2019 Elsevier Ltd. All rights reserved.

Introduction

Acute respiratory distress syndrome (ARDS) is a potentially devastating complication of many pulmonary and non-pulmonary pathologies that is associated with high mortality and low long-term quality of life.[1,2] International guidelines have recommended the use of protective lung ventilation[3] featuring low tidal volume, reduced inspiratory plateau pressure (Pplat),[4] low driving pressure, and prone position to limit ventilator-induced lung injury.[5] However, there is uncertainty about the effects of higher

Research in context

Evidence before this study
Only a few studies have shown an improvement in mortality and major outcomes in patients with acute respiratory distress syndrome (ARDS). One reason being that all types of ARDS are not the same and should not be treated in the same way. There are two ways to phenotype patients with ARDS. First, biological phenotypes that have been described in US-based randomised control trials; these phenotypes have different responses to mechanical ventilation or drugs but have never been assessed prospectively. Second, the use of surrogates of recruitability assessed by CT scan. Numerous physiological studies have shown that responses to mechanical ventilation were different in terms of recruited volume or hyperinflation. We searched PubMed on Jan 12, 2018, for randomised control trials written in English with the keywords "ARDS" and "phenotypes" or "personalisation". We identified no previous studies.

Added value of this study
This study reports that prospective assessment of ARDS phenotypes is not easy using routine techniques. When mechanical ventilation and phenotypes are aligned, mortality might decrease. But when mechanical ventilation and phenotypes are misaligned, mortality increases substantially.

Implications of all the available evidence
Our findings suggest that phenotypes of ARDS should be taken into account both in clinical practice and for further studies. The large difference in mortality related to phenotypes, independent of ventilation strategy, might modify the results of trials on drugs or other treatment strategies. The differences in terms of response to ventilator setting, and the difficulty to correctly phenotype patients at the bedside, warrant further research. The results of this study provide key information that might yield progress in different fields of critical care, but to correctly assign the ventilation strategy for critically ill patients requires rapid phenotyping often by one physician alone, which can be challenging. Researchers should focus on these points for further research into the critical care of patients with ARDS.

positive end-expiratory pressure (PEEP) and recruitment manoeuvres.[6–8] In one randomised clinical trial,[9] the effects of high PEEP and recruitment manoeuvres, known as the open-lung approach, was evaluated in patients with ARDS; results showed an increase in mortality in the intervention group compared with patients in whom PEEP was set using the well known PEEP and FiO_2 table (low PEEP). Even if the results of this trial are clear, the discussion surrounding open-lung ventilation is not closed for several reasons.[10] ARDS is a heterogeneous syndrome involving different subgroups (phenotypes) with distinct clinical and outcome characteristics.[11,12] Patients with non-focal ARDS (defined as patients with diffuse and patchy loss of aeration), as assessed by chest CT, have higher mortality and distinct respiratory mechanics, including lower lung compliance and a higher amount of recruitable lung, than patients with focal ARDS.[13–15] This heterogeneity could have obscured the effects of some therapies and might explain why no benefit for higher PEEP and recruitment manoeuvres was identified in previous trials.[16] Consequently, the response to PEEP and recruitment manoeuvres might vary considerably between patients as a function of lung morphology.[17] International guidelines on mechanical ventilation for patients with ARDS encourage the personalisation of clinical decisions, but the way in which personalisation can be achieved is still unknown.[18,19] According to physiological studies on alveolar recruitment,[13–15] higher PEEP and recruitment manoeuvres might be more suitable in non-focal rather than focal ARDS, whereas the patients with focal ARDS who responded poorly to high PEEP and recruitment manoeuvres might benefit more from prone positioning and lower PEEP. Furthermore, because lung volumes are higher in patients with focal ARDS than in those with non-focal ARDS, the use of higher tidal volume might be more appropriate to limit atelectrauma and inflammation.[20] Prone position is probably as efficient in non-focal ARDS as it is in focal ARDS in terms of gas exchange and recruited volume,[21] but when recruitment manoeuvres and high PEEP have already recruited the lung and increased gas exchange, the effect is probably less important.

We designed the Lung Imaging for Ventilator Setting in ARDS (LIVE) trial[22] to test whether a mechanical ventilation strategy personalised to individual patients' lung morphology (low PEEP, high tidal volume, and early prone position for focal ARDS and recruitment manoeuvres and high PEEP for non-focal ARDS) would improve the survival of patients with ARDS in comparison to the well established low-PEEP strategy.[23]

Methods

Study design

We did this prospective, multicentre, stratified, parallel-group, single-blind randomised controlled trial in 20 university and non-university intensive care units (ICUs) in France (originally 22 centres were included, but two were excluded at the 6-month mark because they did not enrol any patients; appendix pp 4, 5). All investigators were trained during the kick-off meeting for the assessment of lung morphology. The trial protocol and statistical analysis plan (appendix p 9) were approved by a central ethics committee (Comité de Protection des Personnes Sud-EstVI, Clermont-Ferrand, France) and were published elsewhere.[22] The trial was overseen by a

Mitterrand University Hospital, Dijon, France (J-P Quenot MD); **Lipness Team, INSERM Research Centre, and LabExLipSTIC, University of Burgundy, Dijon, France** (J-P Quenot); **INSERM Centres d'Investigation Clinique, Department of Clinical Epidemiology, University of Burgundy, Dijon, France** (J-P Quenot); **Multidisciplinary Intensive Care Unit, Department of Anaesthesiology and Critical Care, Pitié-Salpêtrière Hospital, Assistance Publique Hôpitaux de Paris, Paris, France** (O Langeron MD, A Monsel MD, J-J Rouby MD, J-M Constantin); **Department of Anaesthesia and Intensive Care Medicine, Regional University Centre of Tours, Tours, France** (M Ferrandière MD); **Service de Réanimation, General Hospital Le Mans, Le Mans, France** (F Grelon MD); **Department of Intensive Care Medicine, University Hospital of Rennes, University of Rennes 1 and Unité Mixte de Recherche NuMeCan, Rennes, France** (P Seguin MD); **Intensive Care Unit, University Hospital of Nice, Université Côte d'Azur, Nice, France** (C Ichai MD); **Pôle Anesthésie-Réanimation-SAMU, Rouen University Hospital, Rouen, France** (B Veber MD); **Service de Médicine Intensive et Réanimation, University Hospital of Clermont-Ferrand, Clermont-Ferrand, France** (B Souweine MD); **Department of Anaesthesiology and Intensive Care Medicine, Edouard Herriot Hospital, Hospices Civils de Lyon, Lyon, France** (T Uberti MD); **Department of Anaesthesiology and Reanimation, University Hospital Angers, Angers, France** (S Lasocki MD); **Service de Réanimation, Hospital General Saint-Brieuc, Saint-Brieuc, France** (F Legay MD); **Service d'Anesthésie-Réanimation, Assistance Publique-Hôpitaux de Marseille, Hôpital Nord, Aix-Marseille Université, Marseille, France** (M Leone MD); **Department of Anaesthesiology and Critical Care, Centre Régional de Lutte Contre le Cancer Jean Perrin, Clermont-Ferrand, France** (N Eisenmann MD); **Department**

of Anaesthesia-Intensive Care and Perioperative Medicine, University Hospital of Poitiers, University of Poitiers, INSERM, Poitiers, France (C Dahyot-Fizelier MD); Unité de Recherche Clinique Simplification des Soins chez les Patients Complexes, Université Jules Verne de Picardie, Service d'Anesthésie-Réanimation, Centre Hospitalier Universitaire d'Amiens Picardie, Amiens, France (H Dupont MD); Service d'Anesthésie Réanimation Chirurgicale, Centre Hospitalier Universitaire de Nantes, Hôtel Dieu-HME Hospital, Nantes, France (K Asehnoune MD); and Service de Réanimation, Centre Hospitalier Général du Puy-en-Velay, Puy-en-Velay, France (A Sossou MD)

Correspondence to: Dr Jean-Michel Constantin, Department of Perioperative Medicine, University Hospital of Clermont-Ferrand, 63003 Clermont-Ferrand, France jmconstantin@chu-clermontferrand.fr

See Online for appendix

steering committee. An independent data and safety monitoring board, comprised of three experts in the field, was also created (appendix p 31). No commercial support was received for this project.

Patients

Adult patients older than 18 years who had a diagnosis of moderate-to-severe ARDS for less than 12 h, defined according to the Berlin definition[24] (partial pressure of arterial oxygen [PaO_2]:FiO_2 ratio ≤200 mm Hg with a PEEP ≥5 cm H_2O on volume control ventilation in a patient who was sedated and paralysed at steady state), were eligible for inclusion in the study if they also had a French social security number and had provided written informed consent. Exclusion criteria were mechanical ventilation for more than 7 consecutive days in the past 30 days; a previous history of ARDS in the previous month; intracranial hypertension; a body-mass index higher than 40 kg/m²; chronic respiratory diseases requiring long-term oxygen therapy or respiratory assistance; allogeneic bone-marrow transplantation; metastatic cancer; extensive burns; liver cirrhosis with Child Pugh class C; broncho-pleural fistula; pulmonary fibrosis; patients who were moribund, pregnant, or facing end of life; patients under tutelage; and patients already enrolled in another interventional study on ARDS. Written informed consent was obtained from the patient's next of kin.

Randomisation and masking

Enrolment, randomisation, and data collection were done using an online system. Randomisation was computer-generated with the use of a minimisation algorithm with a random component of 80% and was stratified according to the study site, lung morphology, and duration of mechanical ventilation (≤48 h or >48 h) before ARDS diagnosis. Patients were assigned in a 1:1 ratio to a ventilation strategy adjusted on the basis of lung morphology (personalised group) or to a standard strategy in line with traditional care (control group). Lung morphology was assessed (by a local investigator) before randomisation using a CT scan of the whole lung or chest x-ray when the severity of the patient was not compatible with transport between hospitals. Patients were characterised as having focal ARDS (presence of consolidations localised only in the lower and back part of the lungs) or non-focal ARDS by local site investigators (appendix p 8).[13,25,26] As defined in the protocol, all lung images were re-evaluated a posteriori by adjudicators (one radiologist and two intensivists) who were masked to patient history and group allocation. All CT scan and chest x-rays were anonymous, identified only by the random-isation number.

	Control group (n=204)	Personalised group (n=196)	
		Focal lung morphology	Non-focal lung morphology
Mode of ventilation	Volume control	Volume control	Volume control
Tidal volume	6 mL/kg IBW	8 mL/kg IBW	6 mL/kg IBW
PEEP	PEEP/FiO_2	5–9 cm H_2O	To reach Pplat of 30 cm H_2O
PEEP-PSV	Free	5–9 cm H_2O	≥10 cm H_2O
Recruitment manoeuvre	Rescue	Rescue	Mandatory
Prone position	Encouraged	Mandatory	Rescue

IBW=ideal body weight. PEEP=positive-end expiratory pressure. FiO_2=fraction of inspired oxygen. Pplat=end-inspiratory plateau pressure. PEEP-PSV=positive-end expiratory pressure used during pressure support ventilation.

Table 1: **Summary of ventilator settings according to lung morphology and randomisation group**

Procedures

Mechanical ventilation (table 1) was delivered in a volume-controlled manner with FiO_2 set to allow for oxy-haemoglobin saturation values higher than 88% or PaO_2 higher than 55 mm Hg.[3] All patients received deep sedation assessed with the Richmond Agitation and Sedation Scale[27] and early neuromuscular blockade for a period of no more than 48 h.[28]

Patients assigned to the control group received, regardless of lung morphology, a tidal volume of 6 mL/kg of predicted bodyweight and a PEEP selected according to the low PEEP and FiO_2 table from the ALVEOLI[28] study (appendix p 11), while maintaining an end-inspiratory Pplat of no more than 30 cm of water. For patients assigned to the personalised group, the ventilator settings were adjusted on the basis of lung morphology. Patients with focal ARDS received a tidal volume of 8 mL/kg predicted bodyweight with a PEEP of 5–10 cm of water, according to the oxygenation targets. Patients with non-focal ARDS received a tidal volume of 6 mL/kg predicted bodyweight and PEEP was adjusted to reach a Pplat of 30 cm of water.[7] Immediately afterwards, recruitment manoeuvres were applied, and PEEP was titrated at the highest value without increasing Pplat to more than 30 cm of water. Recruitment manoeuvres were at the discretion of the clinician and included a continuous positive airway pressure of at least 35 cm H_2O for at least 30 sec or an extended sight.[29] The recruitment procedure could be repeated at the clinician's discretion or, in cases of desaturation, after tidal volume was reduced to 4 mL/kg predicted bodyweight and PEEP was readjusted. Prone position was mandatory in the personalised group for patients with focal ARDS, was encouraged for all patients of the control group, and usable only as rescue therapy in the personalised group for patients with non-focal ARDS. Prone position sessions were at least 16 h and were stopped when patients were put on pressure support ventilation.[30] Apart from the ventilation strategy, other aspects of care were similar for both groups. Weaning from mechanical ventilation and interruption of sedation were done similarly for both groups (appendix p 21).

Outcomes

The primary outcome was all-cause mortality at day 90 (with day 0 corresponding to ARDS diagnosis). Assessments were done centrally, but all case report forms

were monitored for data accuracy by a clinical research associate. Secondary outcomes were mortality at 28 days, 30 days, 180 days, and 365 days, ICU mortality, number of ventilator-free days at day 30, ARDS resolution, length of stay in the ICU, the number of patients with ventilator-associated pneumonia, and barotrauma (pneumothorax and pneumomediastinum). The concentrations of soluble forms of the receptor for advance glycation end products (ie, soluble Receptor for Advanced Glycation End-products and endogenous secretory Receptor for Advanced Glycation End-products) and quality of life at 1 year were also assessed but data are not reported here. Although hospital length of stay and mortality were prespecified as secondary outcomes, data were not collected on them because the required fields were omitted in error from the data collection forms. Successful extubation was defined as no reintubation or the use of non-invasive ventilation in the 48 h after extubation.[31] For patients who had a tracheotomy, successful weaning from mechanical ventilation was defined as the ability to breathe unassisted through the tracheostomy cannula for at least 48 h.[28] Safety and serious adverse events were pneumothorax, severe hypoxaemia, defined as a PaO_2:FiO_2 of less than 50 mm Hg, and death.

Statistical analysis

We calculated that a sample of 420 patients would provide the trial with 90% power to show an absolute between-group difference of 13% in the primary outcome measure at a two-sided α level of 0·05, assuming a 33% 90-day mortality (Freedman method).[16] For safety reasons, an interim analysis was done after 50% of patients were enrolled (Lan and Demets method).[32] Results of the interim analysis were communicated only to the data monitoring and safety board.

We did an intention-to-treat analysis, which included all participants who were randomly assigned to treatment, except those who withdrew consent and those who were found to be ineligible because they met the exclusion criteria. We used an unadjusted log-rank test for the primary analysis. We did the adjusted analyses using marginal Cox proportional-hazards regression to take into account adjustments of covariates selected according to univariate results (appendix p 18), clinical relevance, and variables used for stratification, and to consider within-centre and between-centre variability. The results were expressed as hazard ratios (HRs) with 95% CIs, and the proportional hazard assumption was verified using the Schoenfeld test. The unadjusted χ^2 or Fisher's exact test was used for binary endpoints as appropriate. Continuous variables were compared with the use of unpaired *t* tests or Mann–Whitney *U* tests. Adjusted analyses were done using the same adjustment variables as described previously in the regression models, including a random effect to account for the centre effect. The results were expressed as relative risk with 95% CIs for dichotomous endpoints and as regression coefficients with 95% CIs for continuous endpoints. The adjudicators' diagnostic accuracy of distinguishing focal ARDS from non-focal ARDS was done after inclusion of the last patient and

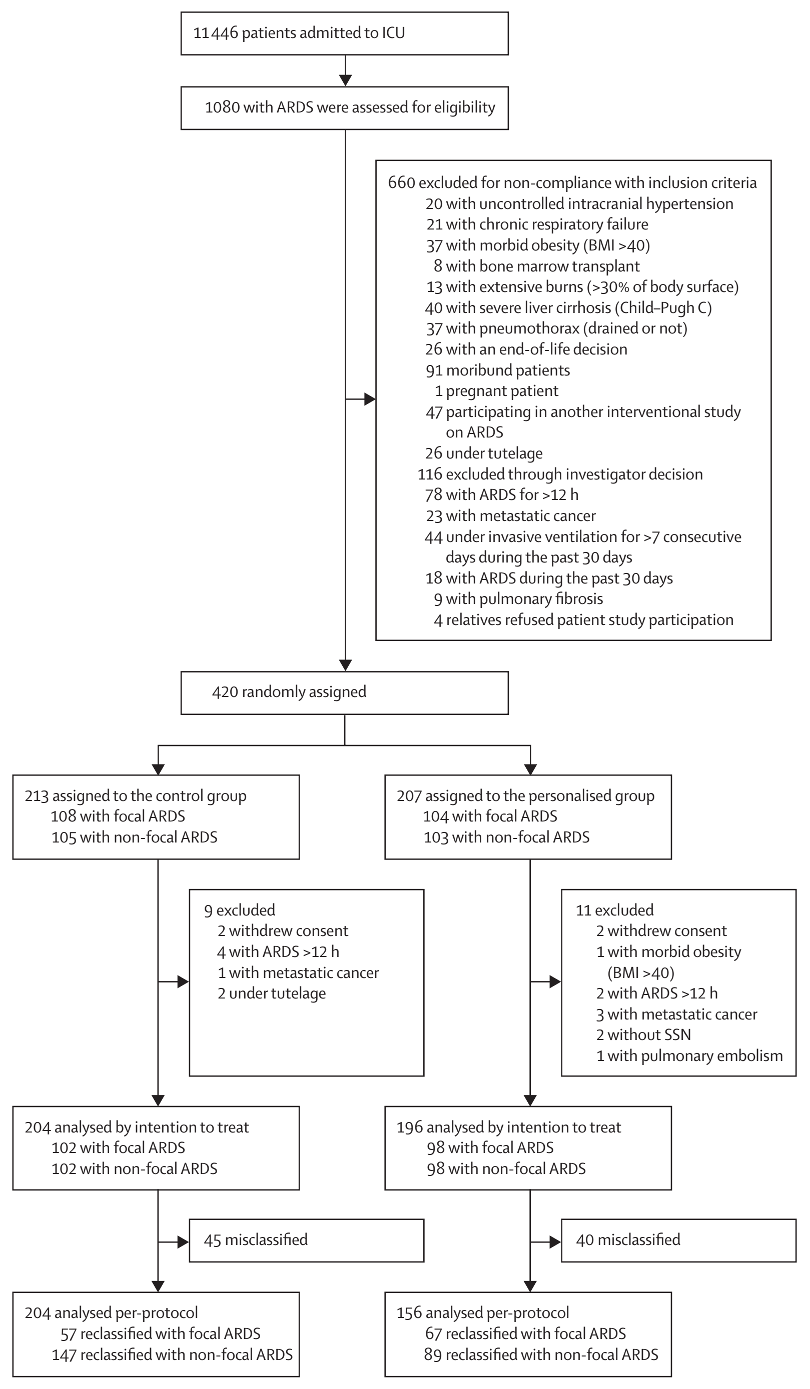

Figure 1: **Trial profile of the LIVE study**
85 (21%) of 400 patients were misclassified (45 [22%] of 204 patients in the control group and 40 [20%] of 196 patients in the personalised group). For the per-protocol analysis, the 40 patients with misclassified lung morphology who were included in the personalised group were excluded from the analysis. The 45 misclassified patients of the control group were not excluded because they were not misaligned with ventilator strategy. ARDS=acute respiratory distress syndrome. BMI=body-mass index. ICU=intensive care unit. SSN=social security number.

	Control group (n=204)	Personalised group (n=196)
Sex		
Male	147 (72%)	154 (79%)
Female	57 (28%)	42 (21%)
Age (years)	61 (16)	63 (14)
BMI (kg/m²)	26 (5)	26 (5)
Predicted bodyweight (kg)	65 (10)	65 (9)
Simplified Acute Physiology Score 2* at admission	52 (17)	51 (16)
Onset of ventilation (days)	0·6 (1·1)	0·6 (1·2)
Onset of ARDS (h)	5·5 (3·9)	5·8 (4·8)
McCabe score		
A	137 (67%)	126 (64%)
B	51 (25%)	58 (30%)
C	10 (5%)	5 (3%)
Comorbidities		
COPD	14 (7%)	26 (13%)
Haematological cancer	10 (5%)	6 (3%)
Solid cancer	4 (2%)	1 (1%)
Chronic kidney disease	3 (1%)	3 (2%)
Other	125 (61%)	143 (73%)
None	62 (30%)	39 (20%)
Cause of ICU admission		
Septic shock	33 (16%)	41 (21%)
Haemorrhagic shock	9 (4%)	8 (4%)
Coma	11 (5%)	13 (7%)
Intra-abdominal sepsis	18 (9%)	16 (8%)
Traumatic injuries	14 (7%)	7 (4%)
Acute respiratory failure	94 (46%)	81 (41%)
Acute metabolic disorders	6 (3%)	4 (2%)
Elective surgery	14 (7%)	15 (8%)
Urgent surgery	5 (2%)	11 (6%)
Cause of ARDS		
Pulmonary	148 (73%)	144 (73%)
Extrapulmonary	56 (27%)	52 (27%)
Coexisting conditions		
Norepinephrine	123 (60%)	123 (63%)
Renal replacement therapy	12 (6%)	14 (7%)
Antibiotics	180 (88%)	180 (92%)
Steroids	49 (24%)	42 (22%)

Data are n (%) or mean (SD). BMI=body-mass index. ARDS=acute respiratory distress syndrome. COPD=chronic obstructive pulmonary disease. ICU=intensive care unit. *The Simplified Acute Physiology Score 2 is based on 17 variables; scores range from 0 to 163, with higher scores indicating more severe disease.[33]

Table 2: **Demographic characteristics and coexisting conditions of the patients at admission**

evaluated by means of Fleiss' κ. The adjudicators were masked to clinical history, the centre of inclusion, the allocation group, and the outcomes. These analyses were completed by multidimensional factorial analysis (factorial mixed-data analysis to analyse assets as elements of qualitative and quantitative variables) to uncover the underlying relationships and structure (latent constructs) and to aggregate patients into clusters, such that each cluster represents a topic (randomised groups and misclassification), to establish whether groups (randomised groups and misclassification) were characterised by qualitative and quantitative variables chosen according to univariate results and to clinical relevance.

In the prespecified per-protocol analysis, misclassified patients in the personalised group were excluded. Misclassified patients of the control group were not excluded because they were not misaligned with ventilator strategy, which by definition is not related to lung morphology. A secondary analysis was also done, removing all misclassified patients.

Because less than 5% of the data were missing, handling of missing data was not done in this study, and we did a complete case analysis. All analyses were done with the use of STATA software, version 13.0. A two-sided p value of less than 0·05 was considered to indicate statistical significance. This study is registered online with ClinicalTrials.gov, number NCT02149589.

Role of the funding source

The funders of the study had no role in study design, data collection, data analysis, data interpretation, or writing of the report. BP and LB had full access to all the data in the study and J-MC had final responsibility for the decision to submit for publication.

Results

From June 12, 2014, to Feb 2, 2017, a total of 1080 patients with ARDS were assessed for eligibility. After 660 patients were excluded because they were not eligible to participate, 420 were randomly assigned to treatment (figure 1). Nine patients were subsequently excluded in the control group and 11 patients were excluded in the personalised group; therefore, 400 patients were included in the analysis (196 in the personalised group and 204 in the control group). The two groups were similar at baseline (table 2; appendix p 12). CT scanning was used to assess lung morphology for 56 (29%) of 196 patients in the personalised group and 80 (39%) of 204 patients in the control group (appendix p 12). Agreement on disease classification between the three experts regarding diagnosis of focal ARDS and non-focal ARDS was high (κ=0·94), and the diagnoses were unanimous for all but two patients. However, for local investigators, the agreement was moderate (κ=0·52; figure 1). There was no significant difference in the baseline characteristics of the correctly classified patients and the misclassified patients (appendix p 22).

All patients were sedated and paralysed at study inclusion. After randomisation, 82 (84%) of 98 patients with non-focal ARDS from the personalised group had at least one recruitment manoeuvre (median 3 [IQR 0–5]). 17 (3%) of 503 recruitment manoeuvres were interrupted

	Control group (n=204)		Personalised group (n=196)	
	Focal ARDS (n=102)	Non-focal ARDS (n=102)	Focal ARDS (n=98)	Non-focal ARDS (n=98)
Tidal volume (mL)	398 (71)	398 (89)	486 (74)	392 (78)
Tidal volume (mL/kg PBW)	6·0 (1·0)	6·2 (1·1)	7·3 (1·1)	6·3 (1·0)
Respiratory rate (breaths/min)	26 (4)	27 (4)	22 (5)	27 (5)
PEEP (cm water)	11 (2)	10 (2)	8 (2)	14 (3)
FiO_2 (%)	56 (16)	53 (17)	48 (19)	49 (15)
Respiratory system Pplat (cm water)	21 (4)	22 (6)	20 (5)	26 (6)
P_{max} (cm water)	33 (7)	35 (7)	35 (9)	37 (7)
Respiratory system Cst (mL/cm water)	41 (15)	36 (14)	40 (10)	34 (15)
Driving pressure (cm water)	9 (5)	11 (5)	11 (5)	12 (5)
SaO_2 (%)	95 (6)	96 (4)	97 (3)	97 (3)
PaO_2 (mm Hg)	94 (37)	92 (30)	94 (31)	110 (43)
PaO_2:FiO_2 (mm Hg)	179 (83)	189 (80)	217 (90)	240 (102)
$PaCO_2$ (mm Hg)	42 (8)	42 (8)	38 (10)	44 (10)
Arterial blood (pH)	7·35 (0·09)	7·38 (0·09)	7·39 (0·09)	7·35 (0·10)
Lactate (mmol/L)	2·4 (2·0)	2·2 (2·1)	2·0 (1·7)	1·8 (1·6)
Plasma bicarbonate (mmol/L)	23 (4)	24 (4)	23 (5)	24 (4)

Data are mean (SD). Driving pressure was calculated as Pplat minus PEEP.[4] ARDS=acute respiratory distress syndrome. PBW=predicted bodyweight. PEEP=positive-end respiratory presssure. FiO_2=fraction of inspired oxygen. Pplat=end-respiratory plateau pressure. Pmax=maximal inspiratory pressure. Cst=static compliance. SaO_2=oxygen saturation. PaO_2=partial pressure of arterial oxygen. $PaCO_2$=partial pressure of arterial carbon dioxide. PaO_2:FiO_2=ratio of the partial pressure of arterial oxygen to the fraction of inspired oxygen.

Table 3: **Ventilator settings, respiratory system mechanics, and results of arterial-blood gas measurements after randomisation (day 1)**

because of hypotension or desaturation. In these patients with non-focal ARDS, the average PEEP was 14 cm (SD 3) of water compared with 10 cm (2) of water for those in the control group. Among the 98 patients with focal ARDS in the personalised group, 92 (94%) had at least one prone-position session (median 2 [1–2]). In these patients with focal ARDS, tidal volume was 7 mL/kg (SD 1) of predicted bodyweight and PEEP was 8 cm (SD 2) of water compared with 6 mL/kg (1) of predicted bodyweight and a PEEP of 11 cm (2) of water for patients with focal ARDS in the control group (table 3). In the control group, 52 (25%) of 204 patients had a prone-position session at least once (appendix p 19).

At day 90, 56 (27%) of 204 individuals died in the control group and 53 (27%) of 196 died in the personalised group. No significant difference between the two groups was shown by univariate analysis (HR 0·96; 95% CI 0·66–1·4; p=0·84; figure 2A) or multivariable analysis (HR 1·01; 0·61–1·66; p=0·98). There was no interaction between recruitment manoeuvres and mortality (HR 0·9; 0·60–1·3; p=0·64) between the two groups. When the interaction between recruitment manoeuvres and mortality was assessed, taking into account lung morphology, a positive interaction was recorded for patients with non-focal ARDS (HR 0·63; 0·39–1·02; p=0·062) and a negative interaction was recorded for those with focal ARDS (HR 3·45; 1·4–8·5; p=0·019). There was no significance difference in secondary outcomes when combining all univariate and multivariable analyses, except rescue therapies (figure 3).

In a prespecified analysis taking into account classification by experts, done for all enrolled patients (correctly classified and misclassified), 90-day survival was improved in the personalised group (n=196) compared with the control group (n=204; HR 0·58; 95% CI 0·37–0·93; p=0·024; appendix p 25) using a marginal Cox model adjusted for misclassification (yes or no categories). We found a significant interaction between misclassification and randomised group allocation with respect to the primary outcome (p<0·001). Thus, in the subgroup analysis, the 90-day mortality of the misclassified patients was higher in the personalised group (26 [65%] of 40 patients) than in the control group (18 [32%] of 57 patients; HR 2·8; 95% CI 1·5–5·1; p=0·012). Mortality was lower in the personalised group (29 [19%] of 156 patients) than in the control group (58 [28%] of 204 patients) for the correctly classified patients (0·6; 0·37–0·99; p=0·042), as established in the univariate analysis (figure 2). In misclassified patients, we noted an interaction between focal lung morphology and mortality (HR 8·65; 1·43–52·18; p=0·019). According to the factorial mixed-data analysis, the four groups that were studied overlapped, showing that they could be considered similar at baseline (ie, non-focal correctly classified, focal correctly classified, non-focal misclassified, and focal misclassified; appendix p 23). Comparison of baseline characteristics, ventilator setting, and outcomes according to group and classification are displayed in the appendix (p 17).

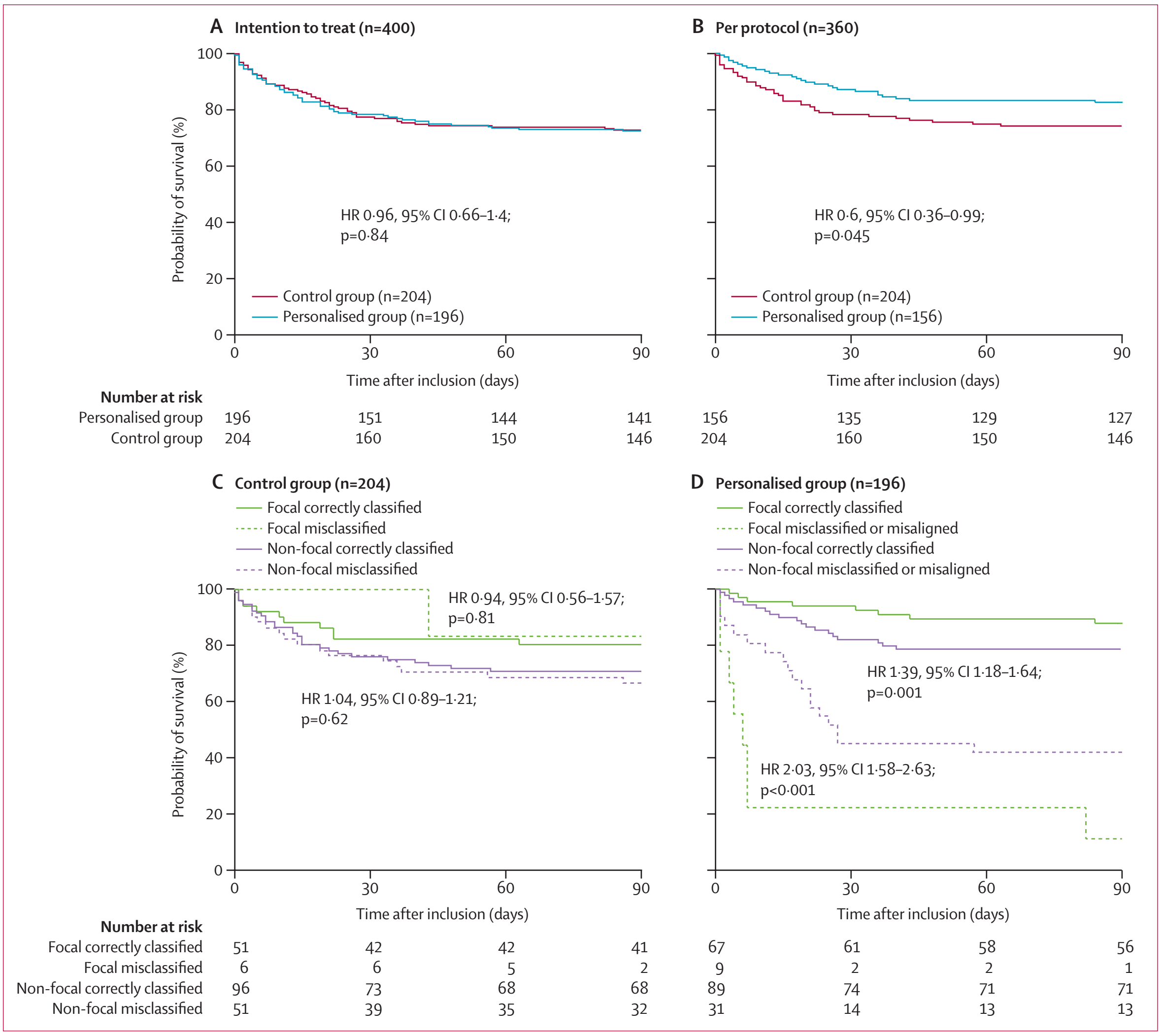

Figure 2: **Effect of personalised ventilator strategy on overall survival at day 90**
In patients classified as having focal ARDS at the time of randomisation, moderate PEEP associated with early prone position was implemented. In patients classified as having non-focal ARDS at the time of randomisation, high PEEP associated with recruitment manoeuvres was implemented. (A) Survival as established by the intention-to-treat analysis, considering all the patients, regardless of whether they were correctly classified or misclassified. (B) Survival as established by the per-protocol analysis, only considering patients who were correctly classified in the intervention group. (C) Survival for patients with focal ARDS who were correctly classified or misclassified and those with non-focal ARDS who were correctly classified or misclassified, ventilated according to the low-PEEP strategy (control group). (D) Survival for patients with focal ARDS who were correctly classified or those with focal ARDS who were misclassified and who were misaligned with the ventilator strategy, and patients with non-focal ARDS who were correctly classified or misclassified and who were misaligned with the incorrect ventilator strategy (personalised group). A comparison of survival for patients correctly classified and misclassified on the basis of group randomisation is provided in the appendix (p 19). ARDS=acute respiratory distress syndrome. HR=hazard ratio. PEEP=positive end-expiratory pressure.

The causes of death and secondary outcomes are summarised in the appendix (p 14). Barotrauma (pneumothorax in five [2%] of 204 patients in the control group *vs* five [3%] of 196 patients in the personalised group; p=0·63), fluid loading in the first 3 days (2277 mL [SD 1800] in the control group *vs* 2411 mL [2579] in the personalised group; p=0·76), norepinephrine used, or cardiac arrest was not different between groups (data not shown). In the per-protocol analysis (without misclassified patients from the personalised group; figure 1), 90 day mortality was decreased from 56 (27%) of 204 patients in the control group to 27 (17%) of 156 in the personalised group (HR 0·6; 95% CI 0·36–0·99; p=0·045; figure 2B).

Discussion

In patients with moderate-to-severe ARDS, ventilation that is personalised on the basis of patient lung morphology, as assessed by investigators at the time of study inclusion, did not modify mortality compared with classic low tidal volume ventilation applied independently of lung morphology. However, the investigators misclassified lung morphology for 85 (21%) of 400 patients in both groups. High mortality was observed among the misclassified

patients for whom the misaligned ventilator strategy was applied. Analysis of patients whose lung morphology was correctly classified at inclusion (per-protocol analysis) revealed a significant increase in survival for those in the personalised group. This finding suggests that misclassification of lung morphology might have concealed a potential beneficial effect of personalised mechanical ventilation on survival. This reduction in mortality observed when lung morphology and mechanical ventilation are aligned is concordant with what is expected by retrospective analysis of historical cohorts when post-hoc phenotyping is done.[34] The approach by Calfee and colleagues[34] is therefore absolutely valid; all patients with ARDS are not the same, and these different phenotypes need to be considered when setting the ventilator or when choosing drugs for these patients. However, prospectively phenotyping patients with ARDS is more difficult than expected, and when misclassification occurs and patients are not aligned with disease-specific strategies, clinical outcomes are worse than they are when standard strategies are used (low PEEP and low tidal volume).

Because this is a negative study, both the choice of surrogate used for phenotyping and the algorithm for personalisation needs to be discussed. Lung morphology has been extensively studied in the past 30 years in terms of response to PEEP and alveolar recruitment.[14,25,35] Because we had 20 centres, we chose lung morphology for simplicity to assess alveolar recruitment and hyperinflation rather than using quantitative analysis, which would mean the use of two slices at two different levels of pressure. Characterisation of lung morphology cannot be reproduced retrospectively and staff at centres might not be sufficiently trained in assessing lung morphology, which are limitations of using lung morphology to phenotype ARDS. We used lung morphology as a surrogate for phenotyping ARDS for several reasons. First, evidence suggests that patients with focal ARDS did not have alveolar recruitment, whereas those with non-focal ARDS did.[25,29,36] Second, from a pathophysiological point of view, non-focal ARDS is different from focal ARDS in terms of epithelial injury, as highlighted by the receptor for advanced glycation end-products pathway.[37] This epithelial injury is related to alveolar clearance, which might be the endotype that underlies these two phenotypes.[19,26,38,39] Whether or not non-focal and focal ARDS are related to Calfee's phenotype is not known, although associated mortality and response to PEEP are similar for both types.

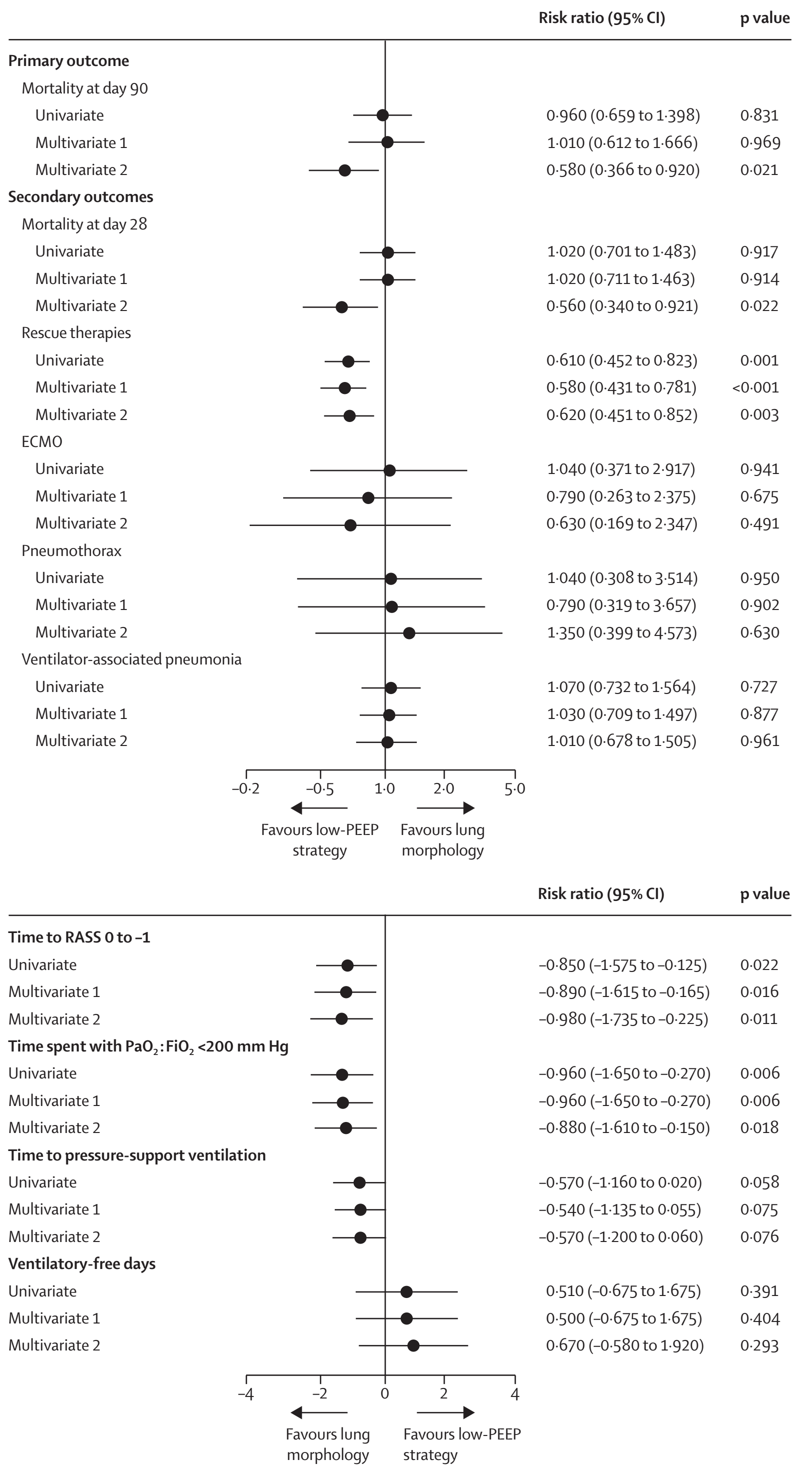

Figure 3: **Primary and secondary outcomes of patients treated on the basis of lung morphology versus low-PEEP strategy**
Univariate and multivariable intention-to-treat analyses of the primary and secondary outcomes done in the overall population (400 patients). Two models were used for the multivariable analysis, with and without the misclassified variable. Note that time to RASS 0 to –1 is the time between study enrolment and achievement of light sedation, defined by a score on the RASS between 0 and –1 according to the study protocol. Time to pressure support ventilation is the time between study enrolment and the switch from volume control ventilation to pressure support ventilation. Ventilator-free days is the number of days a patient was alive and free of mechanical ventilation at day 30. ARDS=acute respiratory distress syndrome. ECMO=extracorporeal membrane oxygenation. FiO_2=fraction of inspired oxygen. PaO_2=partial pressure of arterial oxygen. PEEP=positive end-expiratory pressure. RASS=Richmond Agitation and Sedation Scale.

The overall mortality of the patients included in this trial was lower than that previously published for randomised controlled trials and prospective cohorts.[1,9,28,40,41] However, the patients in the present study had similar amounts of hypoxaemia and multiorgan dysfunction as patients in previous studies.[7,9,28,33,41] A specific characteristic of this study was early inclusion of patients (within the first 12 h of ARDS onset) compared with the timeframe for inclusion in other trials (up to 72 h).[9,28,41,42] Early standardisation of mechanical ventilation might explain the differences in mortality between the present study and previously published cohort studies.[1,40] Notably, in the present study, overall mortality was close to that reported in the PROSEVA trial,[30] in which the mean time from intubation to inclusion was around 32 h. The present trial has another strength, insofar as it is the first to investigate the effects of personalised ventilator settings on mortality. In patients with ARDS, effective adjustment of PEEP on the basis of the physiological features of individual patients has been difficult to achieve. For example, in the ARDSNet study[6] of low tidal volume ventilation, PEEP and FiO_2 were adjusted according to arterial oxygenation without considering chest wall or lung mechanics, and PEEP had no effect on survival. These results have been supported by other studies.[8] The Expiratory Pressure Study Group trial[43] was the first study to personalise PEEP according to respiratory mechanics; the treatment led to improvements in ventilator-free and organ-failure-free days, oxygenation, and respiratory-system compliance, but showed no significant effect on survival. More recently, applying high PEEP and recruitment manoeuvres to patients with moderate-to-severe ARDS was associated with improved oxygenation, but increased mortality.[9] These results might be due, at least in part, to the inclusion of patients with focal ARDS and non-focal ARDS, without consideration of their differences in terms of predicted mortality and distinct responses to PEEP and recruitment manoeuvres.[17,25]

This trial has several limitations. First, our study is open label, as are all other trials on this topic, and performance bias might have generated differences in the use of adjunctive therapies. We tried to reduce these biases by using a precise algorithm. Unfortunately adherence to the protocol was not perfect, as highlighted by the low proportion of patients who had CT scans. However, the use of adjunctive therapies in this trial is in line with available international data.[1] One of the main limitations was the high number of patients whose lung morphology was misclassified by the investigators. This issue was not anticipated, based on our previously published studies,[25,26,44] but it could be explained by the investigators' experience, the low number of CT scans, or the method used. During training sessions of investigators, we did not check that all investigators were present, and we did not formally establish the ability of investigators to assess lung morphology on different examples of both types of ARDS. To decrease the proportion of misclassifications, more formal training could have been set up. CT scans for all patients with two slices could have been used, one with a PEEP of 5 cm of water and the other with a PEEP of 45 cm of water with quantitative analysis.[14] We made the choice of lung morphology for simplicity, avoiding quantitative analysis, but unfortunately it seems mandatory to perform such an analysis to quantify alveolar recruitment and hyperinflation without misclassification. In daily practice, avoiding this type of misclassification is probably easier than a trial setting, because response to the first recruitment manoeuvres is of high interest to characterise patients,[25] and other tools can be used at the patient's bedside, such as lung ultrasounds,[45] electrical impedance tomography,[46] and functional residual capacity measurement[47] to help physicians personalise the ventilation strategy. In this trial, assessment of lung morphology was done only on CT scans or chest x-rays before randomisation. Characterisation of lung morphology could not be modified according to haemodynamic tolerance or changes in PaO_2 and lung compliance following the first recruitment manoeuvres. Nevertheless, the present study's results highlight recommendations for better personalisation of mechanical ventilation in ARDS, given that higher mortality was observed in the misclassified patients when the applied ventilatory strategy was misaligned than in patients aligned with ventilatory strategy. Another limitation caused by simplification of the algorithm is related to the use of neuromuscular blockers and prone position in all patients with a PaO_2:FiO_2 ratio of less than 200 mm Hg, and not in those with a ratio of less than 150 mm Hg, as highlighted by the results of two large trials.[28,30] We made the choice of simplicity, sedation, neuromuscular blockers, and prone position for all included patients; we could have selected only patients with a PaO_2:FiO_2 ratio of less than 150 mm Hg, but we followed the Berlin definition. Nevertheless, prone position was not harmful for patients with a PaO_2:FiO_2 ratio of 100–200 mm Hg according to the findings of Taccone and colleagues.[41] For neuromuscular blockers, we have no evidence to support their use in patients with a PaO_2:FiO_2 ratio of 150–200 mm Hg. In our trial, neuromuscular blockers were stopped as soon as PaO_2:FiO_2 was higher than 200 mm Hg for 4 h.

The misclassified patients in this study support an old but unproven concept.[48] Although numerous physiological studies have shown that lung morphology is a strong predictor of response to PEEP setting or recruitment manoeuvres in terms of changes in oxygenation, recruited volume, or hyperinflation,[14,25,17] to our knowledge, this trial is the first to investigate mortality. When used for patients with non-focal ARDS, high PEEP and recruitment manoeuvres decrease mortality, as does high tidal volume, low PEEP, and prone position for those with focal ARDS. However, when mechanical ventilation is not aligned with phenotype (eg, high tidal volume and low PEEP for patients with non-focal ARDS or high PEEP and recruitment manoeuvres for patients with focal ARDS), mortality increases substantially. The distribution of lung

morphology (70% non-focal ARDS and 30% focal ARDS in different cohorts)[38,40] might explain the negative results reported in previous studies.[6,9,41,43] Moreover, mortality among these distinct ARDS phenotypes is different,[12,40] and an imbalance in the distribution of the two phenotypes at study inclusion might change the study results. The PROSEVA trial[30] investigating prone position does not contradict our study's hypothesis. The intervention group of the PROSEVA trial received low PEEP and prone position (the same as the personalised approach of the focal ARDS group in our trial) and the control group of the PROSEVA trial were equivalent to the non-focal ARDS patients of the intervention group in our trial. The PROSEVA trial showed that prone position saves lives, and the algorithm they used is similar to our own. Considering the mortality among misclassified patients in the present study, it seems worse to ventilate a patient with focal ARDS with high PEEP and low tidal volume (which was done for the patients with focal ARDS in the ART study)[9] than to ventilate patients with non-focal ARDS with low PEEP and prone position.[30] However, when a misaligned strategy is used, regardless of the ARDS phenotype, mortality increases significantly, as indicated by a significant interaction between the study groups and the misclassification of lung morphology.

One could question why misclassified patients in this study were only removed in the personalised group. In this study, the misclassified patients were not removed from the control group because they were not misaligned with the ventilator setting; ventilatory strategy was unrelated to lung morphology in the control group. Notwithstanding, when we removed misclassified patients from the control group for methodological reasons, the difference in 90 day mortality between the personalised and control group remained the same, but the difference was not significant in this case because of the smaller number of patients analysed. Our study is underpowered because the original sample size calculation over-estimated the mortality in the control group, and 5% of the recruited patients were excluded before analysis because of violation of eligibility criteria. When we designed our study, the most recent data available were from Papazian and colleagues,[28] a study in which mortality was 31% in the interventional group and 40% in the control group. We hypothesised that mortality in our study would be just higher than that in the interventional group. In hindsight, we should have recalculated the sample size before beginning the study, or we should have done an interim analysis to modify the number of patients to be enrolled. The reduction in mortality planned by Papazian and colleagues[28] was 15%, and a 10% decrease in mortality was observed; we therefore hypothesised that mortality would be located between these two values in our LIVE trial.

In conclusion, this trial's findings suggest that personalisation of mechanical ventilation based on lung morphology, without quantitative analysis of alveolar recruitment, did not improve 90 day mortality in patients with ARDS. These results might be explained by a high proportion of misclassified patients. When lung morphology is correctly assessed, mortality might decrease with personalised strategies in patients with moderate-to-severe ARDS. However, when personalised ventilation is misaligned with lung morphology, mortality increases substantially, suggesting a harmful effect of open-lung ventilation in patients without alveolar recruitment.

Contributors

J-MC and EF conceived of and designed the study. J-MC, MJ, J-YL, SJ, J-PQ, OL, MF, FG, PS, CI, BV, BS, TU, SL, FL, ML, NE, CD-F, HD, KA, AS, GC, LM, J-EB, AM, LB, J-JR, BP, J-MG, and EF acquired, analysed, or interpreted the data. JMC, MJ, EF, and BP created the first draft of the manuscript. J-MC, ML, J-YL, SJ, J-PQ, OL, MF, FG, PS, CI, BV, BS, TU, SL, FL, ML, NE, CD-F, HD, KA, AS, GC, LM, J-EB, AM, LB, J-JR, BP, J-MG, and EF provided crucial revision for important intellectual content and gave final approval of the manuscript. LB and BP were charged with data management. BP did the statistical analysis. J-MC and LB were tasked with administrative, technical, or material support. J-MC supervised the study.

Declaration of interests

J-MC reports personal fees and non-financial support from Drager, GE Healthcare, Sedana Medical, Baxter, and Amomed, personal fees from Fisher and Paykel Healthcare, Orion, Philips Medical, and Fresenius Medical Care, and non-financial support from LFB, and Bird Corporation, outside of the submitted work. EF reports personal fees from Edwards Lifesciences, Drager, General Electric Healthcare, Fresenius Kabi, Fisher and Paykel Healthcare, and Baxter, outside of the submitted work. MJ reports grants from the Conseil Régional d'Auvergne, the Agence Nationale de la Recherche, and the Direction Générale de l'Offre de Soins during the conduct of the study. KA reports personal fees from Baxter, LFB, Fresenius, and Fisher and Paykel Healthcare, outside of the submitted work. SJ reports personal fees from Drager, Fisher and Paykel Healthcare, Xenios, and Baxter, outside of the submitted work. ML reports grants from Pfizer, Amomed, Baxter, and MSD, outside of the submitted work. All other authors declare no competing interests.

Data sharing

Data collected for the study, including individual participant data, data dictionary defining each field in the set, and study protocol will be made available to others. Data will be communicated as de-identified participant data according to French law, and will be available after publication of the manuscript. Data are stored at the Direction de la Recherche Clinique, University Hospital of Clermont-Ferrand, Clermont-Ferrand, France. Data requests should be address to Dr Bruno Pereira (bpereira@chu-clermontferrand) who will send the data after receipt of a signed data access agreement.

References

1 Bellani G, Laffey JG, Pham T, et al. Epidemiology, patterns of care, and mortality for patients with acute respiratory distress syndrome in intensive care units in 50 countries. *JAMA* 2016; **315:** 788–13.

2 Herridge MS, Tansey CM, Matté A, et al. Functional disability 5 years after acute respiratory distress syndrome. *N Engl J Med* 2011; **364:** 1293–304.

3 Fan E, del Sorbo L, Goligher EC, et al. An official American Thoracic Society/European Society of Intensive Care Medicine/Society of Critical Care Medicine clinical practice guideline: mechanical ventilation in adult patients with acute respiratory distress syndrome. *Am J Respir Crit Care Med* 2017; **195:** 1253–63.

4 Amato MBP, Meade MO, Slutsky AS, et al. Driving pressure and survival in the acute respiratory distress syndrome. *N Engl J Med* 2015; **372:** 747–55.

5 Slutsky AS, Ranieri VM. Ventilator-induced lung injury. *N Engl J Med* 2013; **369:** 2126–36.

6 Brower RG, Lanken PN, MacIntyre N, et al. Higher versus lower positive end-expiratory pressures in patients with the acute respiratory distress syndrome. *N Engl J Med* 2004; **351:** 327–36.

7 Mercat A, Richard J-CM, Vielle B, et al. Positive end-expiratory pressure setting in adults with acute lung injury and acute respiratory distress syndrome: a randomized controlled trial. *JAMA* 2008; **299:** 646–55.
8 Meade MO, Cook DJ, Guyatt GH, et al. Ventilation strategy using low tidal volumes, recruitment maneuvers, and high positive end-expiratory pressure for acute lung injury and acute respiratory distress syndrome: a randomized controlled trial. *JAMA* 2008; **299:** 637–45.
9 Writing Group for the Alveolar Recruitment for Acute Respiratory Distress Syndrome Trial (ART) Investigators, Cavalcanti AB, Suzumura EA, et al. Effect of lung recruitment and titrated positive end-expiratory pressure (PEEP) vs low PEEP on mortality in patients with acute respiratory distress syndrome: a randomized clinical trial. *JAMA* 2017; **318:** 1335–45.
10 Sahetya SK, Brower RG. Lung recruitment and titrated PEEP in moderate to severe ARDS: is the door closing on the open lung? *JAMA* 2017; **318:** 1327.
11 Prescott HC, Calfee CS, Thompson BT, Angus DC, Liu VX. Toward smarter lumping and smarter splitting: rethinking strategies for sepsis and acute respiratory distress syndrome clinical trial design. *Am J Respir Crit Care Med* 2016; **194:** 147–55.
12 Calfee CS, Delucchi K, Parsons PE, et al. Subphenotypes in acute respiratory distress syndrome: latent class analysis of data from two randomised controlled trials. *Lancet Respir Med* 2014; **2:** 611–20.
13 Puybasset L, Cluzel P, Gusman P, Grenier P, Preteux F, Rouby J. Regional distribution of gas and tissue in acute respiratory distress syndrome. Consequences for lung morphology. *Int Care Med* 2000; **26:** 857–69.
14 Gattinoni L, Caironi P, Cressoni M, et al. Lung recruitment in patients with the acute respiratory distress syndrome. *N Engl J Med* 2006; **354:** 1775–86.
15 Jabaudon M, Blondonnet R, Lutz J, et al. Net alveolar fluid clearance is associated with lung morphology phenotypes in acute respiratory distress syndrome. *Anaesth Crit Care Pain Med* 2016; **35:** 81–86.
16 Laffey JG, Kavanagh BP. Negative trials in critical care: why most research is probably wrong. *Lancet Respir Med* 2018; **6:** 659–60.
17 Puybasset L, Gusman P, Muller JC, Cluzel P, Coriat P, Rouby JJ. Regional distribution of gas and tissue in acute respiratory distress syndrome. III. Consequences for the effects of positive end-expiratory pressure. CT Scan ARDS Study Group. Adult respiratory distress syndrome. *Int Care Med* 2000; **26:** 1215–27.
18 Delucchi K, Famous KR, Ware LB, et al. Stability of ARDS subphenotypes over time in two randomised controlled trials. *Thorax* 2018; **73:** 439–45.
19 Jabaudon M, Blondonnet R, Audard J, et al. Recent directions in personalised acute respiratory distress syndrome medicine. *Anaesth Crit Care Pain Med* 2018; **37:** 251–58.
20 Gattinoni L, Marini JJ, Pesenti A, Quintel M, Mancebo J, Brochard L. The 'baby lung' became an adult. *Int Care Med* 2016; **42:** 663–73.
21 Papazian L, Paladini M-H, Bregeon F, et al. Can the tomographic aspect characteristics of patients presenting with acute respiratory distress syndrome predict improvement in oxygenation-related response to the prone position? *Anesthesiology* 2002; **97:** 599–607.
22 Jabaudon M, Godet T, Futier E, et al. Rationale, study design and analysis plan of the lung imaging morphology for ventilator settings in acute respiratory distress syndrome study (LIVE study): study protocol for a randomised controlled trial. *Anaesth Crit Care Pain Med* 2017; **36:** 301–06.
23 Acute Respiratory Distress Syndrome Network, Brower RG, Matthay MA, et al. Ventilation with lower tidal volumes as compared with traditional tidal volumes for acute lung injury and the acute respiratory distress syndrome. *N Engl J Med* 2000; **342:** 1301–08.
24 ARDS Definition Task Force, Ranieri VM, Rubenfeld GD, et al. Acute respiratory distress syndrome: the Berlin Definition. *JAMA* **2012:** 2526–33.
25 Constantin J-M, Grasso S, Chanques G, et al. Lung morphology predicts response to recruitment maneuver in patients with acute respiratory distress syndrome. *Crit Care Med* 2010; **38:** 1108–17.
26 Constantin J-M, Cayot-Constantin S, Roszyk L, et al. Response to recruitment maneuver influences net alveolar fluid clearance in acute respiratory distress syndrome. *Anesthesiology* 2007; **106:** 944–51.
27 Chanques G, Jaber S, Barbotte E, et al. Validation of the French translated Richmond vigilance-agitation scale. *Annales Françaises d'Anesthésie et de Réanimation* 2006; **25:** 696–701 (in French).
28 Papazian L, Forel J-M, Gacouin A, et al. Neuromuscular blockers in early acute respiratory distress syndrome. *N Engl J Med* 2010; **363:** 1107–16.
29 Constantin J-M, Jaber S, Futier E, et al. Respiratory effects of different recruitment maneuvers in acute respiratory distress syndrome. *Crit Care* 2008; **12:** R50.
30 Guérin C, Reignier J, Richard J-C, et al. Prone positioning in severe acute respiratory distress syndrome. *N Engl J Med* 2013; **368:** 2159–68.
31 Boles J-M, Bion J, Connors A, et al. Weaning from mechanical ventilation. *Eur J Respir* 2007; **29:** 1033–56.
32 Lan KK, DeMets DL. Changing frequency of interim analysis in sequential monitoring. *Biometrics* 1989; **45:** 1017–20.
33 Meade MO, Cook DJ, Guyatt GH, et al. Ventilation strategy using low tidal volumes, recruitment maneuvers, and high positive end-expiratory pressure for acute lung injury and acute respiratory distress syndrome: a randomized controlled trial. *JAMA* 2008; **299:** 637–45.
34 Calfee CS, Delucchi KL, Sinha P, et al. Acute respiratory distress syndrome subphenotypes and differential response to simvastatin: secondary analysis of a randomised controlled trial. *Lancet Respir Med* 2018; **6:** 691–98.
35 Rouby J, Puybasset L, Nieszkowska A, Lu Q. Acute respiratory distress syndrome: lessons from computed tomography of the whole lung. *Crit Care Med* 2003; **31:** S285–95.
36 Jabaudon M, Hamroun N, Roszyk L, et al. Effects of a recruitment maneuver on plasma levels of soluble RAGE in patients with diffuse acute respiratory distress syndrome: a prospective randomized crossover study. *Int Care Med* 2015; **41:** 846–55.
37 Jabaudon M, Blondonnet R, Roszyk L, et al. Soluble forms and ligands of the receptor for advanced glycation end-products in patients with acute respiratory distress syndrome: an observational prospective study. *PLoS One* 2015; **10:** e0135857–12.
38 Jabaudon M, Blondonnet R, Roszyk L, et al. Soluble receptor for advanced glycation end-products predicts impaired alveolar fluid clearance in acute respiratory distress syndrome. *Am J Respir Crit Care Med* 2015; **192:** 191–99.
39 Jabaudon M, Blondonnet R, Lutz J, et al. Net alveolar fluid clearance is associated with lung morphology phenotypes in acute respiratory distress syndrome. *Anaesth Crit Care Pain Med* 2016; **35:** 81–86.
40 Mrozek S, Jabaudon M, Jaber S, et al. Elevated plasma levels of sRAGE are associated with nonfocal ct-based lung imaging in patients with ARDS: a prospective multicenter study. *Chest* 2016; **150:** 998–1007.
41 Taccone P, Pesenti A, Latini R, et al. Prone positioning in patients with moderate and severe acute respiratory distress syndrome: a randomized controlled trial. *JAMA* 2009; **302:** 1977–84.
42 Combes A, Hajage D, Capellier G, et al. Extracorporeal membrane oxygenation for severe acute respiratory distress syndrome. *N Engl J Med* 2018; **378:** 1965–75.
43 Mercat A, Richard JC, Vielle B, et al. Positive end-expiratory pressure setting in adults with acute lung injury and acute respiratory distress syndrome: a randomized controlled trial. *JAMA* 2008; **299:** 646.
44 Jabaudon M, Futier E, Roszyk L, et al. Soluble form of the receptor for advanced glycation end products is a marker of acute lung injury but not of severe sepsis in critically ill patients. *Crit Care Med* 2011; **39:** 480–88.
45 Bellani G, Rouby J-J, Constantin J-M, Pesenti A. Looking closer at acute respiratory distress syndrome: the role of advanced imaging techniques. *Curr Opin Crit Care* 2017; **23:** 30–37.
46 Constantin J-M, Perbet S, Delmas J, Futier E. Electrical impedance tomography: so close to touching the holy grail. *Crit Care* 2014; **18:** 164.
47 Dellamonica J, Lerolle N, Sargentini C, et al. PEEP-induced changes in lung volume in acute respiratory distress syndrome. Two methods to estimate alveolar recruitment. *Int Care Med* 2011; **37:** 1595–604.
48 Rouby J-J, Constantin J-M, de A Girardi CR, Zhang M, Lu Q. Mechanical ventilation in patients with acute respiratory distress syndrome. *Anesthesiology* 2004; **101:** 228–34.
