# Supplementary material for: Electrical impedance tomography–guided individualized ventilation strategy in patients with trauma-related and postoperative acute respiratory distress syndrome
Source: PLoS One. 2026 Aug 24;21(8):e0355609. doi: 10.1371/journal.pone.0355609 (PMC13502546; doi:10.1371/journal.pone.0355609)
Supplement: Supplementary file 4 [file pone.0355609.s012.pdf]

Reviewer Coments - PONE-D-26-05009R1

Comments to the Authors

This is a single-center randomized controlled trial evaluating an EIT-guided PEEP titration strategy in trauma- and postoperative-associated ARDS, a clinically relevant and underrepresented population. The authors should be commended for addressing an important and challenging aspect of mechanical ventilation—individualized PEEP selection in heterogeneous lung injury.

The manuscript has improved after revision, particularly in clarifying that the study was powered for physiologic rather than clinical endpoints and in tempering some of the initial overinterpretation. However, several important methodological and interpretive concerns remain, which limit the robustness of the conclusions.

Major Concerns

1. **Analysis population and internal inconsistency (critical issue)**

There is a significant inconsistency between the original manuscript and the response to reviewers regarding the analytical framework. The manuscript suggests that all randomized patients were included in the analysis, implying an intention-to-treat approach. However, the authors clarify in their response that analyses were performed only in patients with available follow-up data, without a full intention-to-treat framework.

This discrepancy is not trivial. It directly impacts the validity of the randomized comparison and must be explicitly clarified in the manuscript. The analytical population should be clearly defined (e.g., modified intention-to-treat, per-protocol), and the implications of excluding patients lost to follow-up must be discussed.

2. **Handling of longitudinal data and choice of statistical model**

The authors used repeated-measures general linear models despite the presence of missing data due to early transfer and potentially incomplete measurements. While the proportion of missing data is relatively small, the choice of model is suboptimal.

A linear mixed-effects model would be more appropriate, as it better handles unbalanced longitudinal data and reduces the risk of bias associated with complete-case or implicitly balanced approaches. The current justification for retaining repeated-measures GLM is not entirely convincing, particularly for a randomized longitudinal study.

3. **Handling of missing data and deaths**

The manuscript states that only observed physiologic data during Days 0–3 were analyzed, with no imputation after death or loss to follow-up. This approach assumes that missingness is non-informative, which may not be valid.

In critically ill populations, missing data due to death or clinical deterioration is often informative and may bias results toward survivors. The authors should more explicitly discuss this limitation and its potential impact on the physiologic comparisons.

4. **Intervention versus comparator asymmetry**

The intervention arm includes recruitment maneuvers and repeated dynamic PEEP titration guided by EIT, whereas the control group follows a lower-PEEP/FiO_2 table without recruitment maneuvers.

This represents more than a monitoring strategy comparison; it constitutes a bundled intervention. Therefore, the observed differences cannot be attributed solely to EIT guidance, but rather to the combined effect of recruitment maneuvers, higher PEEP exposure, and repeated titration. This should be explicitly acknowledged and discussed.

5. **Mechanistic limitation of the study (EIT data not analyzed)**

A central premise of the study is that EIT enables optimization of PEEP by balancing regional collapse and overdistension. However, the manuscript confirms that these EIT-derived variables were not systematically recorded for analysis.

This is a major limitation. Without quantitative EIT data, the study cannot substantiate the mechanistic pathway by which the intervention is presumed to act. Additionally, the absence of EIT acquisition in the control group prevents any direct comparison of regional ventilation patterns.

As a result, the manuscript demonstrates a physiologic effect (improved oxygenation) but provides limited mechanistic insight into how this effect was achieved.

6. **Subgroup analysis (pulmonary contusion)**

The subgroup analysis based on pulmonary contusion is interesting but remains underpowered and should be interpreted with caution. Although the authors now label it as exploratory, the analysis still receives substantial attention in the Results and Discussion. Given the sample size, these findings should be clearly framed as hypothesis-generating and should not influence the main conclusions.

Minor Concerns

- The abstract and discussion continue to emphasize clinical outcomes such as ICU length of stay and SOFA trajectory, despite the study not being powered for these endpoints. Interpretation should be further tempered.
- Data availability statements appear to have been inconsistent across versions and should be fully aligned with journal policy.
- Inclusion of the exact PEEP/FiO_2 table in supplementary material would improve reproducibility.
- Some sentences in the Discussion remain overly long and could be improved for clarity.

Overall Assessment

This is a clinically relevant randomized physiologic trial with a clear question and an appropriate general design. However, important methodological limitations—particularly regarding analysis population, handling of missing data, statistical modeling, and mechanistic interpretation—reduce confidence in the strength of the conclusions.

The study supports the feasibility of an EIT-guided PEEP strategy and suggests physiologic benefit in terms of oxygenation. However, it does not provide sufficient evidence to draw firm conclusions regarding mechanism or clinical impact.

Recommendation: Major Revision
