# Supplementary material for: Electrical impedance tomography–guided individualized ventilation strategy in patients with trauma-related and postoperative acute respiratory distress syndrome
Source: PLoS One. 2026 Aug 24;21(8):e0355609. doi: 10.1371/journal.pone.0355609 (PMC13502546; doi:10.1371/journal.pone.0355609)
Supplement: Supplementary file 5 [file pone.0355609.s014.pdf]

Reviewer Comments - PONE-D-26-05009

The authors should be congratulated for the considerable effort devoted to revising the manuscript. Compared with the previous version, the manuscript has substantially improved in methodological transparency, statistical rigor, and interpretation of the findings. The responses to the previous reviewers were comprehensive, and most of the major concerns have been satisfactorily addressed.

In particular, the clarification of the modified intention-to-treat population, replacement of repeated-measures ANOVA with linear mixed-effects models, improved discussion of missing data, and more cautious interpretation of secondary clinical outcomes significantly strengthen the scientific quality of the study. The manuscript now presents a much more transparent and statistically appropriate analysis.

The study addresses a clinically relevant question, namely whether an EIT-guided strategy for PEEP optimization can improve physiological outcomes in trauma- and postoperative-associated ARDS, a population that remains underrepresented in the literature. The randomized design and prospective data collection are important strengths.

Despite these improvements, several issues deserve further consideration before the manuscript is suitable for publication.

Major comments

1. The intervention should consistently be presented as a bundled ventilation strategy rather than an isolated EIT intervention.

Although the Discussion has been revised to acknowledge this issue, the manuscript occasionally continues to imply that the observed physiological benefits resulted directly from EIT guidance.

In reality, the intervention consisted of multiple simultaneous components, including recruitment maneuvers, decremental PEEP titration, repeated reassessment, individualized PEEP selection, and EIT monitoring. Consequently, the study compares two ventilation strategies rather than evaluating the independent effect of EIT itself.

The wording throughout the manuscript should consistently reflect this distinction. Expressions such as *"EIT-guided individualized ventilation strategy"* or *"EIT-guided PEEP optimization strategy"* would more accurately describe the intervention than suggesting an isolated effect of EIT.

2. Mechanistic interpretation remains limited.

The principal conceptual limitation of the study persists.

The proposed mechanism underlying the intervention is optimization of the balance between regional collapse and overdistension. However, quantitative EIT-derived variables were not systematically recorded, and EIT was not performed in the control group.

Therefore, although the study convincingly demonstrates improved oxygenation, it cannot establish whether this resulted from:

- improved regional recruitment,
- reduced overdistension,
- improved ventilation-perfusion matching,
- higher PEEP exposure,
- recruitment maneuvers,
- or the combined effects of the bundled intervention.

The current Discussion acknowledges this limitation but still occasionally implies mechanistic conclusions that are not directly supported by the available data.
I would recommend making the mechanistic interpretation even more conservative.

3. Discussion of mechanical power could be strengthened.

The authors appropriately report that mechanical power normalized to predicted body weight was modestly higher in the intervention group despite similar driving pressures.

However, the physiological interpretation remains relatively superficial.
Mechanical power is influenced by several interacting determinants, including tidal volume, respiratory rate, inspiratory flow, PEEP, and driving pressure. Because driving pressure remained unchanged while PEEP was consistently higher in the intervention

group, it is likely that the observed increase in MP/PBW primarily reflects greater PEEP exposure rather than worsening respiratory mechanics.

Expanding this discussion would improve the physiological interpretation and place the findings in the context of current concepts regarding mechanical power and ventilator-induced lung injury.

4. The Conclusions should remain slightly more conservative.

The Conclusions have improved substantially compared with previous versions. Nevertheless, I would recommend emphasizing that the study demonstrates the physiological feasibility of an EIT-guided ventilation strategy rather than establishing superiority of EIT as a monitoring modality.

Future studies incorporating quantitative EIT-derived measurements and parallel monitoring in both groups will be required to define the mechanistic pathways responsible for the observed physiological benefits.

Minor comments

1. Consider using consistent terminology throughout the manuscript. "EIT-guided individualized ventilation strategy" is scientifically more accurate than alternating between "EIT-guided ventilation" and "EIT-guided PEEP."
2. The Discussion could benefit from a more detailed integration of recent literature regarding regional lung stress, recruitability, and mechanical power, particularly work from Costa, Gattinoni, and colleagues.
3. Although not mandatory, a brief statement confirming assessment of assumptions underlying the linear mixed-effects models (residual distribution and model diagnostics) would further strengthen confidence in the statistical analyses.

Overall recommendation

This manuscript has improved substantially during the revision process and now represents a well-conducted randomized physiological study addressing an important clinical question. The remaining issues primarily concern interpretation rather than methodology.

I believe the manuscript is close to being suitable for publication after **minor revision**, mainly to further temper mechanistic conclusions and consistently frame the intervention as a bundled EIT-guided ventilation strategy rather than an evaluation of EIT technology alone.
