# Supplementary material for: Electrical impedance tomography–guided individualized ventilation strategy in patients with trauma-related and postoperative acute respiratory distress syndrome
Source: PLoS One. 2026 Aug 24;21(8):e0355609. doi: 10.1371/journal.pone.0355609 (PMC13502546; doi:10.1371/journal.pone.0355609)
Supplement: Supplementary file 6 [file pone.0355609.s016.pdf]

Reviewer coments - PONE-D-26-05009R3

I appreciate the opportunity to review the revised version of this manuscript entitled *"Electrical Impedance Tomography–Guided Individualized Ventilation Strategy in Patients with Trauma-Related and Postoperative Acute Respiratory Distress Syndrome."* The authors have been highly responsive throughout the review process, and the manuscript has improved substantially after multiple rounds of revision.

The major methodological concerns raised during the previous review rounds have been appropriately addressed. In particular, the authors have clarified the modified intention-to-treat analysis, adopted a more appropriate longitudinal statistical approach, substantially tempered the interpretation of subgroup analyses, and consistently acknowledge that the intervention represents a bundled EIT-guided ventilation strategy rather than an isolated assessment of EIT itself. These revisions considerably strengthen the scientific rigor and transparency of the study.

The conclusions are now appropriately balanced and consistent with the available data. The limitations are clearly acknowledged, particularly regarding the inability to attribute the observed physiological improvements to specific EIT-derived mechanisms due to the absence of quantitative regional ventilation analyses and the multicomponent nature of the intervention.

Although the study remains limited by its single-center design, modest sample size, physiologic primary endpoints, and lack of mechanistic EIT data, these limitations are transparently discussed and do not detract from the overall scientific value of the work. The manuscript provides clinically relevant information regarding individualized ventilatory management in a population that remains underrepresented in the literature.

I have only one minor editorial suggestion before publication:

- A final careful English language editing is recommended to improve readability and ensure consistency of terminology throughout the manuscript. Minor stylistic issues and occasional repetitive wording remain but can be corrected during copyediting.

Overall, I believe the authors have satisfactorily addressed the reviewers' concerns, and I have no further major comments.

Recommendation: Accept after Minor Editorial Revision.
