# Supplementary material for: Electrical impedance tomography–guided individualized ventilation strategy in patients with trauma-related and postoperative acute respiratory distress syndrome
Source: PLoS One. 2026 Aug 24;21(8):e0355609. doi: 10.1371/journal.pone.0355609 (PMC13502546; doi:10.1371/journal.pone.0355609)
Supplement: Supplementary file 7 [file pone.0355609.s002.docx]

**S1 Table. Lower PEEP/FiO₂ table used in the control group, adapted from the ARDS Network ALVEOLI trial.**

| **FiO₂** | **0.30** | **0.40** | **0.40** | **0.50** | **0.50** | **0.60** | **0.70** | **0.70** | **0.70** | **0.80** | **0.90** | **0.90** | **0.90** | **1.00** |
| --- | --- | --- | --- | --- | --- | --- | --- | --- | --- | --- | --- | --- | --- | --- |
| PEEP (cmH₂O) | 5 | 5 | 8 | 8 | 10 | 10 | 10 | 12 | 14 | 14 | 14 | 16 | 18 | 18–24 |

**FiO₂**, fraction of inspired oxygen; **PEEP**, positive end-expiratory pressure.
