# Supplementary material for: Electrical impedance tomography–guided individualized ventilation strategy in patients with trauma-related and postoperative acute respiratory distress syndrome
Source: PLoS One. 2026 Aug 24;21(8):e0355609. doi: 10.1371/journal.pone.0355609 (PMC13502546; doi:10.1371/journal.pone.0355609)
Supplement: Supplementary file 8 [file pone.0355609.s003.docx]

**S2 Table. Estimated marginal mean PEEP (cmH₂O) over time by pulmonary contusion status and study group.**

| **Time point** | **Pulmonary contusion: EIT** | **Pulmonary contusion: Control** | **p value** | **No pulmonary contusion: EIT** | **No pulmonary contusion: Control** | **p value** |
| --- | --- | --- | --- | --- | --- | --- |
| Day 0 | 6.10 (5.31 - 6.88) | 6.09 (5.34 - 6.83) | 0.988 | 5.81 (5.03 - 6.59) | 5.59 (4.72 - 6.46) | 0.709 |
| 1 hour | 9.71 (8.93 - 10.50) | 9.30 (8.56 - 10.05) | 0.456 | 9.29 (8.50 - 10.07) | 8.82 (7.96 - 9.69) | 0.437 |
| Day 1 | 8.38 (7.60 - 9.16) | 8.61 (7.86 - 9.36) | 0.679 | 8.71 (7.93 - 9.50) | 8.12 (7.25 - 8.99) | 0.316 |
| Day 2 | 9.52 (8.74 - 10.31) | 7.26 (6.51 - 8.01) | <0.001 | 8.67 (7.89 - 9.45) | 6.47 (5.60 - 7.34) | <0.001 |
| Day 3 | 9.05 (8.27 - 9.83) | 7.04 (6.30 - 7.79) | <0.001 | 7.86 (7.08 - 8.64) | 6.06 (5.19 - 6.93) | 0.003 |

Values are estimated marginal means (95% confidence intervals) derived from the linear mixed-effects model. P values are Sidak-adjusted for between-group comparisons at each time point within each subgroup.
