# Supplementary material for: Electrical impedance tomography–guided individualized ventilation strategy in patients with trauma-related and postoperative acute respiratory distress syndrome
Source: PLoS One. 2026 Aug 24;21(8):e0355609. doi: 10.1371/journal.pone.0355609 (PMC13502546; doi:10.1371/journal.pone.0355609)
Supplement: Supplementary file 9 [file pone.0355609.s004.docx]

**S3 Table. Fixed effects from exploratory subgroup linear mixed-effects models by pulmonary contusion status**

| **Outcome** | **Group** | **Time** | **Pulmonary contusion** | **Group × Time** | **Time × Pulmonary contusion** | **Group × Pulmonary contusion** | **Group × Time × Pulmonary contusion** |
| --- | --- | --- | --- | --- | --- | --- | --- |
| ΔMP | **F(1,78.1)=5.27**  **p=0.024** | **F(2,105.4)=10.64**  **p<0.001** | F(1,78.1)=0.14  p=0.703 | F(2,105.4)=0.10  p=0.903 | F(2,105.4)=0.92  p=0.399 | F(1,78.1)=1.41  p=0.238 | F(2,105.4)=0.34  p=0.707 |
| ΔCstat | **F(1,78.0)=7.51**  **p=0.008** | **F(2,118.5)=6.83**  **p=0.002** | F(1,78.0)=0.78  p=0.378 | F(2,118.5)=0.19  p=0.822 | F(2,118.5)=1.04  p=0.354 | F(1,78.0)=1.56  p=0.215 | F(2,118.5)=2.11  p=0.125 |
| PEEP | **F(1,77.2)=11.11**  **p=0.001** | **F(4,184.8)=66.11**  **p<0.001** | F(1,77.2)=3.77  p=0.056 | **F(4,184.8)=9.95**  **p<0.001** | F(4,184.8)=1.52  p=0.196 | F(1,77.2)=0.07  p=0.780 | F(4,184.8)=0.41  p=0.799 |

**ΔMP,** change in mechanical power; **ΔCstat**, change in static respiratory system compliance; **PEEP**, positive end-expiratory pressure.

Values are F statistics with denominator degrees of freedom and two-sided p values from linear mixed-effects models with fixed effects for treatment group, time, pulmonary contusion status, and interaction terms, with subject-specific random intercepts and autoregressive covariance structure.
