# Supplementary material for: Electrical impedance tomography–guided individualized ventilation strategy in patients with trauma-related and postoperative acute respiratory distress syndrome
Source: PLoS One. 2026 Aug 24;21(8):e0355609. doi: 10.1371/journal.pone.0355609 (PMC13502546; doi:10.1371/journal.pone.0355609)
Supplement: Supplementary file 10 [file pone.0355609.s005.docx]

**S4 Table. Sidak-adjusted pairwise comparisons for exploratory subgroup analyses**

| **Outcome** | **Subgroup** | **Time point** | **Mean difference**  **(EIT − Control)** | **p-value** |
| --- | --- | --- | --- | --- |
| ΔMP | Pulmonary contusion | Day 1 | 3.932 | **0.028** |
|  |  | Day 2 | 4.725 | **0.009** |
|  |  | Day 3 | 4.324 | **0.016** |
| ΔMP | No pulmonary contusion | Day 1 | 1.469 | 0.442 |
|  |  | Day 2 | 1.241 | 0.516 |
|  |  | Day 3 | 1.406 | 0.462 |
| ΔCstat | Pulmonary contusion | Day 1 | 3.694 | 0.165 |
|  |  | Day 2 | 0.495 | 0.852 |
|  |  | Day 3 | 2.781 | 0.295 |
| ΔCstat | No pulmonary contusion | Day 1 | 3.412 | 0.235 |
|  |  | Day 2 | 8.392 | **0.004** |
|  |  | Day 3 | 6.868 | **0.017** |

**ΔMP,** change in mechanical power; Δ**Cstat**, change in static respiratory system compliance. Values are from Sidak-adjusted pairwise comparisons.
