# Supplementary material for: Electrical impedance tomography–guided individualized ventilation strategy in patients with trauma-related and postoperative acute respiratory distress syndrome
Source: PLoS One. 2026 Aug 24;21(8):e0355609. doi: 10.1371/journal.pone.0355609 (PMC13502546; doi:10.1371/journal.pone.0355609)
Supplement: Supplementary file 12 [file pone.0355609.s008.docx]

# RESEARCH PROTOCOL

**Research protocol: part 1**

## 1. Project Summary

**Background:** Acute respiratory distress syndrome (ARDS) following trauma or major surgery is associated with high mortality, particularly in moderate-to-severe disease. Mechanical ventilation is essential for life support; however, inappropriate positive end-expiratory pressure (PEEP) may aggravate ventilator-induced lung injury through alveolar collapse or overdistension. Electrical impedance tomography (EIT) enables bedside assessment of regional ventilation and supports individualized PEEP titration based on lung recruitment and overdistension, with the potential to improve oxygenation and respiratory mechanics.

**Objectives:** To compare the effects of EIT-guided PEEP titration with a conventional ARDSNet low PEEP/FiO₂ strategy on oxygenation, respiratory system mechanics, and clinical outcomes in patients with trauma-related or postoperative moderate-to-severe ARDS.

**Methods:** This study will be conducted as a prospective, randomized, controlled clinical trial including adult patients with trauma-related or postoperative ARDS and a PaO₂/FiO₂ ratio ≤200 mmHg. Participants will be randomized to receive either EIT-guided PEEP titration following a recruitment maneuver or conventional PEEP settings according to the ARDSNet low PEEP/FiO₂ table. Data collection will include physiologic measurements of oxygenation and respiratory mechanics, Sequential Organ Failure Assessment (SOFA) scores, use of rescue therapies, complications, ventilator-free days, length of ICU and hospital stay, and 28-day mortality.

**Expected outcomes:** The study is expected to clarify whether EIT-guided PEEP titration improves oxygenation and respiratory system compliance without increasing ventilator-related complications, compared with conventional PEEP management in trauma-related and postoperative ARDS.

**2. General information**

- Protocol Title: Electrical Impedance Tomography–Guided Positive End-Expiratory Pressure Optimization in Patients With Trauma-Related and Postoperative Acute Respiratory Distress.
- Protocol identifying number and date: Trial registration: ClinicalTrials, NCT07313644. Registered 02 January 2026 – prospectively registered, <https://clinicaltrials.gov/search?cond=NCT07313644>.
- Name and title of the investigator who is responsible for conducting the research, and the address and telephone number of the research site, including responsibilities of each:

Nguyen Viet Minh

Department of Anesthesia and Critical Care, Hanoi Medical University, No.01, Ton That Tung Street, Kim Lien Ward, Hanoi, 11520, Vietnam.

Center of Anesthesia and Surgical Intensive Care, Viet Duc University Hospital, No.40, Trang Thi Street, Hoan Kiem Ward, Hanoi, 11000, Vietnam.

Tel: +(84) 918 292 528.

- Name and address of the clinical Institution involved in the research: Center of Anesthesia and Surgical Intensive Care, Viet Duc University Hospital, No.40, Trang Thi Street, Hoan Kiem Ward, Hanoi, 11000, Vietnam.

### 3. Rationale & background information

Acute respiratory distress syndrome (ARDS) remains a major cause of morbidity and mortality despite lung-protective ventilation, with variable outcomes reflecting marked heterogeneity in lung injury and respiratory mechanics (1). Although positive end-expiratory pressure (PEEP) is central to ARDS management, global PEEP/FiO₂ tables may inadequately represent regional lung behavior, limiting optimal balance between alveolar recruitment and overdistension (2).

Electrical impedance tomography (EIT) is a noninvasive bedside imaging technique that enables real-time assessment of regional ventilation and visualization of collapse and overdistension during PEEP titration (3). Physiological studies and randomized trials suggest that EIT-guided PEEP titration improves oxygenation and respiratory mechanics while reducing potentially injurious ventilatory load compared with conventional strategies (4-6).

However, most previous studies enrolled etiologically heterogeneous ARDS populations.

ARDS following trauma or major surgery represents a distinct clinical context characterized by pronounced regional heterogeneity due to dependent atelectasis, pleural effusions, pulmonary contusion, and altered chest wall mechanics (6-8). In this setting, global indices such as PaO₂/FiO₂ or static compliance may incompletely reflect regional recruitability, and PEEP/FiO₂ tables derived largely from medical ARDS cohorts may be suboptimal. Accordingly, this randomized controlled trial will compare EIT-guided PEEP titration with a conventional low PEEP/FiO₂ strategy in patients with moderate-to-severe trauma- and postoperative-associated ARDS, focusing on oxygenation, respiratory mechanics, and organ dysfunction.

## ****4. Study goals and objectives****

- **Study goals:** To evaluate whether individualized positive end-expiratory pressure (PEEP) titration guided by electrical impedance tomography (EIT) improves oxygenation and respiratory physiology compared with a conventional low PEEP/FiO₂ strategy in patients with moderate-to-severe trauma-related and postoperative acute respiratory distress syndrome (ARDS).
- **Primary objectives:** To compare the effects of EIT-guided PEEP titration and a conventional low PEEP/FiO₂ strategy on oxygenation and respiratory system mechanics, assessed by changes in the PaO₂/FiO₂ ratio and static respiratory system compliance during the first three days after intervention.
- **Secondary objectives:** To assess the effects of EIT-guided PEEP titration on ventilatory load and organ dysfunction, including mechanical power, driving pressure, and Sequential Organ Failure Assessment (SOFA) scores. To compare clinical outcomes between groups, including ventilator-free days at day 28, duration of mechanical ventilation, length of ICU stay, barotrauma, and 28-day mortality.
- **Exploratory objective:** To explore whether physiological responses to EIT-guided PEEP titration differ according to lung injury phenotype, particularly the presence or absence of pulmonary contusion.

**5. Study design**

This study will be conducted as a prospective, single-center, randomized controlled trial comparing electrical impedance tomography (EIT)–guided positive end-expiratory pressure (PEEP) titration with a conventional low PEEP/FiO₂ strategy in adult patients with moderate-to-severe trauma-related and postoperative acute respiratory distress syndrome (ARDS) receiving invasive mechanical ventilation.

**Inclusion criteria:**

- Age ≥18 years.
- Intubated patients with trauma-related or postoperative moderate-to-severe ARDS, defined according to the 2023 Global Definition of ARDS (9).

**Exclusion Criteria:**

- Age >90 years.
- Severe acute brain injury or stroke (Glasgow Coma Scale <8).
- Untreated or clinically unstable pneumothorax or pneumomediastinum, defined as ongoing air leak or radiographic progression despite appropriate drainage.
- End-stage disease or expected survival <7 days.
- Conditions requiring prolonged mechanical ventilation unrelated to ARDS (e.g., Guillain–Barré syndrome or cervical spinal cord injury).
- Prior use of advanced respiratory therapies, including extracorporeal membrane oxygenation, inhaled nitric oxide, prone positioning, or high-frequency ventilation.
- Pregnancy or breastfeeding.
- Contraindications to EIT monitoring, including implanted electrical devices, skin lesions at electrode sites, or known allergy to electrode materials.
- Refusal of informed consent or concurrent enrollment in another interventional trial.

Patients with chest trauma, including pulmonary contusion or stabilized pneumothorax after appropriate drainage, were eligible for inclusion.

**Randomization**:

Patients will be randomized in a 1:1 ratio to the EIT-guided PEEP group or the control group using computer-generated block randomization with concealed allocation. Due to the nature of the intervention, treating clinicians will not be blinded to group assignment.

**Study Duration and Follow-up:**

Physiologic outcomes will be assessed from Day 0 to Day 3 after randomization. Clinical outcomes will be followed until ICU discharge or death, with mortality and ventilator-free days assessed at day 28.

### 6. Methodology

**Intervention**

*EIT group*

Electrical impedance tomography (EIT) will be performed using a commercial system (Enlight 2100, Timpel, São Paulo, Brazil). A 32-electrode belt will be positioned circumferentially around the thorax at the 4th–6th intercostal space using a diaphragm-based positioning method (10). Patients will be deeply sedated (Richmond Agitation–Sedation Scale ≤ −3) without spontaneous breathing, and airway secretions will be adequately cleared prior to the procedure.

A recruitment maneuver will be performed using pressure-controlled ventilation with an inspiratory pressure of 15 cmH₂O above PEEP and PEEP set at 24 cmH₂O (maximum airway pressure ≤40 cmH₂O) for 30 seconds at FiO₂ 1.0. A decremental PEEP titration will then be conducted, starting from 24 cmH₂O and decreasing in 2 cmH₂O steps to a minimum of 6 cmH₂O or until SpO₂ ≤80%, with each PEEP level maintained for 30 seconds.

Optimal PEEP will be defined as the level corresponding to the intersection of cumulative regional collapse and overdistension curves derived from EIT analysis, reflecting a balance between alveolar recruitment and overdistension (3). PEEP selection will be based on the manufacturer’s standardized EIT algorithm and independently confirmed by two investigators blinded to clinical outcomes. Following titration, a recruitment maneuver will be repeated, and PEEP will be set at the EIT-determined level for a minimum of 12 hours.

**Fig 1. Determination of PEEP Using EIT-Based Analysis**. The recommended PEEP was identified at the intersection of the cumulative collapse (blue) and overdistension (white) curves, representing the point at which the estimated proportions of atelectasis and overinflation were balanced. In this example, the recommended PEEP was 12 cmH₂O.

PEEP reassessment using the same protocol will be recommended once daily for up to three days. If repeat titration is contraindicated (e.g., hemodynamic instability or barotrauma), a single titration on Day 0 will be permitted. PEEP reduction (2 cmH₂O every 4–8 hours) will be considered when PaO₂/FiO₂ exceeds 200 with FiO₂ <0.5; clinical deterioration will prompt return to the EIT-derived PEEP with repeat recruitment if indicated.

PEEP titration will be discontinued in cases of sustained mean arterial pressure decrease >20 mmHg, SpO₂ <88%, or new-onset arrhythmias, after which previous ventilator settings will be restored.

***Control group:*** Sedation management will be identical to that of the EIT group. PEEP will be set according to the lower ARDS Network PEEP/FiO₂ table. Routine recruitment maneuvers will not be performed.

| **FiO_2_** | **0.3** | **0.4** | **0.4** | **0.5** | **0.5** | **0.6** | **0.7** | **0.7** | **0.7** | **0.8** | **0.9** | **0.9** | **0.9** | **1.0** |
| --- | --- | --- | --- | --- | --- | --- | --- | --- | --- | --- | --- | --- | --- | --- |
| **PEEP** | 5 | 5 | 8 | 8 | 10 | 10 | 10 | 12 | 14 | 14 | 14 | 16 | 18 | 18-24 |

Adjunctive Therapies**:** Prone positioning will be considered for patients with PaO₂/FiO₂ <150 mmHg while receiving PEEP >10 cmH₂O and FiO₂ >0.6, and may be maintained for up to 16 hours per day (11). Extracorporeal membrane oxygenation (ECMO) will be considered as rescue therapy if oxygenation targets are not achieved.

**Other respiratory therapies:** Maintenance ventilation will be delivered using volume-controlled ventilation as part of a lung-protective strategy. Pressure-controlled ventilation will be used transiently for standardized recruitment and EIT-guided titration maneuvers. Ventilator settings will target a plateau pressure ≤30 cmH₂O, tidal volume of 4–6 mL/kg predicted body weight, and respiratory rate ≤35 breaths/min, adjusted to maintain arterial pH ≥7.30. The inspiratory-to-expiratory ratio will be set between 1:1 and 1:3. Oxygenation targets will be PaO₂ 55–80 mmHg or SpO₂ 88–95%.

**Measurements and data collection:**

**Primary outcome:** Oxygenation and respiratory system mechanics will be assessed using the PaO₂/FiO₂ ratio and static respiratory system compliance.

Secondary outcomes will include in-hospital and 28-day mortality, ventilator-free days to discharge, ICU length of stay, occurrence of barotrauma, use of rescue therapies (including prone positioning and ECMO), and Sequential Organ Failure Assessment (SOFA) scores.

**Fig 2.** Flow chart of the study

## ****7. Safety considerations****

Participant safety will be a primary consideration throughout the study. Particular attention will be given to the risk of barotrauma (including pneumothorax and subcutaneous emphysema) and hemodynamic instability associated with PEEP titration. Patients will be continuously monitored for ventilatory and hemodynamic parameters during recruitment maneuvers and PEEP adjustments, and predefined safety thresholds will prompt immediate intervention or discontinuation of the procedure when necessary.

All adverse events will be systematically recorded and followed according to institutional standards. Data confidentiality will be strictly maintained, and all study procedures will be conducted as part of routine intensive care management to minimize additional physical burden to participants.

## ****8. Follow-up****

Physiologic follow-up will be performed during the early study period after randomization. Respiratory variables, including ventilator settings and respiratory system mechanics (PaO₂/FiO₂ ratio, static compliance, driving pressure, plateau pressure, and mechanical power), will be recorded at baseline and daily from Day 0 through Day 3. Arterial blood gas analyses will be obtained at the same time points to assess oxygenation.

Clinical follow-up will be conducted throughout the ICU stay. Hemodynamic instability, occurrence of barotrauma, and use of rescue therapies will be actively monitored and documented according to standard intensive care practice. Organ dysfunction will be assessed using Sequential Organ Failure Assessment (SOFA) scores. Clinical outcomes, including ventilator-free days and mortality, will be evaluated up to day 28.

### 9. Data management and statistical analysis

Data will be collected using standardized case report forms and entered into a secure, password-protected database with coded identifiers to ensure confidentiality. Data quality will be ensured through regular monitoring and verification.

Categorical variables will be summarized as counts and percentages, and continuous variables as mean ± standard deviation or median (interquartile range), as appropriate. Between-group comparisons will be performed using standard parametric or nonparametric tests. Longitudinal changes will be analyzed using repeated-measures models, with prespecified subgroup analyses according to pulmonary contusion status. Statistical analyses will be conducted using SPSS version 27.0, with two-sided p < 0.05 considered statistically significant.

## ****10. Quality assurance****

Quality assurance will be conducted in accordance with Good Clinical Practice (GCP) guidelines. Source data and case report forms will be reviewed regularly to ensure adherence to the study protocol. Data management procedures will include validation and periodic audits to maintain data accuracy and integrity.

## ****11. Expected outcomes of the study****

This study is expected to clarify whether individualized positive end-expiratory pressure (PEEP) titration guided by electrical impedance tomography improves oxygenation and respiratory system mechanics compared with a conventional low PEEP/FiO₂ strategy in patients with moderate-to-severe trauma-related and postoperative acute respiratory distress syndrome. The results are anticipated to provide detailed physiological information on changes in oxygenation, compliance, driving pressure, and mechanical power, as well as short-term clinical outcomes such as ventilator-free days and organ dysfunction. Findings from this study will contribute to improved understanding of individualized PEEP optimization in trauma- and postoperative-associated ARDS.

## ****12. Dissemination of results and publication policy****

The results of this study will be disseminated through publication in peer-reviewed scientific journals and presentation at relevant national and international conferences. Findings may also be shared internally with participating clinicians for educational and clinical discussion purposes.

Manuscript preparation will be led by the principal investigator, with contributions from co-investigators according to their roles in the study. All contributors and funding sources will be appropriately acknowledged. Study results will be reported transparently and in accordance with international publication standards, regardless of the direction or significance of the findings.

## ****13. Duration of the project****

The overall duration of the project is expected to be approximately 24 months. This period will include protocol development, ethical approval, staff training, and database preparation, followed by patient recruitment and follow-up to day 28 or hospital discharge. Data cleaning, statistical analysis, and interpretation will be conducted after completion of follow-up, with the final phase dedicated to manuscript preparation and dissemination of results.

## ****14. Problems anticipated****

Potential challenges may include slower-than-expected patient recruitment due to strict eligibility criteria and occasional technical limitations related to electrical impedance tomography (EIT) monitoring. To mitigate these issues, patient screening will be conducted consistently in the surgical intensive care unit, and EIT measurements will be performed by trained staff according to standardized procedures. Close in-hospital follow-up is expected to minimize missing data and ensure completeness of outcome assessment within the study period.

### 15. Ethics

The protocol was approved by the Institutional Review Board of Hanoi Medical University (No. 1879/GCN-HMUIRB). Written informed consent was obtained from patients or their legal surrogates. The study complied with the Declaration of Helsinki and was reported in accordance with CONSORT guidelines. The trial was registered at ClinicalTrials.gov (Identifier: NCT07313644).

**16. Informed consent**

Written informed consent will be obtained from each prospective participant or, when the participant lacks decision-making capacity, from a legally authorized representative (LAR), in accordance with the requirements of the Institutional Review Board of Hanoi Medical University. Given the critical condition of most eligible patients, consent is expected to be obtained primarily from LARs.

The investigator or trained study staff obtaining consent will provide clear and comprehensive information regarding the study objectives, procedures, potential risks, and anticipated benefits, and will answer any questions raised by the participant or LAR. Any new information that may affect willingness to continue participation will be communicated in a timely manner. The consent process will be conducted using language understandable to the LAR and in a manner that minimizes coercion or undue influence. Written informed consent will be obtained before initiation of any study-related procedures. The signed and dated consent form will document the LAR’s understanding and agreement to the participant’s involvement in the study. The principal investigator will be responsible for ensuring full compliance with all institutional and ethical requirements for informed consent.

**Research protocol: part 2**

## ****Budget****

This study will not require external funding. All study procedures, clinical investigations, and data management activities will be conducted using existing hospital resources and routine clinical care infrastructure. No financial support is requested from any funding agency.

## ****Other support for the project****

No external financial or material support has been received or is anticipated for this project. All study activities will be carried out using resources available at the participating hospital.

## ****Collaboration with other scientists or research institutions****

Faculty members from the Department of Anesthesia and Critical Care, Hanoi Medical University, in collaboration with intensivists from the Center of Anesthesia and Surgical Intensive Care, Viet Duc University Hospital, will jointly oversee the study. Their responsibilities will include study design, staff training, monitoring of protocol implementation, and supervision of data collection and analysis. This collaboration will ensure methodological rigor, adherence to Good Clinical Practice (GCP), and integration of academic and clinical expertise.

## ****Links to other projects****

This study will be conducted as an independent research project and will not be linked to any other ongoing or completed studies.

### Information of investigators

**Nguyen Viet Minh^1,2^, Trinh Van Dong^1,2^, Nguyen Dang Thu^3^, Nguyen Thi Huyen^4^, Luu Quang Thuy^1,2*^**

^1^ Department of Anesthesia and Critical Care, Hanoi Medical University, Hanoi, Vietnam

^2^ Center of Anesthesia and Surgical Intensive Care, Viet Duc University Hospital, Hanoi, Vietnam

^3^ Department of Anesthesiology, Military Hospital 103, Vietnam Military Medical University, Hanoi, Vietnam

^4^ Institute of Diagnostic and Interventional Radiology, Bach Mai Hospital, Hanoi, Vietnam

## ****Other research activities of the investigators****

The Principal Investigator will not be involved in other research projects during the conduct of this study. Accordingly, no conflict related to time commitment or funding is anticipated.

**Financing and insurance**

**This study will not receive external financial support and will not require special insurance arrangements. Participants will be covered by standard hospital patient insurance policies in accordance with institutional regulation.**

**Reference**

1.	Fan E, Brodie D, Slutsky AS. Acute Respiratory Distress Syndrome: Advances in Diagnosis and Treatment. Jama. 2018;319(7):698–710. doi: 10.1001/jama.2017.21907. PubMed PMID: 29466596.

2.	Amato MB, Meade MO, Slutsky AS, Brochard L, Costa EL, Schoenfeld DA, et al. Driving pressure and survival in the acute respiratory distress syndrome. N Engl J Med. 2015;372(8):747–55. doi: 10.1056/NEJMsa1410639. PubMed PMID: 25693014.

3.	Frerichs I, Amato MB, van Kaam AH, Tingay DG, Zhao Z, Grychtol B, et al. Chest electrical impedance tomography examination, data analysis, terminology, clinical use and recommendations: consensus statement of the TRanslational EIT developmeNt stuDy group. Thorax. 2017;72(1):83–93. Epub 20160905. doi: 10.1136/thoraxjnl-2016-208357. PubMed PMID: 27596161; PubMed Central PMCID: PMC5329047.

4.	Costa EL, Borges JB, Melo A, Suarez-Sipmann F, Toufen C, Jr., Bohm SH, et al. Bedside estimation of recruitable alveolar collapse and hyperdistension by electrical impedance tomography. Intensive Care Med. 2009;35(6):1132–7. Epub 20090303. doi: 10.1007/s00134-009-1447-y. PubMed PMID: 19255741.

5.	Zhao Z, Chang MY, Chang MY, Gow CH, Zhang JH, Hsu YL, et al. Positive end-expiratory pressure titration with electrical impedance tomography and pressure-volume curve in severe acute respiratory distress syndrome. Ann Intensive Care. 2019;9(1):7. Epub 20190117. doi: 10.1186/s13613-019-0484-0. PubMed PMID: 30656479; PubMed Central PMCID: PMC6336593.

6.	He H, Chi Y, Yang Y, Yuan S, Long Y, Zhao P, et al. Early individualized positive end-expiratory pressure guided by electrical impedance tomography in acute respiratory distress syndrome: a randomized controlled clinical trial. Crit Care. 2021;25(1):230. Epub 20210630. doi: 10.1186/s13054-021-03645-y. PubMed PMID: 34193224; PubMed Central PMCID: PMC8243615.

7.	Pape HC, Remmers D, Rice J, Ebisch M, Krettek C, Tscherne H. Appraisal of early evaluation of blunt chest trauma: development of a standardized scoring system for initial clinical decision making. J Trauma. 2000;49(3):496–504. doi: 10.1097/00005373-200009000-00018. PubMed PMID: 11003329.

8.	Cohn SM, Dubose JJ. Pulmonary contusion: an update on recent advances in clinical management. World J Surg. 2010;34(8):1959–70. doi: 10.1007/s00268-010-0599-9. PubMed PMID: 20407767.

9.	Matthay MA, Arabi Y, Arroliga AC, Bernard G, Bersten AD, Brochard LJ, et al. A New Global Definition of Acute Respiratory Distress Syndrome. Am J Respir Crit Care Med. 2024;209(1):37–47. doi: 10.1164/rccm.202303-0558WS. PubMed PMID: 37487152; PubMed Central PMCID: PMC10870872.

10.	Yue C, He H, Su L, Wang J, Yuan S, Long Y, et al. A novel method for diaphragm-based electrode belt position of electrical impedance tomography by ultrasound. J Intensive Care. 2023;11(1):41. Epub 20230925. doi: 10.1186/s40560-023-00691-2. PubMed PMID: 37749637; PubMed Central PMCID: PMC10518967.

11.	Guérin C, Reignier J, Richard JC, Beuret P, Gacouin A, Boulain T, et al. Prone positioning in severe acute respiratory distress syndrome. N Engl J Med. 2013;368(23):2159–68. Epub 20130520. doi: 10.1056/NEJMoa1214103. PubMed PMID: 23688302.
