# Supplementary material for: Electrical impedance tomography–guided individualized ventilation strategy in patients with trauma-related and postoperative acute respiratory distress syndrome
Source: PLoS One. 2026 Aug 24;21(8):e0355609. doi: 10.1371/journal.pone.0355609 (PMC13502546; doi:10.1371/journal.pone.0355609)
Supplement: Supplementary file 13 [file pone.0355609.s013.docx]

Manuscript Number: [PONE-D-26-05009] - [EMID:b4caae6824179d2a]

Response to Reviewers and Editors

Roberta Ribeiro De Santis Santiago, M.D., Ph.D., R.R.T.
Academic Editor
*PLOS One*

# We thank you for the opportunity to revise and resubmit our manuscript entitled “*Electrical Impedance Tomography–Guided Positive End-Expiratory Pressure Optimization in Patients With Trauma-Related and Postoperative Acute Respiratory Distress Syndrome*.” We sincerely appreciate the time and effort invested by the editor and reviewers in evaluating our work. Their insightful comments and constructive suggestions have been invaluable in improving the clarity and rigor of the manuscript. We have carefully addressed all comments and revised the manuscript accordingly. Changes are highlighted in yellow in the revised version, and detailed, point-by-point responses are provided below, with our replies indicated in blue.

**Response to Academic Editor**

# We thank the Academic Editor for the careful and thoughtful evaluation of our manuscript. We appreciate the constructive and insightful comments, which have helped improve the clarity, methodological transparency, and interpretation of our study. We have revised the manuscript accordingly and addressed all concerns in detail below.

- **The study addresses the role of individualized PEEP titration guided by EIT in patients with ARDS from a subgroup of trauma and post-surgical subjects. The authors conducted an RCT enrolling a subgroup of patients with ARDS, with the opportunity to provide a better understanding of the role of individualized mechanical ventilation. Overall, based on the reviewers' comments, it lacks novelty, and they raised major concerns and suggested revisions to clarify and provide more detail in the methodology and data analysis sections.**

We appreciate this important comment. We have revised the Introduction and Discussion to more clearly position the novelty of our study. Specifically, our trial focuses on a relatively underrepresented ARDS population-trauma- and postoperative-associated ARDS-characterized by distinct patterns of regional heterogeneity and recruitability compared with predominantly medical ARDS cohorts. We have emphasized that the study contributes to the field by:

- Evaluating EIT-guided PEEP titration in a clinically distinct ARDS population.
- Exploring phenotype-specific physiologic responses (pulmonary contusion vs non-contusion).
- Integrating mechanical power and compliance responses into the interpretation of individualized ventilation strategies.

We have clarified these points to better contextualize the contribution of the study.

- **I am adding the following comments to the reviewer's suggestions/concerns: The main results compare PaO2/FiO2, PEEP, Cstat, driving pressure, and mechanical power. Therefore, it is very important to address these measurements. In the data collection and outcomes section, it is unclear whether the driving pressure calculation accounted for the total PEEP in its formula (driving pressure = Plateau pressure - PEEP total) (page 7, line 179). It would be important to clarify this.**

We thank the reviewer for this important comment. We have carefully addressed these points in detail in our responses to the reviewer below and have revised the manuscript accordingly.

- **Regarding the Cstat, I would suggest reassessing Cstat normalized by predicted body weight (per se based on height and sex) to strengthen the comparison between groups (Costa ELV et al. Am J Respir Crit Care Med. 2021 PMID: 33784486).**

We thank the Editor for this insightful suggestion. We agree that normalization of respiratory mechanics to predicted body weight (PBW) may improve comparability across patients of different body size.

We note that the referenced study by Costa et al. primarily emphasizes mechanical power normalized to PBW (MP/PBW) as a physiologically integrative metric that incorporates multiple components of ventilatory load. In line with this approach, our study already included MP normalized to PBW (MP/PBW) as a prespecified secondary physiologic outcome.

As shown in our Results, MP/PBW decreased over time in both groups, with modestly higher values in the EIT group but without a significant group-by-time interaction, indicating similar temporal trends between groups. These findings are presented and interpreted in both the Results and Discussion sections.

By contrast, static compliance (Cstat) was reported in absolute terms. In trauma- and postoperative-associated ARDS, Cstat reflects the combined effects of lung and chest wall mechanics, which may vary substantially due to injury-related and postoperative factors. We therefore considered absolute Cstat more appropriate for physiologic interpretation in this population.

We have cited the suggested reference (Costa ELV et al.) and clarified this rationale in the revised manuscript.

- **In the discussion session (page 14, lines 398-400), the authors mentioned the potential benefit of EIT for assessing regional ventilation distribution, but the manuscript lacks exploration of regional ventilation and the mechanics of the study population. Do you have EIT data acquired in the control group for comparison?**

We thank the Editor for this important comment. In the present study, EIT was used as a bedside clinical tool to guide individualized PEEP titration in the intervention group rather than as a modality for comprehensive assessment of regional ventilation mechanics.

EIT monitoring was not performed in the control group because the study was designed to compare an EIT-guided strategy with a standard-of-care approach, without introducing additional monitoring or co-interventions in the control arm. This approach was intended to preserve the pragmatic nature of the comparator and to ensure that observed differences were primarily attributable to the EIT-guided PEEP titration strategy.

As a result, direct comparison of regional ventilation patterns between groups was not feasible. We have clarified this point in the revised manuscript and explicitly acknowledged it as a limitation.

- **The manuscript lacks information about overdistension/collapse findings, which are the alleged advantages of the EIT approach.**

We thank the Editor for this important comment. We agree that assessment of regional overdistension and collapse represents a key advantage of EIT. In the present study, EIT-derived information on regional collapse and overdistension was used in real time to guide individualized PEEP selection in the intervention group. However, these quantitative data were not systematically recorded for subsequent analysis, as the primary aim of the study was to evaluate physiologic outcomes associated with an EIT-guided PEEP strategy rather than to perform a detailed mechanistic analysis of regional ventilation patterns.

As a result, we were unable to provide formal analyses of overdistension and collapse across time or between groups. This limitation has now been explicitly acknowledged in the revised manuscript.

- **Have the authors identified any EIT information that could help phenotyping patients as advocated in the manuscript?**

We thank the Editor for this insightful comment. We agree that EIT has the potential to support phenotyping based on regional ventilation patterns.

In the present study, however, EIT was not used for systematic phenotypic classification, as its primary role was to guide individualized PEEP titration in the intervention group. Quantitative EIT-derived indices were not collected in a manner that would allow formal phenotyping analysis.

Instead, we performed prespecified subgroup analyses based on pulmonary contusion, representing a clinically relevant phenotype of traumatic lung injury. This approach was chosen to explore whether underlying patterns of lung injury, particularly structural disruption associated with pulmonary contusion, may influence physiologic responses to PEEP.

We have clarified in the revised manuscript that these subgroup analyses are exploratory and hypothesis-generating, and that further studies incorporating detailed EIT-derived regional metrics may help refine phenotype-based ventilation strategies.

**Response to Reviewer #1**

We thank the reviewer for the thorough and constructive evaluation of our manuscript. We appreciate the recognition of the methodological rigor and clinical relevance of our study, as well as the insightful comments provided. These suggestions have been invaluable in improving the clarity, consistency, and interpretation of the manuscript. We have carefully revised the manuscript to address all comments, as detailed below.

**Major Points**

1. **Sample Size & Outcome Interpretation: Clearly state that the sample size was powered for a physiological (PaO2/FiO2) endpoint, not for detecting differences in clinical outcomes (e.g., mortality, VFDs). This is crucial for correctly interpreting the negative findings on these secondary endpoints throughout the study.**

We thank the reviewer for this important comment. We have revised the manuscript to clarify that the sample size was based on PaO₂/FiO₂ as the primary physiologic endpoint and that the study was not designed or sufficiently powered to detect differences in clinical outcomes. We also revised the Discussion to present clinical outcomes as secondary exploratory findings rather than definitive evidence of clinical benefit . Revision made:

Methods (Statistical Analysis): “Sample size estimation was based on a two-sided comparison of independent means (α = 0.05, power = 90%) using Day 1 PaO₂/FiO₂, with allowance for 20% attrition.”

Discussion: ‘’Although 28-day mortality did not differ between groups, duration of ventilation and ICU length of stay were shorter in the EIT group. These outcomes were secondary and exploratory. The study was designed and powered for physiologic rather than clinical endpoints; therefore, these findings should not be interpreted as definitive evidence of clinical benefit. In addition, duration of ventilation and ICU length of stay may be influenced by non-respiratory factors, including sedation practices, weaning strategies, surgical recovery, and ICU discharge decisions. Further studies specifically powered for clinical outcomes are required to determine whether the observed physiologic improvements translate into reproducible clinical benefit.’’

2. **EIT Protocol & Adherence: Clarify the stability criterion during the decremental PEEP trial (30 seconds per step). Briefly justify or cite evidence that this duration is sufficient for stable alveolar recruitment/derecruitment in this population. Please provide actual protocol adherence data: How many patients in the EIT group underwent repeat titration on Days 1, 2, and 3? This is essential for interpreting the sustained physiological effects.**

We thank the reviewer for this important comment. We have clarified the stability criterion used during the decremental PEEP trial and provided justification with appropriate references. Each PEEP level was maintained for 30 seconds to allow stabilization of regional ventilation signals before proceeding to the next step. This approach is consistent with previously described EIT-guided decremental PEEP protocols, in which PEEP is reduced in stepwise increments with short stabilization periods (e.g., 30 seconds per step) during EIT-based titration (*van der Zee P, Somhorst P, Endeman H, Gommers D. Electrical Impedance Tomography for Positive End-Expiratory Pressure Titration in COVID-19-related Acute Respiratory Distress Syndrome. Am J Respir Crit Care Med. 2020;202(2):280-284. doi:10.1164/rccm.202003-0816LE*).

Regarding protocol adherence, all patients in the intervention group underwent initial EIT-guided PEEP titration on Day 0. In the EIT group, 43 patients were randomized, and one patient was transferred early and was not further followed, leaving 42 patients with available follow-up. Repeat EIT-guided PEEP titration was planned on Days 1–3 according to the study protocol, with predefined safety criteria. In the present study, no patients met these criteria during the protocolized period. Accordingly, repeat titration was performed in all 42 patients with available follow-up on Day 1, Day 2, and Day 3. This reflects full protocol adherence during the study period. We have clarified these points in the revised Methods and Results sections.

Methods:

“A decremental PEEP titration followed, starting at 24 cmH₂O and decreasing in 2 cmH₂O steps to a minimum of 6 cmH₂O or until SpO₂ ≤80% with each level maintained for 30 seconds. This duration was chosen to allow stabilization of regional ventilation signals, consistent with prior EIT-guided decremental PEEP protocols (26).”

“EIT-guided PEEP reassessment was performed according to protocol on Days 1–3 in all patients with available follow-up in the EIT group.”

Results:

“Protocol adherence was high in the intervention arm: all analyzed patients underwent initial EIT-guided PEEP titration on Day 0, and repeat titration was performed on Days 1–3 in all patients with available follow-up.”

3. **Control Group Strategy: Specify the exact "lower ARDS Network PEEP/FiO2 table" used (e.g., cite the source publication). Consider including the table in the supplement for reproducibility. The term "conventional low PEEP/FiO2 strategy" is vague. And why in the control group, RMs were not performed?**

We thank the reviewer for this important comment. We have clarified the control group strategy to improve precision and reproducibility. As described in the revised Methods, PEEP in the control group was applied according to the lower-PEEP/FiO_2_ strategy of the ARDS Network ALVEOLI trial, using predefined PEEP–FiO₂ combinations, and the corresponding reference has been added (*Brower RG, Lanken PN, MacIntyre N, Matthay MA, Morris A, Ancukiewicz M, et al. Higher versus lower positive end-expiratory pressures in patients with the acute respiratory distress syndrome. N Engl J Med. 2004;351(4):327–36. doi: 10.1056/NEJMoa032193. PubMed PMID: 15269312*). To avoid ambiguity, we have replaced the term “conventional low PEEP/FiO₂ strategy” with this more specific description.

Routine recruitment maneuvers were not performed to reflect a standard comparator strategy and to avoid additional co-interventions beyond the protocolized PEEP approach. This approach is consistent with current ESICM guidelines, which suggest against the routine use of brief high-pressure recruitment maneuvers (≥35 cmH₂O for <1 minute) in patients with ARDS, as no mortality benefit has been demonstrated (Grasselli G, Calfee CS, Camporota L, et al. ESICM guidelines on acute respiratory distress syndrome: definition, phenotyping and respiratory support strategies. *Intensive Care Med*. 2023;49(7):727-759. doi:10.1007/s00134-023-07050-7).

Revision made (Methods):

“PEEP was applied according to the lower-PEEP/FiO_2_ strategy of the ARDS Network ALVEOLI trial, using predefined PEEP–FiO₂ combinations (27).”

“This approach is consistent with contemporary ESICM guidelines, which suggest against the routine use of brief high-pressure recruitment maneuvers in ARDS due to the absence of demonstrated mortality benefit (28).”

4. **Subgroup Analysis Presentation: The analysis by pulmonary contusion status is interesting but must be explicitly labeled as exploratory and hypothesis-generating due to its post-hoc nature and limited sample size. For the key finding of higher ΔMP in contusion patients with EIT, provide the corresponding PEEP levels in this subgroup. This will help distinguish the effect of the EIT method from the effect of simply applying higher PEEP in this specific pathology.**

We thank the reviewer for this insightful comment. We agree that the subgroup analysis should be interpreted with appropriate caution. We have clarified throughout the manuscript that the analyses stratified by pulmonary contusion status were prespecified but exploratory and not powered for definitive subgroup comparisons. Accordingly, these findings are now consistently described as hypothesis-generating. Regarding the observation of higher ΔMP in the EIT group, we agree that PEEP is a key determinant of mechanical power. As reported in the Results, PEEP levels were higher in the EIT group at later time points across both subgroups, without a significant interaction with pulmonary contusion status.

In the Discussion, we have clarified that higher MP/PBW in the EIT group was not accompanied by increases in driving pressure or plateau pressure, suggesting that the observed differences primarily reflect ventilatory settings, particularly higher PEEP. We have also emphasized that this pattern was consistent across subgroups.

**Revision made**

**Results:**

“In corresponding analyses, PEEP decreased over time (time effect: F(3,76)=19.20, p < 0.001) and was higher in the EIT group compared with the control group (F(1,78)=11.70, p = 0.001), with a significant time-by-treatment interaction (F(3,76)=9.95, p < 0.001). Neither the time-by-contusion interaction (p = 0.129) nor the three-way interaction among time, pulmonary contusion, and treatment group (p = 0.628) was significant, indicating consistent temporal patterns across subgroups. Sidak-adjusted comparisons showed no between-group differences at early time points, whereas higher PEEP levels were observed in the EIT group at later time points (Days 2–3) in both pulmonary contusion and non-contusion subgroups (all p < 0.01). This pattern is further illustrated by subgroup-specific estimated marginal means (S3 Table).”

**Discussion:**

“These differences paralleled the higher PEEP levels observed in the EIT group. This suggests that increased MP may be reflect ventilatory settings rather than intrinsic differences in lung injury.”

“In this context, higher PEEP may contribute to increased mechanical load without proportional recruitment (5). However, the absence of a significant interaction indicates that this pattern was not confined to this subgroup and was consistent across phenotypes.”

“These findings should be interpreted as exploratory and hypothesis-generating. The study was not powered for definitive subgroup comparisons.”

5. **Discussion & Interpretation: Specify the unique pathophysiology of trauma/postoperative ARDS (e.g., chest wall mechanics, lower inflammatory burden) and how it might influence responses to EIT-guided PEEP compared to sepsis-related ARDS.**

We thank the reviewer for this insightful comment. We agree that trauma- and postoperative-associated ARDS may differ from sepsis-related ARDS in important pathophysiologic aspects that could influence responses to ventilatory strategies. In the revised manuscript, we have clarified that this population is often characterized by heterogeneous lung injury, including focal contusion or atelectasis, as well as altered chest wall mechanics related to trauma or surgery. These features may influence respiratory system mechanics and lung recruitability. Compared with sepsis-related ARDS, which is often associated with more diffuse inflammatory lung injury, trauma- and postoperative-associated ARDS may exhibit greater regional heterogeneity and variable recruitability.

We have also expanded the Discussion to highlight how these differences may influence responses to EIT-guided PEEP. In particular, we emphasize that in heterogeneous lung injury, the effects of PEEP may vary across regions, and EIT may provide additional value by enabling region-specific assessment of collapse and overdistension, which may not be captured by conventional global indices.

Revision made (Discussion):

“Unlike prior EIT studies in predominantly sepsis- or pneumonia-related ARDS (7-9, 31), our cohort comprised trauma- and postoperative-associated ARDS, characterized by marked regional heterogeneity due to dependent atelectasis, pleural effusions, and altered chest wall mechanics.

Lung injury in this population is often heterogeneous, with focal contusion or atelectasis. In pulmonary contusion, alveolar hemorrhage, interstitial edema, and ventilation–perfusion mismatch contribute to reduced compliance and impaired gas exchange (32).

These factors may influence lung recruitability and the response to PEEP. Compared with sepsis-related ARDS, which is often characterized by more diffuse inflammatory lung injury, trauma- and postoperative-associated ARDS may exhibit greater regional heterogeneity and variable recruitability. The effects of PEEP are known to depend on lung morphology, recruitability, and overall respiratory system mechanics (34, 35). In heterogeneous lung injury, the same level of PEEP may result in recruitment in some regions while causing overdistension in others.

In this context, EIT may be particularly useful, as it provides bedside assessment of regional ventilation and supports individualized PEEP titration based on the balance between collapse and overdistension (36).”

**Minor Points**

1. **Provide a clear definition for "Weaning success" in the Methods or Table S2 footnote.**
2. **Some sentences, particularly in the Discussion, are too long.**
3. **Double-check that all abbreviations are defined at first use.**

We thank the reviewer for these helpful comments. We have revised the manuscript accordingly by (1) providing a clear definition of weaning success in the Methods section, (2) improving readability through shortening long sentences, particularly in the Discussion, and (3) ensuring that all abbreviations are defined at first use and used consistently throughout the manuscript.

**Revision made (Methods):**

“Weaning success was defined as sustained liberation from mechanical ventilation for at least 48 hours without the need for reintubation.”

**Response to Reviewer #2**

We thank the reviewer for the careful and constructive evaluation of our manuscript. We sincerely appreciate the reviewer’s insightful comments, which have been highly valuable in improving the clarity, methodological rigor, and overall quality of the manuscript. We have revised the manuscript accordingly, as detailed below.

1. **Data availability statement does not match information provided in how the data may be accessed.**

We thank the reviewer for identifying this inconsistency. We have revised the Data Availability statement to ensure consistency between the stated restrictions and the process for accessing the data.

**Revision made:**

‘The data are not publicly available due to restrictions imposed by the Institutional Review Board (IRB) of Hanoi Medical University to protect participant confidentiality. Deidentified data are available upon reasonable request through the IRB of Hanoi Medical University (email: daihocyhn@hmu.edu.vn), subject to ethical approval and institutional data access policies. The data are securely stored at Hanoi Medical University in accordance with institutional policies to ensure long-term availability.’’

2. **What is the purpose of the "Etiologic Classification" in the methods section? I'm not sure this is the best place for this.**

We thank the reviewer for this insightful comment. We agree that the placement of etiologic classification in the Methods section may suggest a prespecified subgroup hypothesis. To improve clarity, we have revised the manuscript by de-emphasizing this description in the Methods and presenting it within the Results section as part of the subgroup analyses. Specifically, etiologic classification is now described in the context of exploratory subgroup analyses, and we have clarified that these analyses are hypothesis-generating and not powered for definitive conclusions.

**Revision made ( Subgroup analyses):**

‘’Pulmonary contusion was defined on admission chest computed tomography. For exploratory subgroup analyses, patients were classified as pulmonary contusion or non-contusion, with the latter including pneumonia, extrapulmonary sepsis, and transfusion-related acute lung injury. This classification aimed to distinguish traumatic parenchymal injury from non-traumatic inflammatory etiologies. Subgroup analyses according to pulmonary contusion status were exploratory and hypothesis-generating, reflecting the limited sample size and lack of statistical power for subgroup comparisons.’’

3. **For the primary outcomes - which day is the primary outcome, and is the primary outcome the absolute level or the change from baseline, or is it all of the days - e.g. mean over 3 days, or change over three days.**

We thank the reviewer for this important comment. We have clarified that the co-primary physiologic outcomes were PaO₂/FiO₂ and Cstat assessed longitudinally over the predefined study period (Days 0–3). The primary analyses evaluated repeated measurements over time to assess temporal trajectories and between-group differences, rather than focusing on a single time point or a mean value over time. Changes from baseline were analyzed as supportive measures to aid interpretation but were not defined as primary endpoints.

**Revision made (Methods):**

“The co-primary physiologic outcomes were PaO₂/FiO₂ and Cstat, assessed longitudinally over the predefined study period (Days 0–3). Analyses focused on repeated measurements over time to evaluate temporal trajectories and between-group differences. Changes from baseline were analyzed as supportive measures to aid interpretation of physiologic responses.”

4. **For the longitudinal models - please add details about e.g. Pillai's trace, and the adjustment for multiple comparisons made.**

We thank the reviewer for this important comment. We have added further details to the Statistical Analysis section to clarify the longitudinal modeling approach. Specifically, repeated-measures general linear models were used, with Pillai’s trace applied for multivariate testing, and Sidak-adjusted post hoc comparisons were performed to account for multiple comparisons.

**Revision made (Statistical Analysis):**

“Longitudinal changes were analyzed using repeated-measures general linear models (time as within-subject factor, group as between-subject factor), with Pillai’s trace and Sidak-adjusted post hoc comparisons.”

5. **How was missing data dealt with in the non-repeated measures analyses?**

We thank the reviewer for this important question. Non-repeated measures analyses were conducted using complete-case analysis in patients with available follow-up data. Four randomized patients were lost to follow-up due to early inter-hospital transfer and were not included in these analyses. No imputation was performed for missing data.

**Revision made (Statistical Analysis):**

“Non-repeated measures analyses were conducted using complete-case analysis in patients with available follow-up data. No imputation was performed for missing data.”

6. **Did you assess sphericity in your repeated measures glm?**

We thank the reviewer for this important comment. Yes, sphericity was assessed as part of the repeated-measures analysis. We have clarified this in the Statistical Analysis section and specified that the Greenhouse–Geisser correction was applied when the sphericity assumption was violated.

**Revision made (Statistical Analysis):**

“Sphericity was assessed and corrected using Greenhouse–Geisser when violated.”

7. **Usual implementations of repeated measures anova drop subjects who are missing even one measure - is this what you did? A more flexible approach would be to use a linear mixed model.**

We thank the reviewer for this important methodological comment. Longitudinal analyses were performed using repeated-measures general linear models. Four randomized patients were lost to follow-up due to early inter-hospital transfer and were not included in the final analyses. We agree that linear mixed models provide a more flexible approach for handling incomplete longitudinal data. However, given the small proportion of missing data in our study (4/86 patients) and the high completeness of repeated measurements, we retained the repeated-measures GLM approach. We have clarified the analyzed population and missing-data handling in the revised manuscript to improve transparency.

**Revision made (Statistical Analysis):**

“Non-repeated measures analyses were conducted using complete-case analysis in patients with available follow-up data. No imputation was performed for missing data.”

8. **Was an intention to treat approach used?**

We thank the reviewer for this important comment. Because four randomized patients were lost to follow-up after early inter-hospital transfer and were not further followed, analyses were performed in patients with available follow-up data rather than under a full intention-to-treat framework. We have revised the manuscript to clarify the analyzed population and to ensure transparency in reporting.

**Revision made (Statistical Analysis):**

“Non-repeated measures analyses were conducted using complete-case analysis in patients with available follow-up data.”

9. **Remove p values from Table S1.**

We thank the reviewer for this suggestion. While statistical testing of baseline characteristics is not essential in randomized trials, we retained p values in Table 1 to provide a complete descriptive comparison between groups. These values are presented for transparency only and were not used for inferential interpretation or to inform any analytical decisions.

10. **How were deaths dealt with in the analyses? This is the most important point raised here. Please carefully consider how death was dealt with in the primary analyses, as well as the clinical comparisons.**

We thank the reviewer for raising this important point. We have clarified the handling of deaths according to outcome type to ensure transparent interpretation of the analyses. For the co-primary physiologic outcomes, analyses were based on observed measurements obtained during the predefined study period (Days 0–3). Missing physiologic data were handled within the repeated-measures framework without imputation, and no imputation was performed after death or loss to follow-up. Four randomized patients were lost to follow-up due to early inter-hospital transfer and were not included in the final outcome analyses. For clinical outcomes, deaths were retained as observed events in the corresponding comparisons, including 28-day mortality and ventilator-free days. We have revised the manuscript to clarify the analyzed population, missing-data handling, and the interpretation of outcomes.

**Revision made (Statistical Analysis):**

“For the co-primary physiologic analyses, only observed measurements obtained during Days 0–3 were analyzed, and no imputation was performed after death or loss to follow-up.”

“Non-repeated measures analyses were conducted using complete-case analysis in patients with available follow-up data. No imputation was performed for missing data.”

11. **Ensure that the measures presented in Figure 4 are described in the methods section.**

We thank the reviewer for this helpful comment. We have revised the Methods section to ensure that the ventilatory variables presented in Figure 4 are explicitly described in the Data Collection and Outcomes section.

**Revision made (Methods):**

“At each predefined study time point, ventilatory variables recorded included applied PEEP, Pplat, Pdriv, Cstat, and MP.”
