# Supplementary material for: Electrical impedance tomography–guided individualized ventilation strategy in patients with trauma-related and postoperative acute respiratory distress syndrome
Source: PLoS One. 2026 Aug 24;21(8):e0355609. doi: 10.1371/journal.pone.0355609 (PMC13502546; doi:10.1371/journal.pone.0355609)
Supplement: Supplementary file 14 [file pone.0355609.s015.docx]

Manuscript Number: [[PONE-D-26-05009R1] - [EMID:f34553fd3da164d7]

Response to Reviewers and Editors

Roberta Ribeiro De Santis Santiago, M.D., Ph.D., R.R.T.
Academic Editor
*PLOS One*

# We thank you for the opportunity to revise and resubmit our manuscript entitled “*Electrical Impedance Tomography–Guided Positive End-Expiratory Pressure Optimization in Patients With Trauma-Related and Postoperative Acute Respiratory Distress Syndrome*.” We sincerely appreciate the time and effort invested by the editor and reviewers in evaluating our work. Their insightful comments and constructive suggestions have been invaluable in improving the clarity and rigor of the manuscript. We have carefully addressed all comments and revised the manuscript accordingly. A revised version with tracked changes is provided, and detailed point-by-point responses are presented below, with our replies indicated in blue.

**Response to Reviewer #1**

We thank the reviewer for the thorough and constructive evaluation of our manuscript. We appreciate the recognition of the methodological rigor and clinical relevance of our study, as well as the insightful comments provided. These suggestions have been invaluable in improving the clarity, consistency, and interpretation of the manuscript. We have carefully revised the manuscript to address all comments, as detailed below.

**The author provided detailed & appropriate responses to all of my comments.
The only minor concern is the Figures, which have room for improvement. For example, resolution of the Figure 1, which would be better if it is a original screenshot.**

We sincerely thank the reviewer for the positive assessment of our revisions and for the helpful final suggestion regarding the figures. In response, we have improved the overall figure quality and replaced **Figure 1** with a higher-resolution original system screenshot/export to enhance image clarity and readability. The figure formatting and legend have also been refined to improve presentation quality in the revised manuscript. We appreciate this valuable recommendation.

**Response to Reviewer #3**

We sincerely thank the reviewer for the careful reassessment of our manuscript and for the thoughtful, constructive comments. We greatly appreciate the recognition of the clinical relevance of this trial and of the improvements made in the revised version, particularly regarding clarification that the study was powered for physiologic rather than clinical endpoints and the more cautious interpretation of secondary outcomes. We also appreciate the reviewer’s additional methodological and interpretive concerns, which have been highly valuable in further strengthening the manuscript. In response, we have undertaken substantial additional revisions, including clarification of the modified intention-to-treat analysis population, replacement of repeated-measures analyses with linear mixed-effects modeling for longitudinal outcomes, expanded discussion of missing data and mechanistic limitations, clearer acknowledgment that the intervention represented a bundled EIT-guided PEEP optimization strategy, and further tempering of subgroup and clinical outcome interpretations. Detailed point-by-point responses to each comment are provided below.

**Major Concerns**

**1. Analysis population and internal inconsistency (critical issue)**

**There is a significant inconsistency between the original manuscript and the response to reviewers regarding the analytical framework. The manuscript suggests that all randomized patients were included in the analysis, implying an intention-to-treat approach. However, the authors clarify in their response that analyses were performed only in patients with available follow-up data, without a full intention-to-treat framework.**

**This discrepancy is not trivial. It directly impacts the validity of the randomized comparison and must be explicitly clarified in the manuscript. The analytical population should be clearly defined (e.g., modified intention-to-treat, per-protocol), and the implications of excluding patients lost to follow-up must be discussed.**

We thank the reviewer for this important comment. We agree that the analytical population required clearer and fully consistent reporting. We have revised the manuscript to explicitly define the primary analysis population as a modified intention-to-treat (mITT) population, consisting of all randomized patients who received the allocated intervention and had at least one post-randomization outcome assessment available. This clarification has now been incorporated consistently in the Abstract, Methods, Results, Fig 2, and Limitations section.

Four randomized patients were transferred early to other hospitals before analyzable follow-up data could be collected and therefore had no post-randomization longitudinal physiologic measurements available for inclusion in the prespecified repeated-measures analyses. These patients were excluded from the mITT population because the co-primary physiologic outcomes required post-randomization follow-up assessments.

We also agree that the implications of these exclusions should be discussed. Accordingly, we have added a limitation acknowledging that exclusion of these early-transferred patients means the analysis was not a full intention-to-treat analysis and may introduce limited attrition bias, although the number of excluded patients was small (4/86) and reasonably balanced between groups (1 in EIT, 3 in control).

**Revision made:**

**Abstract (Methods):** “Four patients were transferred early before analyzable follow-up data were collected; therefore, the modified intention-to-treat population included 42 patients in the EIT group and 40 in the control group.”

**Methods (Statistical analysis):** “The primary analysis population was a modified intention-to-treat population, defined as all randomized patients who received the allocated intervention and had at least one post-randomization outcome assessment available. Patients transferred before collection of analyzable follow-up data were excluded.”

**Results:** “Accordingly, the modified intention-to-treat population included 82 patients, comprising 42 patients in the EIT group and 40 patients in the control group.”

**Limitations:** “Because four randomized patients were transferred early and had no analyzable post-randomization follow-up data, the analysis was not a full intention-to-treat analysis. Although the proportion of excluded patients was small (4/86), this may introduce limited attrition bias and should be considered when interpreting the randomized comparison.”

**2. Handling of longitudinal data and choice of statistical model**

**The authors used repeated-measures general linear models despite the presence**

**of missing data due to early transfer and potentially incomplete measurements. While**

**the proportion of missing data is relatively small, the choice of model is suboptimal.A linear mixed-effects model would be more appropriate, as it better handles unbalanced longitudinal data and reduces the risk of bias associated with complete-case or implicitly balanced approaches. The current justification for retaining repeated-measures GLM is not entirely convincing, particularly for a randomized longitudinal study.**

We thank the reviewer for this important methodological comment and agree that a linear mixed-effects modeling approach is more appropriate for longitudinal outcomes in the presence of unbalanced repeated measurements. In response, we have revised the statistical analysis and replaced the previous repeated-measures general linear model analyses with linear mixed-effects models (LMMs) for all longitudinal outcomes.

The revised models included fixed effects for treatment group, time, and the group-by-time interaction, with subject-specific random intercepts and an autoregressive covariance structure to account for within-subject correlation across repeated measurements. This approach uses all available observations without requiring complete data at every time point and is more robust to missing follow-up measurements due to early transfer, death, or clinical deterioration.

We have updated the Methods, Results, figure legends, and Supporting Information tables accordingly. Importantly, the principal findings were materially unchanged after reanalysis, with sustained between-group differences in oxygenation favoring the EIT group and no major changes in the interpretation of respiratory mechanics or clinical outcomes. This consistency strengthens the robustness of the reported physiologic effects.

**Revision made:**

**Methods (Statistical analysis):** “Longitudinal outcomes during Days 0–3 were analyzed using linear mixed-effects models with fixed effects for treatment group, time, and the group-by-time interaction, with subject-specific random intercepts and an autoregressive covariance structure. This approach used all available repeated measurements while accounting for within-subject correlation.”

**Results:** All repeated longitudinal analyses for PaO₂/FiO₂, ΔPaO₂/FiO₂, PEEP, Cstat, Pplat, Pdriv, MP/PBW, and exploratory subgroup outcomes were reanalyzed using linear mixed-effects models, and corresponding statistics have been updated throughout the manuscript.

**Figure legends (Figs 3–5):** “Longitudinal changes were analyzed using linear mixed-effects models with fixed effects for group, time, and group-by-time interaction, with Sidak-adjusted post hoc comparisons where applicable.”

**Supporting Information:** Updated subgroup model outputs and pairwise comparisons are now presented in S2–S4 Tables based on linear mixed-effects models.

**3. Handling of missing data and deaths**

**The manuscript states that only observed physiologic data during Days 0–3 were analyzed, with no imputation after death or loss to follow-up. This approach assumes that missingness is non-informative, which may not be valid.**

**In critically ill populations, missing data due to death or clinical deterioration is often informative and may bias results toward survivors. The authors should more explicitly discuss this limitation and its potential impact on the physiologic comparisons.**

We thank the reviewer for this important comment. We agree that missing physiologic data due to death, clinical deterioration, or early transfer in critically ill populations may be informative rather than completely random, and that this limitation should be explicitly acknowledged.

In response, we have revised the manuscript to clarify that longitudinal outcomes during Days 0–3 were analyzed using linear mixed-effects models based on all available observed repeated measurements, with no imputation performed after death, transfer, or loss to follow-up. This approach allows use of incomplete repeated data while accounting for within-subject correlation, but does not eliminate the possibility of informative missingness.

We have therefore expanded the Limitations section to explicitly state that missing physiologic data after early transfer, death, or clinical deterioration may not have been random, and that longitudinal comparisons during Days 0–3 should be interpreted cautiously. We appreciate the reviewer’s comment, which has improved the transparency and interpretation of the study findings.

Revision made:

**Methods (Statistical analysis):**

“Longitudinal outcomes during Days 0–3 were analyzed using linear mixed-effects models with fixed effects for treatment group, time, and the group-by-time interaction, with subject-specific random intercepts and an autoregressive covariance structure. This approach used all available repeated measurements while accounting for within-subject correlation.”

“No imputation was performed after death, transfer, or loss to follow-up.”

**Discussion (Limitations):**

“Missing physiologic data after early transfer, death, or clinical deterioration may not have been random; therefore, longitudinal comparisons during Days 0–3 should be interpreted cautiously. No post-event imputation was performed.”

**4. Intervention versus comparator asymmetry**

**The intervention arm includes recruitment maneuvers and repeated dynamic PEEP titration guided by EIT, whereas the control group follows a lower-PEEP/FiO₂ table without recruitment maneuvers.**

**This represents more than a monitoring strategy comparison; it constitutes a bundled intervention. Therefore, the observed differences cannot be attributed solely to EIT guidance, but rather to the combined effect of recruitment maneuvers, higher PEEP exposure, and repeated titration. This should be explicitly acknowledged and discussed.**

We thank the reviewer for this important comment. We agree that the intervention arm represented more than a monitoring-only comparison. The EIT group received a protocolized strategy that incorporated recruitment maneuvers, decremental titration, repeated reassessment, and individualized PEEP adjustment, whereas the control group received a lower-PEEP/FiO₂ table strategy without routine recruitment maneuvers. We therefore agree that the observed differences cannot be attributed solely to EIT monitoring itself.

In response, we have revised the manuscript to consistently describe the intervention as an EIT-guided PEEP optimization strategy rather than EIT guidance alone. We have also strengthened the Discussion and Clinical Implications sections to explicitly acknowledge that the observed physiologic effects likely reflect the combined influence of recruitment maneuvers, higher PEEP exposure at later time points, repeated titration, and regional monitoring-informed adjustment. Accordingly, the trial should be interpreted as a comparison of two ventilation strategies rather than an isolated test of bedside monitoring technology.

We appreciate the reviewer’s comment, which has improved the conceptual framing and interpretation of the study.

**Revision made:**

**Discussion:** “In this randomized controlled trial of moderate-to-severe trauma- and postoperative-associated ARDS, an EIT-guided PEEP optimization strategy was associated with greater improvement in oxygenation than a lower-PEEP/FiO₂ strategy. Measurements were obtained after post-titration stabilization. Therefore, sustained differences were more likely related to individualized PEEP selection than to transient recruitment effects alone.”

**Clinical implications and future directions:** “Our findings support the feasibility of an EIT-guided bundled PEEP optimization strategy and suggest a physiologic oxygenation benefit in trauma- and postoperative-associated ARDS.”

**Conclusions:** “These findings suggest the physiological feasibility of an EIT-guided individualized PEEP optimization strategy, although larger multicenter studies are needed to determine effects on patient-centered outcomes.”

**5. Mechanistic limitation of the study (EIT data not analyzed)**

**A central premise of the study is that EIT enables optimization of PEEP by balancing regional collapse and overdistension. However, the manuscript confirms that these EIT-derived variables were not systematically recorded for analysis.**

**This is a major limitation. Without quantitative EIT data, the study cannot substantiate the mechanistic pathway by which the intervention is presumed to act. Additionally, the absence of EIT acquisition in the control group prevents any direct comparison of regional ventilation patterns.As a result, the manuscript demonstrates a physiologic effect (improved oxygenation) but provides limited mechanistic insight into how this effect was achieved.**

We thank the reviewer for this important comment. We agree that the absence of systematically recorded quantitative EIT-derived variables limits mechanistic interpretation. Although EIT was used clinically to guide individualized PEEP titration in the intervention group, quantitative EIT-derived indices were not systematically collected for formal analysis. In addition, EIT was not performed in the control group; therefore, direct comparison of regional ventilation patterns between groups was not possible.

We have revised the manuscript to explicitly acknowledge this limitation and to temper mechanistic interpretation. The revised text clarifies that the study demonstrates a physiologic effect, particularly improved oxygenation, but cannot definitively establish the mechanistic pathway by which this effect was achieved.

**Revision made:**

**Discussion:**
“Sustained improvements over time may be consistent with improved regional ventilation–perfusion matching; however, this mechanism was not directly confirmed because quantitative EIT-derived indices were not systematically collected.”

**Discussion:**
“Although quantitative indices of collapse and overdistension were not analyzed, PEEP titration was guided by real-time EIT assessment of regional ventilation.”

**Limitations:**
“Finally, mechanistic inferences were limited because EIT was not performed in the control group and quantitative EIT-derived indices were not systematically collected.”

**6. Subgroup analysis (pulmonary contusion)**

**The subgroup analysis based on pulmonary contusion is interesting but remains underpowered and should be interpreted with caution. Although the authors now label it as exploratory, the analysis still receives substantial attention in the Results and Discussion. Given the sample size, these findings should be clearly framed as hypothesis-generating and should not influence the main conclusions.**

We thank the reviewer for this important comment. We agree that the pulmonary contusion subgroup analyses were underpowered for definitive subgroup inference and should be interpreted cautiously. These analyses were intended to explore whether traumatic lung injury phenotype might modify physiologic responses, but not to provide confirmatory evidence of treatment-effect heterogeneity.

In response, we have further revised the manuscript to consistently frame all pulmonary contusion subgroup findings as exploratory and hypothesis-generating. We have reduced emphasis on these results in both the Results and Discussion, clarified that formal interaction testing did not identify consistent subgroup-specific treatment effects, and ensured that the main Conclusions do not rely on subgroup findings.

We appreciate the reviewer’s comment, which has helped improve the balance and interpretation of the manuscript.

**Revision made:**

**Results (Subgroup analyses):**

“Subgroup analyses according to pulmonary contusion status were exploratory and hypothesis-generating because the study was not powered for subgroup comparisons.”

“Neither the time-by-contusion interaction nor the group-by-time-by-contusion interaction was significant, indicating similar temporal PEEP patterns across subgroups.”

“ΔMP showed a broadly similar pattern across subgroups.”

“In contrast, selected later differences in ΔCstat were observed among patients without pulmonary contusion, whereas no clear between-group differences were identified in patients with pulmonary contusion.”

**Discussion:**

“Exploratory subgroup analyses did not demonstrate consistent treatment-effect heterogeneity, although selected later improvements in Cstat were observed among patients without pulmonary contusion.”

“Although exploratory and underpowered, our findings suggested possible differences in selected physiologic responses according to pulmonary contusion status; however, formal interaction testing did not identify consistent subgroup-specific temporal effects (40, 43).”

**Conclusions:** “In moderate-to-severe trauma- and postoperative-associated ARDS, EIT-guided PEEP titration was associated with improved oxygenation compared with a lower-PEEP/FiO₂ strategy while preserving lung-protective ventilatory parameters. These findings suggest the physiological feasibility of an EIT-guided individualized PEEP optimization strategy, although larger multicenter studies are needed to determine effects on patient-centered outcomes. ”

**Minor Concerns**

**The abstract and discussion continue to emphasize clinical outcomes such as ICU length of stay and SOFA trajectory, despite the study not being powered for these endpoints. Interpretation should be further tempered.**

We thank the reviewer for this important comment. We agree that secondary clinical outcomes should be interpreted cautiously because the trial was designed and powered for physiologic rather than clinical endpoints. These outcomes were exploratory and should not be interpreted as definitive evidence of clinical benefit.

In response, we further revised both the Abstract and Discussion to temper the presentation of ICU length of stay, SOFA score changes, and other clinical outcomes. These findings are now explicitly described as secondary exploratory outcomes, while emphasizing that larger adequately powered studies are required to determine whether physiologic improvements translate into patient-centered benefit.

We appreciate the reviewer’s comment, which has improved the balance and interpretation of the manuscript.

**Revision made:**

**Abstract (Results):** “Secondary clinical outcomes were broadly similar between groups. Exploratory analyses suggested greater SOFA score reduction and shorter ICU length of stay in the EIT group. Duration of mechanical ventilation, ventilator-free days, and 28-day mortality were similar between groups.”

**Discussion:**

“Secondary clinical outcomes were broadly similar between groups. Exploratory analyses suggested greater SOFA score reduction and shorter ICU length of stay in the EIT group. The trial was designed for physiologic rather than clinical endpoints, and larger studies are needed to determine whether these physiologic benefits translate into improved patient-centered outcomes.”

“Beyond respiratory physiology, exploratory secondary analyses showed greater SOFA score reduction and shorter ICU length of stay in the EIT group; however, these findings should be interpreted cautiously because the study was not powered for clinical endpoints.”

**Data availability statements appear to have been inconsistent across versions** **and should be fully aligned with journal policy.**

We thank the reviewer for this important comment. We agree that the Data Availability Statement should be fully consistent across all manuscript versions and clearly aligned with journal policy.

In response, we have revised and standardized the Data Availability Statement in the current version of the manuscript. The statement now consistently explains the basis for restricted public posting of participant-level data in this small single-center cohort, while confirming that deidentified data underlying the reported findings are available upon reasonable request through the Institutional Review Board of Hanoi Medical University, subject to applicable ethical and institutional requirements.

We appreciate the reviewer’s comment, which has improved the transparency and consistency of the manuscript.

**Revision made:**

**Data Availability Statement:** “Public posting of the participant-level dataset is limited because this study involved a small single-center cohort with residual risk of re-identification despite de-identification. Deidentified data underlying the findings are available upon reasonable request from the Institutional Review Board of Hanoi Medical University (daihocyhn@hmu.edu.vn), subject to applicable ethical and institutional requirements. Data are securely archived at Hanoi Medical University to ensure long-term availability.”

**Inclusion of the exact PEEP/FiO₂ table in supplementary material would improve reproducibility.**

We thank the reviewer for this helpful suggestion. We agree that inclusion of the exact lower PEEP/FiO₂ table used in the control group would improve transparency and reproducibility of the study protocol.

In response, we have added the complete lower PEEP/FiO₂ table as Supporting Information in the revised manuscript so that readers can clearly identify the comparator strategy and reproduce the protocol if desired.

We appreciate the reviewer’s comment, which has strengthened the reproducibility of the manuscript.

**Revision made:**

**Supporting Information:** **S1 Table. Lower PEEP/FiO₂ table used in the control group.**

**Some sentences in the Discussion remain overly long and could be improved for clarity.**

We thank the reviewer for this helpful comment. We agree that several sentences in the Discussion were overly long and could be improved for clarity and readability.

In response, we carefully edited the Discussion throughout the revised manuscript. Several long or complex sentences were shortened or divided into clearer statements, redundant wording was removed, and paragraph flow was improved while preserving the original scientific meaning.

We appreciate the reviewer’s comment, which has improved the readability of the manuscript.

**Revision made:**

**Discussion:** Long sentences were revised throughout the Discussion section, including the opening interpretation paragraph, the population characteristics paragraph, and the limitations section, to improve clarity, conciseness, and readability.
