# Supplementary material for: Electrical impedance tomography–guided individualized ventilation strategy in patients with trauma-related and postoperative acute respiratory distress syndrome
Source: PLoS One. 2026 Aug 24;21(8):e0355609. doi: 10.1371/journal.pone.0355609 (PMC13502546; doi:10.1371/journal.pone.0355609)
Supplement: Supplementary file 15 [file pone.0355609.s017.docx]

Manuscript Number: [PONE-D-26-05009R2] - [EMID:e49ba8f50d8c88f0]

Response to Reviewers and Editors

Roberta Ribeiro De Santis Santiago, M.D., Ph.D., R.R.T.

Academic Editor

PLOS ONE

We thank you for the opportunity to revise and resubmit our manuscript entitled “*Electrical impedance tomography–guided individualized ventilation strategy in patients with trauma-related and postoperative acute respiratory distress syndrome*.”

We sincerely appreciate the time and effort invested by the editor and reviewers in evaluating our work. Their insightful comments and constructive suggestions have been invaluable in improving the clarity and rigor of the manuscript. We have carefully addressed all comments and revised the manuscript accordingly. A revised version with tracked changes is provided, and detailed point-by-point responses are presented below, with our replies indicated in blue.

**Response to Reviewer #1**

We sincerely thank Reviewer #1 for confirming that all previous comments have been addressed. We greatly appreciate the reviewer’s constructive feedback throughout the revision process, which has helped improve the clarity and presentation of the manuscript.

**Response to Reviewer #2**

We sincerely thank Reviewer #2 for the positive reassessment and for indicating that there are no further comments. We appreciate the reviewer’s time and valuable input during the review process.

**Response to Reviewer #3**

We sincerely thank the reviewer for the careful reassessment of our manuscript and for the thoughtful, constructive comments. We greatly appreciate the recognition that the manuscript has improved substantially in methodological transparency, statistical rigor, and interpretation of findings, and that the remaining concerns primarily relate to interpretation rather than methodology. In response, we have revised the manuscript to consistently present the intervention as an EIT-guided individualized ventilation strategy, clarified its multicomponent nature, further tempered mechanistic interpretations, expanded the discussion of mechanical power, clarified statistical model diagnostics, and revised the Conclusions to emphasize physiological feasibility rather than superiority of EIT as a monitoring modality. Detailed point-by-point responses are provided below.

**Major comments**

1. **The intervention should consistently be presented as a bundled ventilation strategy rather than an isolated EIT intervention.**

**Although the Discussion has been revised to acknowledge this issue, the manuscript occasionally continues to imply that the observed physiological benefits resulted directly from EIT guidance. In reality, the intervention consisted of multiple simultaneous components, including recruitment maneuvers, decremental PEEP titration, repeated reassessment, individualized PEEP selection, and EIT monitoring. Consequently, the study compares two ventilation strategies rather than evaluating the independent effect of EIT itself. The wording throughout the manuscript should consistently reflect this distinction. Expressions such as “EIT-guided individualized ventilation strategy” or “EIT-guided PEEP optimization strategy” would more accurately describe the intervention than suggesting an isolated effect of EIT.**

We thank the reviewer for this important comment. We fully agree that the intervention should be interpreted as a multicomponent ventilation strategy rather than as an isolated evaluation of EIT monitoring. In response, we revised the manuscript throughout to use “EIT-guided individualized ventilation strategy” when referring to the overall intervention. When referring specifically to the PEEP component, we used “EIT-based PEEP titration,” “individualized PEEP selection,” or “protocol-selected PEEP,” as appropriate.

We also clarified that the trial compares ventilation strategies and does not establish the independent effect of EIT monitoring alone. The intervention components are now explicitly described as EIT monitoring, recruitment maneuvers, decremental PEEP titration, repeated reassessment, and individualized PEEP selection.

Revision made:

Title: “Electrical impedance tomography–guided individualized ventilation strategy in patients with trauma-related and postoperative acute respiratory distress syndrome”

Abstract (Methods): “In this single-center randomized controlled trial, 86 adults with moderate-to-severe trauma- or postoperative-associated ARDS were randomized to an EIT-guided individualized ventilation strategy (n = 43) or a lower-PEEP/FiO2 strategy (n = 43).”

Abstract (Methods): “In the EIT group, the multicomponent strategy included EIT monitoring, recruitment maneuvers, decremental PEEP titration, repeated reassessment, and individualized PEEP selection based on the intersection of regional collapse and overdistension curves.”

Methods (EIT group): “The EIT-guided individualized ventilation strategy comprised EIT monitoring, recruitment maneuvers, decremental PEEP titration, repeated reassessment, and individualized PEEP selection.”

Results: “Protocol adherence was high in the intervention arm: all analyzed patients underwent initial PEEP titration within the EIT-guided individualized ventilation strategy on Day 0, and repeat titration was performed on Days 1–3 in all patients with available follow-up.”

Discussion: “The intervention incorporated EIT monitoring, recruitment maneuvers, decremental PEEP titration, repeated reassessment, and individualized PEEP selection.”

Limitations: “Therefore, the observed oxygenation benefit should be interpreted as the effect of the multicomponent EIT-guided individualized ventilation strategy rather than as evidence of a specific EIT-derived mechanism.”

2. **Mechanistic interpretation remains limited.**

**The principal conceptual limitation of the study persists. The proposed mechanism underlying the intervention is optimization of the balance between regional collapse and overdistension. However, quantitative EIT-derived variables were not systematically recorded, and EIT was not performed in the control group. Therefore, although the study convincingly demonstrates improved oxygenation, it cannot establish whether this resulted from improved regional recruitment, reduced overdistension, improved ventilation-perfusion matching, higher PEEP exposure, recruitment maneuvers, or the combined effects of the bundled intervention. The current Discussion acknowledges this limitation but still occasionally implies mechanistic conclusions that are not directly supported by the available data. I would recommend making the mechanistic interpretation even more conservative.**

We thank the reviewer for this important and insightful comment. We agree that mechanistic interpretation should be more conservative. We further revised the Discussion, Limitations, and Conclusions to state explicitly that the mechanism underlying the oxygenation improvement cannot be determined from the present data because quantitative EIT-derived variables were not systematically collected and EIT was not performed in the control group.

The revised manuscript no longer attributes the oxygenation benefit to a specific EIT-derived mechanism. Instead, we explicitly acknowledge that the present design cannot distinguish among potential contributors, including regional recruitment, reduced overdistension, ventilation-perfusion matching, greater PEEP exposure, recruitment maneuvers, or the combined effects of the multicomponent intervention strategy. We also removed or tempered wording that could imply direct confirmation of regional ventilation mechanisms.

Revision made:

Discussion: “Because this trial evaluated a bundled EIT-guided ventilation strategy, without systematic collection of quantitative EIT-derived indices or EIT assessment in the control group, it cannot determine the mechanisms underlying the observed improvement in oxygenation or the relative contribution of individual intervention components.”

Discussion: “These features may partly explain the oxygenation improvement observed with the EIT-guided individualized ventilation strategy and differences from prior studies in more heterogeneous or predominantly medical ARDS cohorts, although the underlying mechanism could not be determined.”

Discussion: “Although quantitative indices of collapse and overdistension were not analyzed, PEEP titration within the intervention strategy was guided by real-time EIT assessment of regional ventilation.”

Limitations: “Finally, mechanistic interpretation was limited because EIT was not performed in the control group and quantitative EIT-derived indices were not systematically collected. Therefore, the observed oxygenation benefit should be interpreted as the effect of the multicomponent EIT-guided individualized ventilation strategy rather than as evidence of a specific EIT-derived mechanism.”

Conclusions: “These findings suggest the physiological feasibility of this strategy, but do not establish the independent effect of EIT monitoring or the mechanistic pathway underlying the observed oxygenation benefit.”

3. **Discussion of mechanical power could be strengthened.**

**The authors appropriately report that mechanical power normalized to predicted body weight was modestly higher in the intervention group despite similar driving pressures. However, the physiological interpretation remains relatively superficial. Mechanical power is influenced by several interacting determinants, including tidal volume, respiratory rate, inspiratory flow, PEEP, and driving pressure. Because driving pressure remained unchanged while PEEP was consistently higher in the intervention group, it is likely that the observed increase in MP/PBW primarily reflects greater PEEP exposure rather than worsening respiratory mechanics. Expanding this discussion would improve the physiological interpretation and place the findings in the context of current concepts regarding mechanical power and ventilator-induced lung injury.**

We thank the reviewer for this helpful comment. We agree that the physiological interpretation of MP/PBW required expansion. We revised the Discussion to clarify that mechanical power integrates several ventilatory determinants, including tidal volume, respiratory rate, inspiratory flow, driving pressure, and PEEP. We now interpret the modestly higher MP/PBW in the EIT group in relation to greater PEEP exposure within the EIT-guided individualized ventilation strategy, while noting that plateau pressure and driving pressure were not increased.

The revised Discussion also places this finding in the context of the MP framework proposed by Gattinoni et al., the analysis by Costa et al. showing that driving pressure and respiratory rate capture much of the prognostic information contained in mechanical power, and EIT-based evidence showing that recruitable alveolar collapse and regional hyperdistension can be estimated at the bedside during decremental PEEP titration. We further clarified that, because quantitative EIT-derived indices were not systematically collected and EIT was not performed in the control group, the findings remain physiologic and hypothesis-generating.

Revision made:

Discussion: “MP/PBW decreased over time in both groups, with no group-by-time interaction, indicating similar temporal trends. Overall MP/PBW was modestly higher in the EIT group.”

Discussion: “In the MP framework proposed by Gattinoni et al., MP integrates the ventilator-related load generated by VT, RR, inspiratory flow, Pdriv, and PEEP (5).”

Discussion: “In our study, higher MP/PBW paralleled greater PEEP exposure within the EIT-guided individualized ventilation strategy, while Pplat and Pdriv were not increased. This pattern supports a PEEP-related contribution to calculated MP/PBW rather than worsening respiratory mechanics or greater intrinsic lung injury.”

Discussion: “In an analysis of patients with ARDS, Costa et al. showed that MP was associated with outcome, but a simpler model using ΔP and RR captured comparable prognostic information, with ΔP having a greater effect than RR (37).”

Discussion: “In an EIT-based study during decremental PEEP titration, Costa et al. showed that recruitable alveolar collapse and regional hyperdistension can be estimated at the bedside and have regional distributions (7). This supports the concept that PEEP effects depend on lung morphology and recruitability (38).”

Discussion: “Because quantitative EIT-derived indices were not systematically collected and EIT was not performed in the control group, our findings should be interpreted as physiologic and hypothesis-generating, not as evidence of increased injurious regional lung stress or a specific EIT-derived mechanism.”

4. **The Conclusions should remain slightly more conservative.**

**The Conclusions have improved substantially compared with previous versions. Nevertheless, I would recommend emphasizing that the study demonstrates the physiological feasibility of an EIT-guided ventilation strategy rather than establishing superiority of EIT as a monitoring modality. Future studies incorporating quantitative EIT-derived measurements and parallel monitoring in both groups will be required to define the mechanistic pathways responsible for the observed physiological benefits.**

We thank the reviewer for this important recommendation. We agree and have revised both the Abstract Conclusions and the main Conclusions to emphasize physiological feasibility rather than superiority of EIT as a monitoring modality. The revised conclusions explicitly state that the study does not establish the independent effect of EIT monitoring or the mechanistic pathway underlying the observed oxygenation benefit.

We also added that future multicenter studies should include quantitative EIT-derived measurements and parallel regional ventilation monitoring in both groups to clarify mechanisms and determine effects on patient-centered outcomes.

Revision made:

Abstract (Conclusions): “These findings suggest the physiological feasibility of this strategy, but do not establish the independent effect of EIT monitoring or the underlying mechanistic pathway. Larger multicenter studies are needed to determine mechanisms and effects on patient-centered outcomes.”

Conclusions: “These findings suggest the physiological feasibility of this strategy, but do not establish the independent effect of EIT monitoring or the mechanistic pathway underlying the observed oxygenation benefit. Larger multicenter studies incorporating quantitative EIT-derived measurements and parallel regional ventilation monitoring in both groups are needed to determine effects on patient-centered outcomes.”

**Minor comments**

1. **Consider using consistent terminology throughout the manuscript. “EIT-guided individualized ventilation strategy” is scientifically more accurate than alternating between “EIT-guided ventilation” and “EIT-guided PEEP.”**

We thank the reviewer for this helpful comment. We agree that “EIT-guided individualized ventilation strategy” more accurately describes the intervention than alternating between “EIT-guided ventilation” and “EIT-guided PEEP.” We have therefore revised the manuscript to use “EIT-guided individualized ventilation strategy” when referring to the overall intervention. When referring specifically to the PEEP component, we use “EIT-based PEEP titration,” “individualized PEEP selection,” or “protocol-selected PEEP,” as appropriate. Group labels such as “EIT group” and “control group” were retained where appropriate for tables, figure legends, and concise reporting of group comparisons.

Revision made:

Terminology was revised throughout the title, Abstract, Introduction, Methods, Results, Discussion, Clinical implications, and Conclusions to frame the intervention as an EIT-guided individualized ventilation strategy rather than as an isolated EIT intervention or EIT-guided PEEP alone.

2. **The Discussion could benefit from a more detailed integration of recent literature regarding regional lung stress, recruitability, and mechanical power, particularly work from Costa, Gattinoni, and colleagues.**

We thank the reviewer for this helpful suggestion. We revised the Discussion to better integrate recent literature on regional lung heterogeneity, recruitability, mechanical power, and ventilator-induced lung injury. The revised text now discusses the MP framework proposed by Gattinoni et al., including the contribution of VT, RR, inspiratory flow, Pdriv, and PEEP to ventilator-related load (5). We also integrated the analysis by Costa et al. showing that MP is associated with outcome in ARDS, while ΔP and RR capture comparable prognostic information (37). In addition, we incorporated EIT-based evidence showing that recruitable alveolar collapse and regional hyperdistension can be estimated at the bedside during decremental PEEP titration (7).

We clarified that the modestly higher MP/PBW observed in the EIT group most likely reflected greater PEEP exposure within the EIT-guided individualized ventilation strategy rather than worsening respiratory mechanics. At the same time, we tempered the mechanistic interpretation because quantitative EIT-derived indices were not systematically collected and EIT was not performed in the control group.

Revision made:

Discussion: We expanded the MP/PBW paragraph to cite and discuss mechanical power and its determinants (5), ventilatory variables and mechanical power in ARDS (37), bedside EIT assessment of recruitable collapse and regional hyperdistension (7), and the dependence of PEEP response on lung morphology and recruitability (38).

3. **Although not mandatory, a brief statement confirming assessment of assumptions underlying the linear mixed-effects models (residual distribution and model diagnostics) would further strengthen confidence in the statistical analyses**.

We thank the reviewer for this helpful methodological suggestion. We have added a statement in the Statistical analysis section confirming that model assumptions were assessed by inspection of residual distributions and diagnostic plots.

Revision made:

Methods (Statistical analysis): “Model assumptions were assessed by inspection of residual distributions and diagnostic plots.”
