# Supplementary material for: Electrical impedance tomography–guided individualized ventilation strategy in patients with trauma-related and postoperative acute respiratory distress syndrome
Source: PLoS One. 2026 Aug 24;21(8):e0355609. doi: 10.1371/journal.pone.0355609 (PMC13502546; doi:10.1371/journal.pone.0355609)
Supplement: Peer review history

# Peer review: Decision Letter 0

**DOI:** 10.1371/journal.pone.0355609.r001

**Authors:** Roberta Ribeiro De Santis Santiago

16 Mar 2026

Dear Dr. Luu Quang,

==============================

The study addresses the role of individualized PEEP titration guided by EIT in patients with ARDS from a subgroup of trauma and post-surgical subjects. The authors conducted an RCT enrolling a subgroup of patients with ARDS, with the opportunity to provide a better understanding of the role of individualized mechanical ventilation. Overall, based on the reviewers' comments, it lacks novelty, and they raised major concerns and suggested revisions to clarify and provide more detail in the methodology and data analysis sections. I am adding the following comments to the reviewer's suggestions/concerns: The main results compare PaO2/FiO2, PEEP, Cstat, driving pressure, and mechanical power. Therefore, it is very important to address these measurements. In the data collection and outcomes section, it is unclear whether the driving pressure calculation accounted for the total PEEP in its formula (driving pressure = Plateau pressure - PEEP total) (page 7, line 179). It would be important to clarify this. Regarding the Cstat, I would suggest reassessing Cstat normalized by predicted body weight (per se based on height and sex) to strengthen the comparison between groups (Costa ELV et al. Am J Respir Crit Care Med. 2021 PMID: 33784486). In the discussion session (page 14, lines 398-400), the authors mentioned the potential benefit of EIT for assessing regional ventilation distribution, but the manuscript lacks exploration of regional ventilation and the mechanics of the study population. Do you have EIT data acquired in the control group for comparison? The manuscript lacks information about overdistension/collapse findings, which are the alleged advantages of the EIT approach. Have the authors identified any EIT information that could help phenotyping patients as advocated in the manuscript?

I attached two references as suggestions to your methods and discussion. I hope they can be helpful. Congratulations on your work. 

==============================

Please submit your revised manuscript by Apr 30 2026 11:59PM. If you will need more time than this to complete your revisions, please reply to this message or contact the journal office at plosone@plos.org. When you're ready to submit your revision, log on to https://www.editorialmanager.com/pone/ and select the 'Submissions Needing Revision' folder to locate your manuscript file.

- A letter that responds to each point raised by the academic editor and reviewer(s). You should upload this letter as a separate file labeled 'Response to Reviewers'.
- A marked-up copy of your manuscript that highlights changes made to the original version. You should upload this as a separate file labeled 'Revised Manuscript with Track Changes'.
- An unmarked version of your revised paper without tracked changes. You should upload this as a separate file labeled 'Manuscript'.

If you would like to make changes to your financial disclosure, please include your updated statement in your cover letter. Guidelines for resubmitting your figure files are available below the reviewer comments at the end of this letter.

If applicable, we recommend that you deposit your laboratory protocols in protocols.io to enhance the reproducibility of your results. Protocols.io assigns your protocol its own identifier (DOI) so that it can be cited independently in the future. For instructions see: https://journals.plos.org/plosone/s/submission-guidelines#loc-laboratory-protocols. Additionally, PLOS ONE offers an option for publishing peer-reviewed Lab Protocol articles, which describe protocols hosted on protocols.io. Read more information on sharing protocols at https://plos.org/protocols?utm_medium=editorial-email&utm_source=authorletters&utm_campaign=protocols.

We look forward to receiving your revised manuscript.

Kind regards,

Roberta Ribeiro De Santis Santiago, M.D., Ph.D., R.R.T.

Academic Editor

PLOS One

Journal Requirements:

When submitting your revision, we need you to address these additional requirements.

1. Please ensure that your manuscript meets PLOS ONE's style requirements, including those for file naming. The PLOS ONE style templates can be found at

https://journals.plos.org/plosone/s/file?id=wjVg/PLOSOne_formatting_sample_main_body.pdf and

https://journals.plos.org/plosone/s/file?id=ba62/PLOSOne_formatting_sample_title_authors_affiliations.pdf

2. PLOS requires an ORCID iD for the corresponding author in Editorial Manager on papers submitted after December 6th, 2016. Please ensure that you have an ORCID iD and that it is validated in Editorial Manager. To do this, go to ‘Update my Information’ (in the upper left-hand corner of the main menu), and click on the Fetch/Validate link next to the ORCID field. This will take you to the ORCID site and allow you to create a new iD or authenticate a pre-existing iD in Editorial Manager.

3. Your ethics statement should only appear in the Methods section of your manuscript. If your ethics statement is written in any section besides the Methods, please delete it from any other section.

4. In this instance it seems there may be acceptable restrictions in place that prevent the public sharing of your minimal data. However, in line with our goal of ensuring long-term data availability to all interested researchers, PLOS’ Data Policy states that authors cannot be the sole named individuals responsible for ensuring data access (http://journals.plos.org/plosone/s/data-availability#loc-acceptable-data-sharing-methods).

Data requests to a non-author institutional point of contact, such as a data access or ethics committee, helps guarantee long term stability and availability of data. Providing interested researchers with a durable point of contact ensures data will be accessible even if an author changes email addresses, institutions, or becomes unavailable to answer requests.

Before we proceed with your manuscript, please also provide non-author contact information (phone/email/hyperlink) for a data access committee, ethics committee, or other institutional body to which data requests may be sent. If no institutional body is available to respond to requests for your minimal data, please consider if there any institutional representatives who did not collaborate in the study, and are not listed as authors on the manuscript, who would be able to hold the data and respond to external requests for data access? If so, please provide their contact information (i.e., email address). Please also provide details on how you will ensure persistent or long-term data storage and availability.

5. Please ensure that you refer to Figure 2 in your text as, if accepted, production will need this reference to link the reader to the figure.

6. We notice that your supplementary tables are included in the manuscript file. Please remove them and upload them with the file type 'Supporting Information'. Please ensure that each Supporting Information file has a legend listed in the manuscript after the references list.

7. Please include captions for your Supporting Information files at the end of your manuscript, and update any in-text citations to match accordingly. Please see our Supporting Information guidelines for more information: http://journals.plos.org/plosone/s/supporting-information.

8. If the reviewer comments include a recommendation to cite specific previously published works, please review and evaluate these publications to determine whether they are relevant and should be cited. There is no requirement to cite these works unless the editor has indicated otherwise.

[Note: HTML markup is below. Please do not edit.]

Reviewers' comments:

Reviewer's Responses to Questions

**Comments to the Author**

1. Is the manuscript technically sound, and do the data support the conclusions?

Reviewer #1: Yes

Reviewer #2: Partly

**********

2. Has the statistical analysis been performed appropriately and rigorously?

Reviewer #1: Yes

Reviewer #2: I Don't Know

**********

3. Have the authors made all data underlying the findings in their manuscript fully available?

The PLOS Data policy

Reviewer #1: Yes

Reviewer #2: No

**********

4. Is the manuscript presented in an intelligible fashion and written in standard English?

Reviewer #1: Yes

Reviewer #2: Yes

**********

Reviewer #1: Thank you for the opportunity to review the manuscript entitled: Electrical Impedance Tomography–Guided Positive End-Expiratory Pressure Optimization in Patients With Trauma-Related and Postoperative Acute Respiratory Distress Syndrome. This is a well-designed single-center RCT addressing an important knowledge gap: individualized PEEP titration using EIT in trauma/postoperative ARDS. The study is methodologically sound, but it lacks a little bit novelty.

Major Points

1. Sample Size & Outcome Interpretation

Clearly state that the sample size was powered for a physiological (PaO2/FiO2) endpoint, not for detecting differences in clinical outcomes (e.g., mortality, VFDs). This is crucial for correctly interpreting the negative findings on these secondary endpoints throughout the study.

2. EIT Protocol & Adherence

Clarify the stability criterion during the decremental PEEP trial (30 seconds per step). Briefly justify or cite evidence that this duration is sufficient for stable alveolar recruitment/derecruitment in this population. Please provide actual protocol adherence data: How many patients in the EIT group underwent repeat titration on Days 1, 2, and 3? This is essential for interpreting the sustained physiological effects.

3. Control Group Strategy

Specify the exact "lower ARDS Network PEEP/FiO2 table" used (e.g., cite the source publication). Consider including the table in the supplement for reproducibility. The term "conventional low PEEP/FiO2 strategy" is vague. And why in the control group, RMs were not performed?

4. Subgroup Analysis Presentation

The analysis by pulmonary contusion status is interesting but must be explicitly labeled as exploratory and hypothesis-generating due to its post-hoc nature and limited sample size. For the key finding of higher ΔMP in contusion patients with EIT, provide the corresponding PEEP levels in this subgroup. This will help distinguish the effect of the EIT method from the effect of simply applying higher PEEP in this specific pathology.

5. Discussion & Interpretation

Specify the unique pathophysiology of trauma/postoperative ARDS (e.g., chest wall mechanics, lower inflammatory burden) and how it might influence responses to EIT-guided PEEP compared to sepsis-related ARDS.

Minor Points

1. Provide a clear definition for "Weaning success" in the Methods or Table S2 footnote.

2. Some sentences, particularly in the Discussion, are too long.

3. Double-check that all abbreviations are defined at first use.

Reviewer #2: This single center RCT compared electrical impedance tomography vs. usual to assess if oxygenation and other characteristics differed.

1. Data availability statement does not match information provided in how the data may be accessed.

2. What is the purpose of the "Etiologic Classification" in th emethods section? I'm not sure this is the best place for this.

3. For the primary outcomes - which day is the primary outcome, and is the primary outcome the absolute level or the change from baseline, or is it all of the days - e.g. mean over 3 days, or change over three days.

4.For the longitudinal models - please add details about e.g. Pillai's trace, and the adjustment for multiple comparisons made

5. How was missing data dealt with in the non-repeated measures analyses?

6. Did you assess sphericity in your repeated measures glm?

7. Usual implementations of repeated measures anova drop subjects who are missing even one measure - is this what you did? A more flexible approach would be to use a linear mixed model.

8. Was an intention to treat approach used?

9. Remove p values from Table S1.

10. How were deaths dealt with in the analyses? This is the most important point raised here. Please carefully consider how death was dealt with in the primary analyses, as well as the clinical comparisons.

11. Ensure that the measures presented in Figure 4 are described in the methods section.

**********

what does this mean?). If published, this will include your full peer review and any attached files.

If you choose “no”, your identity will remain anonymous but your review may still be made public.

**Do you want your identity to be public for this peer review?**  For information about this choice, including consent withdrawal, please see our Privacy Policy

Reviewer #1: No

Reviewer #2: No

**********

[NOTE: If reviewer comments were submitted as an attachment file, they will be attached to this email and accessible via the submission site. Please log into your account, locate the manuscript record, and check for the action link "View Attachments". If this link does not appear, there are no attachment files.]

To ensure your figures meet our technical requirements, please review our figure guidelines: https://journals.plos.org/plosone/s/figures

You may also use PLOS’s free figure tool, NAAS, to help you prepare publication quality figures: https://journals.plos.org/plosone/s/figures#loc-tools-for-figure-preparation.

NAAS will assess whether your figures meet our technical requirements by comparing each figure against our figure specifications.

Attachment Submitted filename: costa-et-al-2021-ventilatory-variables.pdf

Attachment Submitted filename: Lung morphology.pdf

# Author comment: Author response to Decision Letter 1

**DOI:** 10.1371/journal.pone.0355609.r002

30 Mar 2026

Manuscript Number: [PONE-D-26-05009] - [EMID:b4caae6824179d2a]

Response to Reviewers and Editors

Roberta Ribeiro De Santis Santiago, M.D., Ph.D., R.R.T.

Academic Editor

PLOS One

We thank you for the opportunity to revise and resubmit our manuscript entitled “Electrical Impedance Tomography–Guided Positive End-Expiratory Pressure Optimization in Patients With Trauma-Related and Postoperative Acute Respiratory Distress Syndrome.” We sincerely appreciate the time and effort invested by the editor and reviewers in evaluating our work. Their insightful comments and constructive suggestions have been invaluable in improving the clarity and rigor of the manuscript. We have carefully addressed all comments and revised the manuscript accordingly. Changes are highlighted in yellow in the revised version, and detailed, point-by-point responses are provided below, with our replies indicated in blue.

Response to Academic Editor

We thank the Academic Editor for the careful and thoughtful evaluation of our manuscript. We appreciate the constructive and insightful comments, which have helped improve the clarity, methodological transparency, and interpretation of our study. We have revised the manuscript accordingly and addressed all concerns in detail below.

- The study addresses the role of individualized PEEP titration guided by EIT in patients with ARDS from a subgroup of trauma and post-surgical subjects. The authors conducted an RCT enrolling a subgroup of patients with ARDS, with the opportunity to provide a better understanding of the role of individualized mechanical ventilation. Overall, based on the reviewers' comments, it lacks novelty, and they raised major concerns and suggested revisions to clarify and provide more detail in the methodology and data analysis sections.

We appreciate this important comment. We have revised the Introduction and Discussion to more clearly position the novelty of our study. Specifically, our trial focuses on a relatively underrepresented ARDS population-trauma- and postoperative-associated ARDS-characterized by distinct patterns of regional heterogeneity and recruitability compared with predominantly medical ARDS cohorts. We have emphasized that the study contributes to the field by:

• Evaluating EIT-guided PEEP titration in a clinically distinct ARDS population.

• Exploring phenotype-specific physiologic responses (pulmonary contusion vs non-contusion).

• Integrating mechanical power and compliance responses into the interpretation of individualized ventilation strategies.

We have clarified these points to better contextualize the contribution of the study.

- I am adding the following comments to the reviewer's suggestions/concerns: The main results compare PaO2/FiO2, PEEP, Cstat, driving pressure, and mechanical power. Therefore, it is very important to address these measurements. In the data collection and outcomes section, it is unclear whether the driving pressure calculation accounted for the total PEEP in its formula (driving pressure = Plateau pressure - PEEP total) (page 7, line 179). It would be important to clarify this.

We thank the reviewer for this important comment. We have carefully addressed these points in detail in our responses to the reviewer below and have revised the manuscript accordingly.

- Regarding the Cstat, I would suggest reassessing Cstat normalized by predicted body weight (per se based on height and sex) to strengthen the comparison between groups (Costa ELV et al. Am J Respir Crit Care Med. 2021 PMID: 33784486).

We thank the Editor for this insightful suggestion. We agree that normalization of respiratory mechanics to predicted body weight (PBW) may improve comparability across patients of different body size.

We note that the referenced study by Costa et al. primarily emphasizes mechanical power normalized to PBW (MP/PBW) as a physiologically integrative metric that incorporates multiple components of ventilatory load. In line with this approach, our study already included MP normalized to PBW (MP/PBW) as a prespecified secondary physiologic outcome.

As shown in our Results, MP/PBW decreased over time in both groups, with modestly higher values in the EIT group but without a significant group-by-time interaction, indicating similar temporal trends between groups. These findings are presented and interpreted in both the Results and Discussion sections.

By contrast, static compliance (Cstat) was reported in absolute terms. In trauma- and postoperative-associated ARDS, Cstat reflects the combined effects of lung and chest wall mechanics, which may vary substantially due to injury-related and postoperative factors. We therefore considered absolute Cstat more appropriate for physiologic interpretation in this population.

We have cited the suggested reference (Costa ELV et al.) and clarified this rationale in the revised manuscript.

- In the discussion session (page 14, lines 398-400), the authors mentioned the potential benefit of EIT for assessing regional ventilation distribution, but the manuscript lacks exploration of regional ventilation and the mechanics of the study population. Do you have EIT data acquired in the control group for comparison?

We thank the Editor for this important comment. In the present study, EIT was used as a bedside clinical tool to guide individualized PEEP titration in the intervention group rather than as a modality for comprehensive assessment of regional ventilation mechanics.

EIT monitoring was not performed in the control group because the study was designed to compare an EIT-guided strategy with a standard-of-care approach, without introducing additional monitoring or co-interventions in the control arm. This approach was intended to preserve the pragmatic nature of the comparator and to ensure that observed differences were primarily attributable to the EIT-guided PEEP titration strategy.

As a result, direct comparison of regional ventilation patterns between groups was not feasible. We have clarified this point in the revised manuscript and explicitly acknowledged it as a limitation.

- The manuscript lacks information about overdistension/collapse findings, which are the alleged advantages of the EIT approach.

We thank the Editor for this important comment. We agree that assessment of regional overdistension and collapse represents a key advantage of EIT. In the present study, EIT-derived information on regional collapse and overdistension was used in real time to guide individualized PEEP selection in the intervention group. However, these quantitative data were not systematically recorded for subsequent analysis, as the primary aim of the study was to evaluate physiologic outcomes associated with an EIT-guided PEEP strategy rather than to perform a detailed mechanistic analysis of regional ventilation patterns.

As a result, we were unable to provide formal analyses of overdistension and collapse across time or between groups. This limitation has now been explicitly acknowledged in the revised manuscript.

- Have the authors identified any EIT information that could help phenotyping patients as advocated in the manuscript?

We thank the Editor for this insightful comment. We agree that EIT has the potential to support phenotyping based on regional ventilation patterns.

In the present study, however, EIT was not used for systematic phenotypic classification, as its primary role was to guide individualized PEEP titration in the intervention group. Quantitative EIT-derived indices were not collected in a manner that would allow formal phenotyping analysis.

Instead, we performed prespecified subgroup analyses based on pulmonary contusion, representing a clinically relevant phenotype of traumatic lung injury. This approach was chosen to explore whether underlying patterns of lung injury, particularly structural disruption associated with pulmonary contusion, may influence physiologic responses to PEEP.

We have clarified in the revised manuscript that these subgroup analyses are exploratory and hypothesis-generating, and that further studies incorporating detailed EIT-derived regional metrics may help refine phenotype-based ventilation strategies.

Response to Reviewer #1

We thank the reviewer for the thorough and constructive evaluation of our manuscript. We appreciate the recognition of the methodological rigor and clinical relevance of our study, as well as the insightful comments provided. These suggestions have been invaluable in improving the clarity, consistency, and interpretation of the manuscript. We have carefully revised the manuscript to address all comments, as detailed below.

Major Points

1. Sample Size & Outcome Interpretation: Clearly state that the sample size was powered for a physiological (PaO2/FiO2) endpoint, not for detecting differences in clinical outcomes (e.g., mortality, VFDs). This is crucial for correctly interpreting the negative findings on these secondary endpoints throughout the study.

We thank the reviewer for this important comment. We have revised the manuscript to clarify that the sample size was based on PaO₂/FiO₂ as the primary physiologic endpoint and that the study was not designed or sufficiently powered to detect differences in clinical outcomes. We also revised the Discussion to present clinical outcomes as secondary exploratory findings rather than definitive evidence of clinical benefit . Revision made:

Methods (Statistical Analysis): “Sample size estimation was based on a two-sided comparison of independent means (α = 0.05, power = 90%) using Day 1 PaO₂/FiO₂, with allowance for 20% attrition.”

Discussion: ‘’Although 28-day mortality did not differ between groups, duration of ventilation and ICU length of stay were shorter in the EIT group. These outcomes were secondary and exploratory. The study was designed and powered for physiologic rather than clinical endpoints; therefore, these findings should not be interpreted as definitive evidence of clinical benefit. In addition, duration of ventilation and ICU length of stay may be influenced by non-respiratory factors, including sedation practices, weaning strategies, surgical recovery, and ICU discharge decisions. Further studies specifically powered for clinical outcomes are required to determine whether the observed physiologic improvements translate into reproducible clinical benefit.’’

2. EIT Protocol & Adherence: Clarify the stability criterion during the decremental PEEP trial (30 seconds per step). Briefly justify or cite evidence that this duration is sufficient for stable alveolar recruitment/derecruitment in this population. Please provide actual protocol adherence data: How many patients in the EIT group underwent repeat titration on Days 1, 2, and 3? This is essential for interpreting the sustained physiological effects.

We thank the reviewer for this important comment. We have clarified the stability criterion used during the decremental PEEP trial and provided justification with appropriate references. Each PEEP level was maintained for 30 seconds to allow stabilization of regional ventilation signals before proceeding to the next step. This approach is consistent with previously described EIT-guided decremental PEEP protocols, in which PEEP is reduced in stepwise increments with short stabilization periods (e.g., 30 seconds per step) during EIT-based titration (van der Zee P, Somhorst P, Endeman H, Gommers D. Electrical Impedance Tomography for Positive End-Expiratory Pressure Titration in COVID-19-related Acute Respiratory Distress Syndrome. Am J Respir Crit Care Med. 2020;202(2):280-284. doi:10.1164/rccm.202003-0816LE).

Regarding protocol adherence, all patients in the intervention group underwent initial EIT-guided PEEP titration on Day 0. In the EIT group, 43 patients were randomized, and one patient was transferred early and was not further followed, leaving 42 patients with available follow-up. Repeat EIT-guided PEEP titration was planned on Days 1–3 according to the study protocol, with predefined safety criteria. In the present study, no patients met these criteria during the protocolized period. Accordingly, repeat titration was performed in all 42 patients with available follow-up on Day 1, Day 2, and Day 3. This reflects full protocol adherence during the study period. We have clarified these points in the revised Methods and Results sections.

Methods:

“A decremental PEEP titration followed, starting at 24 cmH₂O and decreasing in 2 cmH₂O steps to a minimum of 6 cmH₂O or until SpO₂ ≤80% with each level maintained for 30 seconds. This duration was chosen to allow stabilization of regional ventilation signals, consistent with prior EIT-guided decremental PEEP protocols (26).”

“EIT-guided PEEP reassessment was performed according to protocol on Days 1–3 in all patients with available follow-up in the EIT group.”

Results:

“Protocol adherence was high in the intervention arm: all analyzed patients underwent initial EIT-guided PEEP titration on Day 0, and repeat titration was performed on Days 1–3 in all patients with available follow-up.”

3. Control Group Strategy: Specify the exact "lower ARDS Network PEEP/FiO2 table" used (e.g., cite the source publication). Consider including the table in the supplement for reproducibility. The term "conventional low PEEP/FiO2 strategy" is vague. And why in the control group, RMs were not performed?

We thank the reviewer for this important comment. We have clarified the control group strategy to improve precision and reproducibility. As described in the revised Methods, PEEP in the control group was applied according to the lower-PEEP/FiO2 strategy of the ARDS Network ALVEOLI trial, using predefined PEEP–FiO₂ combinations, and the corresponding reference has been added (Brower RG, Lanken PN, MacIntyre N, Matthay MA, Morris A, Ancukiewicz M, et al. Higher versus lower positive end-expiratory pressures in patients with the acute respiratory distress syndrome. N Engl J Med. 2004;351(4):327–36. doi: 10.1056/NEJMoa032193. PubMed PMID: 15269312). To avoid ambiguity, we have replaced the term “conventional low PEEP/FiO₂ strategy” with this more specific description.

Routine recruitment maneuvers were not performed to reflect a standard comparator strategy and to avoid additional co-interventions beyond the protocolized PEEP approach. This approach is consistent with current ESICM guidelines, which suggest against the routine use of brief high-pressure recruitment maneuvers (≥35 cmH₂O for <1 minute) in patients with ARDS, as no mortality benefit has been demonstrated (Grasselli G, Calfee CS, Camporota L, et al. ESICM guidelines on acute respiratory distress syndrome: definition, phenotyping and respiratory support strategies. Intensive Care Med. 2023;49(7):727-759. doi:10.1007/s00134-023-07050-7).

Revision made (Methods):

“PEEP was applied according to the lower-PEEP/FiO2 strategy of the ARDS Network ALVEOLI trial, using predefined PEEP–FiO₂ combinations (27).”

“This approach is consistent with contemporary ESICM guidelines, which suggest against the routine use of brief high-pressure recruitment maneuvers in ARDS due to the absence of demonstrated mortality benefit (28).”

4. Subgroup Analysis Presentation: The analysis by pulmonary contusion status is interesting but must be explicitly labeled as exploratory and hypothesis-generating due to its post-hoc nature and limited sample size. For the key finding of higher ΔMP in contusion patients with EIT, provide the corresponding PEEP levels in this subgroup. This will help distinguish the effect of the EIT method from the effect of simply applying higher PEEP in this specific pathology.

We thank the reviewer for this insightful comment. We agree that the subgroup analysis should be interpreted with appropriate caution. We have clarified throughout the manuscript that the analyses stratified by pulmonary contusion status were prespecified but exploratory and not powered for definitive subgroup comparisons. Accordingly, these findings are now consistently described as hypothesis-generating. Regarding the observation of higher ΔMP in the EIT group, we agree that PEEP is a key determinant of mechanical power. As reported in the Results, PEEP levels were higher in the EIT group at later time points across both subgroups, without a signif

Attachment Submitted filename: Response_to_Reviewers.docx

# Peer review: Decision Letter 1

**DOI:** 10.1371/journal.pone.0355609.r003

**Authors:** Roberta Ribeiro De Santis Santiago

26 Apr 2026

Dear Dr. Luu Quang,

Thank you for submitting your manuscript to PLOS ONE for addressing the reviewers' comments and concerns. After careful consideration, we feel that it has merit; therefore, we invite you to submit a revised version of the manuscript that addresses the points raised during the review process.

Please submit your revised manuscript by Jun 10 2026 11:59PM. If you will need more time than this to complete your revisions, please reply to this message or contact the journal office at plosone@plos.org. When you're ready to submit your revision, log on to https://www.editorialmanager.com/pone/ and select the 'Submissions Needing Revision' folder to locate your manuscript file.

- A letter that responds to each point raised by the academic editor and reviewer(s). You should upload this letter as a separate file labeled 'Response to Reviewers'.
- A marked-up copy of your manuscript that highlights changes made to the original version. You should upload this as a separate file labeled 'Revised Manuscript with Track Changes'.
- An unmarked version of your revised paper without tracked changes. You should upload this as a separate file labeled 'Manuscript'.

If you would like to make changes to your financial disclosure, please include your updated statement in your cover letter. Guidelines for resubmitting your figure files are available below the reviewer comments at the end of this letter.

If applicable, we recommend that you deposit your laboratory protocols in protocols.io to enhance the reproducibility of your results. Protocols.io assigns your protocol its own identifier (DOI) so that it can be cited independently in the future. For instructions see: https://journals.plos.org/plosone/s/submission-guidelines#loc-laboratory-protocols. Additionally, PLOS ONE offers an option for publishing peer-reviewed Lab Protocol articles, which describe protocols hosted on protocols.io. Read more information on sharing protocols at https://plos.org/protocols?utm_medium=editorial-email&utm_source=authorletters&utm_campaign=protocols.

As the corresponding author, your ORCID iD is verified in the submission system and will appear in the published article. PLOS supports the use of ORCID, and we encourage all coauthors to register for an ORCID iD and use it as well. Please encourage your coauthors to verify their ORCID iD within the submission system before final acceptance, as unverified ORCID iDs will not appear in the published article. *Only*  the individual author can complete the verification step; PLOS staff *cannot*  verify ORCID iDs on behalf of authors.

We look forward to receiving your revised manuscript.

Kind regards,

Roberta Ribeiro De Santis Santiago, M.D., Ph.D., R.R.T.

Academic Editor

PLOS One

Journal Requirements:

If the reviewer comments include a recommendation to cite specific previously published works, please review and evaluate these publications to determine whether they are relevant and should be cited. There is no requirement to cite these works unless the editor has indicated otherwise.

Reviewers' comments:

Reviewer's Responses to Questions

**Comments to the Author**

Reviewer #1: All comments have been addressed

Reviewer #2: All comments have been addressed

Reviewer #3: All comments have been addressed

**********

2. Is the manuscript technically sound, and do the data support the conclusions?

Reviewer #1: Yes

Reviewer #2: Yes

Reviewer #3: Partly

**********

3. Has the statistical analysis been performed appropriately and rigorously?

Reviewer #1: Yes

Reviewer #2: Yes

Reviewer #3: No

**********

4. Have the authors made all data underlying the findings in their manuscript fully available?

The PLOS Data policy

Reviewer #1: Yes

Reviewer #2: No

Reviewer #3: Yes

**********

5. Is the manuscript presented in an intelligible fashion and written in standard English?

Reviewer #1: Yes

Reviewer #2: Yes

Reviewer #3: Yes

**********

Reviewer #1: The author provided detailed & appropriate responses to all of my comments.

The only minor concern is the Figures, which have room for improvement. For example, resolution of the Figure 1, which would be better if it is a original screenshot.

Reviewer #2: (No Response)

Reviewer #3: This is a single-center randomized controlled trial evaluating an EIT-guided PEEP titration strategy in trauma- and postoperative-associated ARDS, a clinically relevant and underrepresented population. The authors should be commended for addressing an important and challenging aspect of mechanical ventilation—individualized PEEP selection in heterogeneous lung injury.

The manuscript has improved after revision, particularly in clarifying that the study was powered for physiologic rather than clinical endpoints and in tempering some of the initial overinterpretation. However, several important methodological and interpretive concerns remain, which limit the robustness of the conclusions.

**********

what does this mean?). If published, this will include your full peer review and any attached files.

If you choose “no”, your identity will remain anonymous but your review may still be made public.

**Do you want your identity to be public for this peer review?**  For information about this choice, including consent withdrawal, please see our Privacy Policy

Reviewer #1: **Yes:**  Changsheng Zhang

Reviewer #2: No

Reviewer #3: **Yes:**  Bruno Tonelotto

**********

[NOTE: If reviewer comments were submitted as an attachment file, they will be attached to this email and accessible via the submission site. Please log into your account, locate the manuscript record, and check for the action link "View Attachments". If this link does not appear, there are no attachment files.]

To ensure your figures meet our technical requirements, please review our figure guidelines: https://journals.plos.org/plosone/s/figures

You may also use PLOS’s free figure tool, NAAS, to help you prepare publication quality figures: https://journals.plos.org/plosone/s/figures#loc-tools-for-figure-preparation.

NAAS will assess whether your figures meet our technical requirements by comparing each figure against our figure specifications.

Attachment Submitted filename: Reviewer Coments - PONE-D-26-05009R1.pdf

# Author comment: Author response to Decision Letter 2

**DOI:** 10.1371/journal.pone.0355609.r004

9 May 2026

Manuscript Number: [[PONE-D-26-05009R1] - [EMID:f34553fd3da164d7]

Response to Reviewers and Editors

Roberta Ribeiro De Santis Santiago, M.D., Ph.D., R.R.T.

Academic Editor

PLOS One

We thank you for the opportunity to revise and resubmit our manuscript entitled “Electrical Impedance Tomography–Guided Positive End-Expiratory Pressure Optimization in Patients With Trauma-Related and Postoperative Acute Respiratory Distress Syndrome.” We sincerely appreciate the time and effort invested by the editor and reviewers in evaluating our work. Their insightful comments and constructive suggestions have been invaluable in improving the clarity and rigor of the manuscript. We have carefully addressed all comments and revised the manuscript accordingly. A revised version with tracked changes is provided, and detailed point-by-point responses are presented below, with our replies indicated in blue.

Response to Reviewer #1

We thank the reviewer for the thorough and constructive evaluation of our manuscript. We appreciate the recognition of the methodological rigor and clinical relevance of our study, as well as the insightful comments provided. These suggestions have been invaluable in improving the clarity, consistency, and interpretation of the manuscript. We have carefully revised the manuscript to address all comments, as detailed below.

The author provided detailed & appropriate responses to all of my comments.

The only minor concern is the Figures, which have room for improvement. For example, resolution of the Figure 1, which would be better if it is a original screenshot.

We sincerely thank the reviewer for the positive assessment of our revisions and for the helpful final suggestion regarding the figures. In response, we have improved the overall figure quality and replaced Figure 1 with a higher-resolution original system screenshot/export to enhance image clarity and readability. The figure formatting and legend have also been refined to improve presentation quality in the revised manuscript. We appreciate this valuable recommendation.

Response to Reviewer #3

We sincerely thank the reviewer for the careful reassessment of our manuscript and for the thoughtful, constructive comments. We greatly appreciate the recognition of the clinical relevance of this trial and of the improvements made in the revised version, particularly regarding clarification that the study was powered for physiologic rather than clinical endpoints and the more cautious interpretation of secondary outcomes. We also appreciate the reviewer’s additional methodological and interpretive concerns, which have been highly valuable in further strengthening the manuscript. In response, we have undertaken substantial additional revisions, including clarification of the modified intention-to-treat analysis population, replacement of repeated-measures analyses with linear mixed-effects modeling for longitudinal outcomes, expanded discussion of missing data and mechanistic limitations, clearer acknowledgment that the intervention represented a bundled EIT-guided PEEP optimization strategy, and further tempering of subgroup and clinical outcome interpretations. Detailed point-by-point responses to each comment are provided below.

Major Concerns

1. Analysis population and internal inconsistency (critical issue)

There is a significant inconsistency between the original manuscript and the response to reviewers regarding the analytical framework. The manuscript suggests that all randomized patients were included in the analysis, implying an intention-to-treat approach. However, the authors clarify in their response that analyses were performed only in patients with available follow-up data, without a full intention-to-treat framework.

This discrepancy is not trivial. It directly impacts the validity of the randomized comparison and must be explicitly clarified in the manuscript. The analytical population should be clearly defined (e.g., modified intention-to-treat, per-protocol), and the implications of excluding patients lost to follow-up must be discussed.

We thank the reviewer for this important comment. We agree that the analytical population required clearer and fully consistent reporting. We have revised the manuscript to explicitly define the primary analysis population as a modified intention-to-treat (mITT) population, consisting of all randomized patients who received the allocated intervention and had at least one post-randomization outcome assessment available. This clarification has now been incorporated consistently in the Abstract, Methods, Results, Fig 2, and Limitations section.

Four randomized patients were transferred early to other hospitals before analyzable follow-up data could be collected and therefore had no post-randomization longitudinal physiologic measurements available for inclusion in the prespecified repeated-measures analyses. These patients were excluded from the mITT population because the co-primary physiologic outcomes required post-randomization follow-up assessments.

We also agree that the implications of these exclusions should be discussed. Accordingly, we have added a limitation acknowledging that exclusion of these early-transferred patients means the analysis was not a full intention-to-treat analysis and may introduce limited attrition bias, although the number of excluded patients was small (4/86) and reasonably balanced between groups (1 in EIT, 3 in control).

Revision made:

Abstract (Methods): “Four patients were transferred early before analyzable follow-up data were collected; therefore, the modified intention-to-treat population included 42 patients in the EIT group and 40 in the control group.”

Methods (Statistical analysis): “The primary analysis population was a modified intention-to-treat population, defined as all randomized patients who received the allocated intervention and had at least one post-randomization outcome assessment available. Patients transferred before collection of analyzable follow-up data were excluded.”

Results: “Accordingly, the modified intention-to-treat population included 82 patients, comprising 42 patients in the EIT group and 40 patients in the control group.”

Limitations: “Because four randomized patients were transferred early and had no analyzable post-randomization follow-up data, the analysis was not a full intention-to-treat analysis. Although the proportion of excluded patients was small (4/86), this may introduce limited attrition bias and should be considered when interpreting the randomized comparison.”

2. Handling of longitudinal data and choice of statistical model

The authors used repeated-measures general linear models despite the presence

of missing data due to early transfer and potentially incomplete measurements. While

the proportion of missing data is relatively small, the choice of model is suboptimal.A linear mixed-effects model would be more appropriate, as it better handles unbalanced longitudinal data and reduces the risk of bias associated with complete-case or implicitly balanced approaches. The current justification for retaining repeated-measures GLM is not entirely convincing, particularly for a randomized longitudinal study.

We thank the reviewer for this important methodological comment and agree that a linear mixed-effects modeling approach is more appropriate for longitudinal outcomes in the presence of unbalanced repeated measurements. In response, we have revised the statistical analysis and replaced the previous repeated-measures general linear model analyses with linear mixed-effects models (LMMs) for all longitudinal outcomes.

The revised models included fixed effects for treatment group, time, and the group-by-time interaction, with subject-specific random intercepts and an autoregressive covariance structure to account for within-subject correlation across repeated measurements. This approach uses all available observations without requiring complete data at every time point and is more robust to missing follow-up measurements due to early transfer, death, or clinical deterioration.

We have updated the Methods, Results, figure legends, and Supporting Information tables accordingly. Importantly, the principal findings were materially unchanged after reanalysis, with sustained between-group differences in oxygenation favoring the EIT group and no major changes in the interpretation of respiratory mechanics or clinical outcomes. This consistency strengthens the robustness of the reported physiologic effects.

Revision made:

Methods (Statistical analysis): “Longitudinal outcomes during Days 0–3 were analyzed using linear mixed-effects models with fixed effects for treatment group, time, and the group-by-time interaction, with subject-specific random intercepts and an autoregressive covariance structure. This approach used all available repeated measurements while accounting for within-subject correlation.”

Results: All repeated longitudinal analyses for PaO₂/FiO₂, ΔPaO₂/FiO₂, PEEP, Cstat, Pplat, Pdriv, MP/PBW, and exploratory subgroup outcomes were reanalyzed using linear mixed-effects models, and corresponding statistics have been updated throughout the manuscript.

Figure legends (Figs 3–5): “Longitudinal changes were analyzed using linear mixed-effects models with fixed effects for group, time, and group-by-time interaction, with Sidak-adjusted post hoc comparisons where applicable.”

Supporting Information: Updated subgroup model outputs and pairwise comparisons are now presented in S2–S4 Tables based on linear mixed-effects models.

3. Handling of missing data and deaths

The manuscript states that only observed physiologic data during Days 0–3 were analyzed, with no imputation after death or loss to follow-up. This approach assumes that missingness is non-informative, which may not be valid.

In critically ill populations, missing data due to death or clinical deterioration is often informative and may bias results toward survivors. The authors should more explicitly discuss this limitation and its potential impact on the physiologic comparisons.

We thank the reviewer for this important comment. We agree that missing physiologic data due to death, clinical deterioration, or early transfer in critically ill populations may be informative rather than completely random, and that this limitation should be explicitly acknowledged.

In response, we have revised the manuscript to clarify that longitudinal outcomes during Days 0–3 were analyzed using linear mixed-effects models based on all available observed repeated measurements, with no imputation performed after death, transfer, or loss to follow-up. This approach allows use of incomplete repeated data while accounting for within-subject correlation, but does not eliminate the possibility of informative missingness.

We have therefore expanded the Limitations section to explicitly state that missing physiologic data after early transfer, death, or clinical deterioration may not have been random, and that longitudinal comparisons during Days 0–3 should be interpreted cautiously. We appreciate the reviewer’s comment, which has improved the transparency and interpretation of the study findings.

Revision made:

Methods (Statistical analysis):

“Longitudinal outcomes during Days 0–3 were analyzed using linear mixed-effects models with fixed effects for treatment group, time, and the group-by-time interaction, with subject-specific random intercepts and an autoregressive covariance structure. This approach used all available repeated measurements while accounting for within-subject correlation.”

“No imputation was performed after death, transfer, or loss to follow-up.”

Discussion (Limitations):

“Missing physiologic data after early transfer, death, or clinical deterioration may not have been random; therefore, longitudinal comparisons during Days 0–3 should be interpreted cautiously. No post-event imputation was performed.”

4. Intervention versus comparator asymmetry

The intervention arm includes recruitment maneuvers and repeated dynamic PEEP titration guided by EIT, whereas the control group follows a lower-PEEP/FiO₂ table without recruitment maneuvers.

This represents more than a monitoring strategy comparison; it constitutes a bundled intervention. Therefore, the observed differences cannot be attributed solely to EIT guidance, but rather to the combined effect of recruitment maneuvers, higher PEEP exposure, and repeated titration. This should be explicitly acknowledged and discussed.

We thank the reviewer for this important comment. We agree that the intervention arm represented more than a monitoring-only comparison. The EIT group received a protocolized strategy that incorporated recruitment maneuvers, decremental titration, repeated reassessment, and individualized PEEP adjustment, whereas the control group received a lower-PEEP/FiO₂ table strategy without routine recruitment maneuvers. We therefore agree that the observed differences cannot be attributed solely to EIT monitoring itself.

In response, we have revised the manuscript to consistently describe the intervention as an EIT-guided PEEP optimization strategy rather than EIT guidance alone. We have also strengthened the Discussion and Clinical Implications sections to explicitly acknowledge that the observed physiologic effects likely reflect the combined influence of recruitment maneuvers, higher PEEP exposure at later time points, repeated titration, and regional monitoring-informed adjustment. Accordingly, the trial should be interpreted as a comparison of two ventilation strategies rather than an isolated test of bedside monitoring technology.

We appreciate the reviewer’s comment, which has improved the conceptual framing and interpretation of the study.

Revision made:

Discussion: “In this randomized controlled trial of moderate-to-severe trauma- and postoperative-associated ARDS, an EIT-guided PEEP optimization strategy was associated with greater improvement in oxygenation than a lower-PEEP/FiO₂ strategy. Measurements were obtained after post-titration stabilization. Therefore, sustained differences were more likely related to individualized PEEP selection than to transient recruitment effects alone.”

Clinical implications and future directions: “Our findings support the feasibility of an EIT-guided bundled PEEP optimization strategy and suggest a physiologic oxygenation benefit in trauma- and postoperative-associated ARDS.”

Conclusions: “These findings suggest the physiological feasibility of an EIT-guided individualized PEEP optimization strategy, although larger multicenter studies are needed to determine effects on patient-centered outcomes.”

5. Mechanistic limitation of the study (EIT data not analyzed)

A central premise of the study is that EIT enables optimization of PEEP by balancing regional collapse and overdistension. However, the manuscript confirms that these EIT-derived variables were not systematically recorded for analysis.

This is a major limitation. Without quantitative EIT data, the study cannot substantiate the mechanistic pathway by which the intervention is presumed to act. Additionally, the absence of EIT acquisition in the control group prevents any direct comparison of regional ventilation patterns.As a result, the manuscript demonstrates a physiologic effect (improved oxygenation) but provides limited mechanistic insight into how this effect was achieved.

We thank the reviewer for this important comment. We agree that the absence of systematically recorded quantitative EIT-derived variables limits mechanistic interpretation. Although EIT was used clinically to guide individualized PEEP titration in the intervention group, quantitative EIT-derived indices were not systematically collected for formal analysis. In addition, EIT was not performed in the control group; therefore, direct comparison of regional ventilation patterns between groups was not possible.

We have revised the manuscript to explicitly acknowledge this limitation and to temper mechanistic interpretation. The revised text clarifies that the study demonstrates a physiologic effect, particularly improved oxygenation, but cannot definitively establish the mechanistic pathway by which this effect was achieved.

Revision made:

Discussion:

Attachment Submitted filename: PONE-D-26-05009R2. Response to Reviewers.docx

# Peer review: Decision Letter 2

**DOI:** 10.1371/journal.pone.0355609.r005

**Authors:** Roberta Ribeiro De Santis Santiago

5 Jul 2026

Dear Dr. Luu Quang,

Thank you for submitting your manuscript to PLOS ONE. After careful consideration, we feel that it has merit but does not fully meet PLOS ONE’s publication criteria as it currently stands. Therefore, we invite you to submit a revised version of the manuscript that addresses the points raised during the review process.

Please submit your revised manuscript by Aug 19 2026 11:59PM. If you will need more time than this to complete your revisions, please reply to this message or contact the journal office at plosone@plos.org. When you're ready to submit your revision, log on to https://www.editorialmanager.com/pone/ and select the 'Submissions Needing Revision' folder to locate your manuscript file.

- A letter that responds to each point raised by the academic editor and reviewer(s). You should upload this letter as a separate file labeled 'Response to Reviewers'.
- A marked-up copy of your manuscript that highlights changes made to the original version. You should upload this as a separate file labeled 'Revised Manuscript with Track Changes'.
- An unmarked version of your revised paper without tracked changes. You should upload this as a separate file labeled 'Manuscript'.

If you would like to make changes to your financial disclosure, please include your updated statement in your cover letter. Guidelines for resubmitting your figure files are available below the reviewer comments at the end of this letter.

If applicable, we recommend that you deposit your laboratory protocols in protocols.io to enhance the reproducibility of your results. Protocols.io assigns your protocol its own identifier (DOI) so that it can be cited independently in the future. For instructions see: https://journals.plos.org/plosone/s/submission-guidelines#loc-laboratory-protocols. Additionally, PLOS ONE offers an option for publishing peer-reviewed Lab Protocol articles, which describe protocols hosted on protocols.io. Read more information on sharing protocols at https://plos.org/protocols?utm_medium=editorial-email&utm_source=authorletters&utm_campaign=protocols.

As the corresponding author, your ORCID iD is verified in the submission system and will appear in the published article. PLOS supports the use of ORCID, and we encourage all coauthors to register for an ORCID iD and use it as well. Please encourage your coauthors to verify their ORCID iD within the submission system before final acceptance, as unverified ORCID iDs will not appear in the published article. *Only*  the individual author can complete the verification step; PLOS staff *cannot*  verify ORCID iDs on behalf of authors.

We look forward to receiving your revised manuscript.

Kind regards,

Roberta Ribeiro De Santis Santiago, M.D., Ph.D., R.R.T.

Academic Editor

PLOS One

Journal Requirements:

If the reviewer comments include a recommendation to cite specific previously published works, please review and evaluate these publications to determine whether they are relevant and should be cited. There is no requirement to cite these works unless the editor has indicated otherwise.

Please review your reference list to ensure that it is complete and correct. If you have cited papers that have been retracted, please include the rationale for doing so in the manuscript text, or remove these references and replace them with relevant current references. Any changes to the reference list should be mentioned in the rebuttal letter that accompanies your revised manuscript. If you need to cite a retracted article, indicate the article’s retracted status in the References list and also include a citation and full reference for the retraction notice.

Additional Editor Comments:

Dear Dr. Quang & authors, I hope this message finds you well.

I received 10 minutes ago the information that the 3 required reviewers completed their evaluation for the Second revision round.

Please see attached their comments.

In my end, I will prioritize the conclusion of this manuscript evaluation to the best of my abilities to make up for the delay we faced during this process.

I appreciate your time and patience.

[Note: HTML markup is below. Please do not edit.]

Reviewers' comments:

Reviewer's Responses to Questions

**Comments to the Author**

Reviewer #1: All comments have been addressed

Reviewer #2: All comments have been addressed

Reviewer #3: All comments have been addressed

**********

2. Is the manuscript technically sound, and do the data support the conclusions?

Reviewer #1: Yes

Reviewer #2: Yes

Reviewer #3: Partly

**********

3. Has the statistical analysis been performed appropriately and rigorously?

Reviewer #1: Yes

Reviewer #2: Yes

Reviewer #3: Yes

**********

4. Have the authors made all data underlying the findings in their manuscript fully available?

The PLOS Data policy

Reviewer #1: Yes

Reviewer #2: Yes

Reviewer #3: Yes

**********

5. Is the manuscript presented in an intelligible fashion and written in standard English?

Reviewer #1: Yes

Reviewer #2: Yes

Reviewer #3: Yes

**********

Reviewer #1: The authors made amendments accordingly. I have no more comments. Congratulations to all the authors.

Reviewer #2: No further comments.

Reviewer #3: check the attached file

This manuscript has improved substantially during the revision process and now represents a well-conducted randomized physiological study addressing an important clinical question. The remaining issues primarily concern interpretation rather than methodology.

I believe the manuscript is close to being suitable for publication after minor revision, mainly to further temper mechanistic conclusions and consistently frame the intervention as a bundled EIT-guided ventilation strategy rather than an evaluation of EIT technology alone.

**********

what does this mean?). If published, this will include your full peer review and any attached files.

If you choose “no”, your identity will remain anonymous but your review may still be made public.

**Do you want your identity to be public for this peer review?**  For information about this choice, including consent withdrawal, please see our Privacy Policy

Reviewer #1: **Yes:**  Changsheng Zhang

Reviewer #2: No

Reviewer #3: **Yes:**  Bruno Francisco de Freitas Tonelotto

**********

[NOTE: If reviewer comments were submitted as an attachment file, they will be attached to this email and accessible via the submission site. Please log into your account, locate the manuscript record, and check for the action link "View Attachments". If this link does not appear, there are no attachment files.]

To ensure your figures meet our technical requirements, please review our figure guidelines: https://journals.plos.org/plosone/s/figures

You may also use PLOS’s free figure tool, NAAS, to help you prepare publication quality figures: https://journals.plos.org/plosone/s/figures#loc-tools-for-figure-preparation.

NAAS will assess whether your figures meet our technical requirements by comparing each figure against our figure specifications.

Attachment Submitted filename: Reviewer Comments - PONE-D-26-05009.pdf

# Author comment: Author response to Decision Letter 3

**DOI:** 10.1371/journal.pone.0355609.r006

7 Jul 2026

Manuscript Number: [PONE-D-26-05009R2] - [EMID:e49ba8f50d8c88f0]

Response to Reviewers and Editors

Roberta Ribeiro De Santis Santiago, M.D., Ph.D., R.R.T.

Academic Editor

PLOS ONE

We thank you for the opportunity to revise and resubmit our manuscript entitled “Electrical impedance tomography–guided individualized ventilation strategy in patients with trauma-related and postoperative acute respiratory distress syndrome.”

We sincerely appreciate the time and effort invested by the editor and reviewers in evaluating our work. Their insightful comments and constructive suggestions have been invaluable in improving the clarity and rigor of the manuscript. We have carefully addressed all comments and revised the manuscript accordingly. A revised version with tracked changes is provided, and detailed point-by-point responses are presented below, with our replies indicated in blue.

Response to Reviewer #1

We sincerely thank Reviewer #1 for confirming that all previous comments have been addressed. We greatly appreciate the reviewer’s constructive feedback throughout the revision process, which has helped improve the clarity and presentation of the manuscript.

Response to Reviewer #2

We sincerely thank Reviewer #2 for the positive reassessment and for indicating that there are no further comments. We appreciate the reviewer’s time and valuable input during the review process.

Response to Reviewer #3

We sincerely thank the reviewer for the careful reassessment of our manuscript and for the thoughtful, constructive comments. We greatly appreciate the recognition that the manuscript has improved substantially in methodological transparency, statistical rigor, and interpretation of findings, and that the remaining concerns primarily relate to interpretation rather than methodology. In response, we have revised the manuscript to consistently present the intervention as an EIT-guided individualized ventilation strategy, clarified its multicomponent nature, further tempered mechanistic interpretations, expanded the discussion of mechanical power, clarified statistical model diagnostics, and revised the Conclusions to emphasize physiological feasibility rather than superiority of EIT as a monitoring modality. Detailed point-by-point responses are provided below.

Major comments

1. The intervention should consistently be presented as a bundled ventilation strategy rather than an isolated EIT intervention.

Although the Discussion has been revised to acknowledge this issue, the manuscript occasionally continues to imply that the observed physiological benefits resulted directly from EIT guidance. In reality, the intervention consisted of multiple simultaneous components, including recruitment maneuvers, decremental PEEP titration, repeated reassessment, individualized PEEP selection, and EIT monitoring. Consequently, the study compares two ventilation strategies rather than evaluating the independent effect of EIT itself. The wording throughout the manuscript should consistently reflect this distinction. Expressions such as “EIT-guided individualized ventilation strategy” or “EIT-guided PEEP optimization strategy” would more accurately describe the intervention than suggesting an isolated effect of EIT.

We thank the reviewer for this important comment. We fully agree that the intervention should be interpreted as a multicomponent ventilation strategy rather than as an isolated evaluation of EIT monitoring. In response, we revised the manuscript throughout to use “EIT-guided individualized ventilation strategy” when referring to the overall intervention. When referring specifically to the PEEP component, we used “EIT-based PEEP titration,” “individualized PEEP selection,” or “protocol-selected PEEP,” as appropriate.

We also clarified that the trial compares ventilation strategies and does not establish the independent effect of EIT monitoring alone. The intervention components are now explicitly described as EIT monitoring, recruitment maneuvers, decremental PEEP titration, repeated reassessment, and individualized PEEP selection.

Revision made:

Title: “Electrical impedance tomography–guided individualized ventilation strategy in patients with trauma-related and postoperative acute respiratory distress syndrome”

Abstract (Methods): “In this single-center randomized controlled trial, 86 adults with moderate-to-severe trauma- or postoperative-associated ARDS were randomized to an EIT-guided individualized ventilation strategy (n = 43) or a lower-PEEP/FiO2 strategy (n = 43).”

Abstract (Methods): “In the EIT group, the multicomponent strategy included EIT monitoring, recruitment maneuvers, decremental PEEP titration, repeated reassessment, and individualized PEEP selection based on the intersection of regional collapse and overdistension curves.”

Methods (EIT group): “The EIT-guided individualized ventilation strategy comprised EIT monitoring, recruitment maneuvers, decremental PEEP titration, repeated reassessment, and individualized PEEP selection.”

Results: “Protocol adherence was high in the intervention arm: all analyzed patients underwent initial PEEP titration within the EIT-guided individualized ventilation strategy on Day 0, and repeat titration was performed on Days 1–3 in all patients with available follow-up.”

Discussion: “The intervention incorporated EIT monitoring, recruitment maneuvers, decremental PEEP titration, repeated reassessment, and individualized PEEP selection.”

Limitations: “Therefore, the observed oxygenation benefit should be interpreted as the effect of the multicomponent EIT-guided individualized ventilation strategy rather than as evidence of a specific EIT-derived mechanism.”

2. Mechanistic interpretation remains limited.

The principal conceptual limitation of the study persists. The proposed mechanism underlying the intervention is optimization of the balance between regional collapse and overdistension. However, quantitative EIT-derived variables were not systematically recorded, and EIT was not performed in the control group. Therefore, although the study convincingly demonstrates improved oxygenation, it cannot establish whether this resulted from improved regional recruitment, reduced overdistension, improved ventilation-perfusion matching, higher PEEP exposure, recruitment maneuvers, or the combined effects of the bundled intervention. The current Discussion acknowledges this limitation but still occasionally implies mechanistic conclusions that are not directly supported by the available data. I would recommend making the mechanistic interpretation even more conservative.

We thank the reviewer for this important and insightful comment. We agree that mechanistic interpretation should be more conservative. We further revised the Discussion, Limitations, and Conclusions to state explicitly that the mechanism underlying the oxygenation improvement cannot be determined from the present data because quantitative EIT-derived variables were not systematically collected and EIT was not performed in the control group.

The revised manuscript no longer attributes the oxygenation benefit to a specific EIT-derived mechanism. Instead, we explicitly acknowledge that the present design cannot distinguish among potential contributors, including regional recruitment, reduced overdistension, ventilation-perfusion matching, greater PEEP exposure, recruitment maneuvers, or the combined effects of the multicomponent intervention strategy. We also removed or tempered wording that could imply direct confirmation of regional ventilation mechanisms.

Revision made:

Discussion: “Because this trial evaluated a bundled EIT-guided ventilation strategy, without systematic collection of quantitative EIT-derived indices or EIT assessment in the control group, it cannot determine the mechanisms underlying the observed improvement in oxygenation or the relative contribution of individual intervention components.”

Discussion: “These features may partly explain the oxygenation improvement observed with the EIT-guided individualized ventilation strategy and differences from prior studies in more heterogeneous or predominantly medical ARDS cohorts, although the underlying mechanism could not be determined.”

Discussion: “Although quantitative indices of collapse and overdistension were not analyzed, PEEP titration within the intervention strategy was guided by real-time EIT assessment of regional ventilation.”

Limitations: “Finally, mechanistic interpretation was limited because EIT was not performed in the control group and quantitative EIT-derived indices were not systematically collected. Therefore, the observed oxygenation benefit should be interpreted as the effect of the multicomponent EIT-guided individualized ventilation strategy rather than as evidence of a specific EIT-derived mechanism.”

Conclusions: “These findings suggest the physiological feasibility of this strategy, but do not establish the independent effect of EIT monitoring or the mechanistic pathway underlying the observed oxygenation benefit.”

3. Discussion of mechanical power could be strengthened.

The authors appropriately report that mechanical power normalized to predicted body weight was modestly higher in the intervention group despite similar driving pressures. However, the physiological interpretation remains relatively superficial. Mechanical power is influenced by several interacting determinants, including tidal volume, respiratory rate, inspiratory flow, PEEP, and driving pressure. Because driving pressure remained unchanged while PEEP was consistently higher in the intervention group, it is likely that the observed increase in MP/PBW primarily reflects greater PEEP exposure rather than worsening respiratory mechanics. Expanding this discussion would improve the physiological interpretation and place the findings in the context of current concepts regarding mechanical power and ventilator-induced lung injury.

We thank the reviewer for this helpful comment. We agree that the physiological interpretation of MP/PBW required expansion. We revised the Discussion to clarify that mechanical power integrates several ventilatory determinants, including tidal volume, respiratory rate, inspiratory flow, driving pressure, and PEEP. We now interpret the modestly higher MP/PBW in the EIT group in relation to greater PEEP exposure within the EIT-guided individualized ventilation strategy, while noting that plateau pressure and driving pressure were not increased.

The revised Discussion also places this finding in the context of the MP framework proposed by Gattinoni et al., the analysis by Costa et al. showing that driving pressure and respiratory rate capture much of the prognostic information contained in mechanical power, and EIT-based evidence showing that recruitable alveolar collapse and regional hyperdistension can be estimated at the bedside during decremental PEEP titration. We further clarified that, because quantitative EIT-derived indices were not systematically collected and EIT was not performed in the control group, the findings remain physiologic and hypothesis-generating.

Revision made:

Discussion: “MP/PBW decreased over time in both groups, with no group-by-time interaction, indicating similar temporal trends. Overall MP/PBW was modestly higher in the EIT group.”

Discussion: “In the MP framework proposed by Gattinoni et al., MP integrates the ventilator-related load generated by VT, RR, inspiratory flow, Pdriv, and PEEP (5).”

Discussion: “In our study, higher MP/PBW paralleled greater PEEP exposure within the EIT-guided individualized ventilation strategy, while Pplat and Pdriv were not increased. This pattern supports a PEEP-related contribution to calculated MP/PBW rather than worsening respiratory mechanics or greater intrinsic lung injury.”

Discussion: “In an analysis of patients with ARDS, Costa et al. showed that MP was associated with outcome, but a simpler model using ΔP and RR captured comparable prognostic information, with ΔP having a greater effect than RR (37).”

Discussion: “In an EIT-based study during decremental PEEP titration, Costa et al. showed that recruitable alveolar collapse and regional hyperdistension can be estimated at the bedside and have regional distributions (7). This supports the concept that PEEP effects depend on lung morphology and recruitability (38).”

Discussion: “Because quantitative EIT-derived indices were not systematically collected and EIT was not performed in the control group, our findings should be interpreted as physiologic and hypothesis-generating, not as evidence of increased injurious regional lung stress or a specific EIT-derived mechanism.”

4. The Conclusions should remain slightly more conservative.

The Conclusions have improved substantially compared with previous versions. Nevertheless, I would recommend emphasizing that the study demonstrates the physiological feasibility of an EIT-guided ventilation strategy rather than establishing superiority of EIT as a monitoring modality. Future studies incorporating quantitative EIT-derived measurements and parallel monitoring in both groups will be required to define the mechanistic pathways responsible for the observed physiological benefits.

We thank the reviewer for this important recommendation. We agree and have revised both the Abstract Conclusions and the main Conclusions to emphasize physiological feasibility rather than superiority of EIT as a monitoring modality. The revised conclusions explicitly state that the study does not establish the independent effect of EIT monitoring or the mechanistic pathway underlying the observed oxygenation benefit.

We also added that future multicenter studies should include quantitative EIT-derived measurements and parallel regional ventilation monitoring in both groups to clarify mechanisms and determine effects on patient-centered outcomes.

Revision made:

Abstract (Conclusions): “These findings suggest the physiological feasibility of this strategy, but do not establish the independent effect of EIT monitoring or the underlying mechanistic pathway. Larger multicenter studies are needed to determine mechanisms and effects on patient-centered outcomes.”

Conclusions: “These findings suggest the physiological feasibility of this strategy, but do not establish the independent effect of EIT monitoring or the mechanistic pathway underlying the observed oxygenation benefit. Larger multicenter studies incorporating quantitative EIT-derived measurements and parallel regional ventilation monitoring in both groups are needed to determine effects on patient-centered outcomes.”

Minor comments

1. Consider using consistent terminology throughout the manuscript. “EIT-guided individualized ventilation strategy” is scientifically more accurate than alternating between “EIT-guided ventilation” and “EIT-guided PEEP.”

We thank the reviewer for this helpful comment. We agree that “EIT-guided individualized ventilation strategy” more accurately describes the intervention than alternating between “EIT-guided ventilation” and “EIT-guided PEEP.” We have therefore revised the manuscript to use “EIT-guided individualized ventilation strategy” when referring to the overall intervention. When referring specifically to the PEEP component, we use “EIT-based PEEP titration,” “individualized PEEP selection,” or “protocol-selected PEEP,” as appropriate. Group labels such as “EIT group” and “control group” were retained where appropriate for tables, figure legends, and concise reporting of group comparisons.

Revision made:

Terminology was revised throughout the title, Abstract, Introduction, Methods, Results, Discussion, Clinical implications, and Conclusions to frame the intervention as an EIT-guided individualized ventilation strategy rather than as an isolated EIT intervention or EIT-guided PEEP alone.

2. The Discussion could benefit from a more detailed integration of recent literature regarding regional lung stress, recruitability, and mechanical power, particularly work from Costa, Gattinoni, and colleagues.

We thank the reviewer for this helpful suggestion. We revised the Discussion to better integrate recent literature on regional lung heterogeneity, recruitability, mechanical power, and ventilator-induced lung injury. The revised text n

Attachment Submitted filename: Response to Reviewers R3.docx

# Peer review: Decision Letter 3

**DOI:** 10.1371/journal.pone.0355609.r007

**Authors:** Roberta Ribeiro De Santis Santiago

24 Jul 2026

Electrical impedance tomography–guided individualized ventilation strategy in patients with trauma-related and postoperative acute respiratory distress syndrome

PONE-D-26-05009R3

Dear Dr. Luu Quang,

We’re pleased to inform you that your manuscript has been judged scientifically suitable for publication and will be formally accepted for publication once it meets all outstanding technical requirements.

Within one week, you’ll receive an e-mail detailing the required amendments. When these have been addressed, you’ll receive a formal acceptance letter and your manuscript will be scheduled for publication.

An invoice will be generated when your article is formally accepted. Please note, if your institution has a publishing partnership with PLOS and your article meets the relevant criteria, all or part of your publication costs will be covered. Please make sure your user information is up-to-date by logging into Editorial Manager at Editorial Manager® and clicking the ‘Update My Information' link at the top of the page. For questions related to billing, please contact billing support.

If your institution or institutions have a press office, please notify them about your upcoming paper to help maximize its impact. If they’ll be preparing press materials, please inform our press team as soon as possible -- no later than 48 hours after receiving the formal acceptance. Your manuscript will remain under strict press embargo until 2 pm Eastern Time on the date of publication. For more information, please contact onepress@plos.org.

Kind regards,

Roberta Ribeiro De Santis Santiago, M.D., Ph.D., R.R.T.

Academic Editor

PLOS One

Additional Editor Comments (optional):

Dear authors, I am happy that your manuscript was accepted for publication and I appreciate your patience with the delays during this review process.

Reviewers' comments:

Reviewer's Responses to Questions

**Comments to the Author**

Reviewer #3: All comments have been addressed

**********

2. Is the manuscript technically sound, and do the data support the conclusions?

Reviewer #3: Yes

**********

3. Has the statistical analysis been performed appropriately and rigorously?

Reviewer #3: Yes

**********

4. Have the authors made all data underlying the findings in their manuscript fully available?

The PLOS Data policy

Reviewer #3: Yes

**********

5. Is the manuscript presented in an intelligible fashion and written in standard English?

Reviewer #3: Yes

**********

Reviewer #3: I appreciate the opportunity to review the revised version of this manuscript entitled "Electrical Impedance Tomography–Guided Individualized Ventilation Strategy in Patients with Trauma-Related and Postoperative Acute Respiratory Distress Syndrome." The authors have been highly responsive throughout the review process, and the manuscript has improved substantially after multiple rounds of revision.

Recommendation: Accept after Minor Editorial Revision.

**********

what does this mean?). If published, this will include your full peer review and any attached files.

If you choose “no”, your identity will remain anonymous but your review may still be made public.

**Do you want your identity to be public for this peer review?**  For information about this choice, including consent withdrawal, please see our Privacy Policy

Reviewer #3: **Yes:**  Bruno Tonelotto

**********

Attachment Submitted filename: Reviewer coments - PONE-D-26-05009R3.pdf

# Editor report: Acceptance letter

**DOI:** 10.1371/journal.pone.0355609.r008

**Authors:** Roberta Ribeiro De Santis Santiago

PONE-D-26-05009R3

PLOS One

Dear Dr. Luu Quang,

I'm pleased to inform you that your manuscript has been deemed suitable for publication in PLOS One. Congratulations! Your manuscript is now being handed over to our production team.

At this stage, our production department will prepare your paper for publication. This includes ensuring the following:

* All references, tables, and figures are properly cited

* All relevant supporting information is included in the manuscript submission,

* There are no issues that prevent the paper from being properly typeset

You will receive further instructions from the production team, including instructions on how to review your proof when it is ready. Please keep in mind that we are working through a large volume of accepted articles, so please give us a few days to review your paper and let you know the next and final steps.

Lastly, if your institution or institutions have a press office, please let them know about your upcoming paper now to help maximize its impact. If they'll be preparing press materials, please inform our press team within the next 48 hours. Your manuscript will remain under strict press embargo until 2 pm Eastern Time on the date of publication. For more information, please contact onepress@plos.org.

You will receive an invoice from PLOS for your publication fee after your manuscript has reached the completed accept phase. If you receive an email requesting payment before acceptance or for any other service, this may be a phishing scheme. Learn how to identify phishing emails and protect your accounts at https://explore.plos.org/phishing.

If we can help with anything else, please email us at customercare@plos.org.

Thank you for submitting your work to PLOS ONE and supporting open access.

Kind regards,

PLOS ONE Editorial Office Staff

on behalf of

Dr. Roberta Ribeiro De Santis Santiago

Academic Editor

PLOS One
